# PPA1 promotes oxidative phosphorylation and malignant progression of colorectal cancer under glucose restriction via AMPK/ULK1/FUNDC1-mediated mitophagy

**DOI:** 10.1038/s41420-025-02816-y

**Published:** 2025-11-28

**Authors:** Yajun Chen, Qican Deng, Zhenzhou Chen, Lei Yang, Xiang Li, Zhongxue Fu

**Affiliations:** 1 Department of Gastrointestinal Surgery, The Third Affiliated Hospital of Chongqing Medical University, Chongqing, China; 2 Department of Gastrointestinal Surgery, The First Affiliated Hospital of Chongqing Medical University, Chongqing, China

**Keywords:** Colon cancer, Cancer metabolism

## Abstract

Metabolic reprogramming is a hallmark of colorectal cancer (CRC). Pyrophosphatase 1(PPA1), an energy-metabolizing enzyme, has been observed to be upregulated in multiple cancers and implicated in tumorigenesis and progression. However, its specific role in metabolic rewiring of CRC and the underlying molecular mechanisms remain poorly understood. Our study revealed that PPA1 is highly expressed in CRC epithelial cells and is significantly associated with advanced tumor size, lymph node status, TNM stage, and reduced overall survival in patients. Knockdown of PPA1 suppressed CRC tumorigenesis and metastasis both in vitro and in vivo. Under glucose-restricted conditions, PPA1 depletion impaired OXPHOS in CRC cells, leading to reduced oxygen consumption, decreased ATP production, elevated mitochondrial ROS levels, and decline in mitochondrial membrane potential. Mechanistically, PPA1 promotes phosphorylation of AMPK at Thr172, thereby facilitating phosphorylation of ULK1 at Ser467 and Ser555, and subsequently enhancing FUNDC1 phosphorylation at Ser17. This phosphorylation cascade initiates mitophagy to sustain OXPHOS metabolic activity, thereby driving CRC malignant progression. In summary, PPA1 sustains OXPHOS and drives malignant progression in CRC under glucose restriction by promoting AMPK/ULK1/FUNDC1-mediated mitophagy.

## Background

Colorectal cancer (CRC), the third most prevalent malignancy and second leading cause of cancer-related deaths globally, is the most common gastrointestinal cancer [1]. Despite advancements in multimodal therapies—including neoadjuvant treatment, radical surgery, adjuvant chemoradiotherapy, and immunotherapy—the 5 year survival rates for stage III and IV CRC remain critically low at 17% and 14%, respectively [2–4]. These sobering statistics underscore the urgent need to unravel the complex mechanisms driving CRC progression and identify novel therapeutic targets.

Inorganic pyrophosphatase 1(PPA1), an essential metabolic enzyme, is overexpressed in lung adenocarcinoma, invasive ductal carcinoma, prostate cancer, gastric cancer, hepatocellular carcinoma, diffuse large B-cell lymphoma, ovarian cancer, and CRC [5]. In vitro PPA1 silencing upregulates cell cycle inhibitors (p21, TP53), suppressing proliferation and inducing apoptosis in lung, breast, and lymphoma models [6]. In CRC, PPA1 knockdown attenuates JNK dephosphorylation and PI3K phosphorylation, impairing proliferative capacity [7, 8]. Similarly, PPA1 depletion in non-small cell lung cancer promotes apoptosis via p-JNK1 dephosphorylation [9]. Beyond oncogenesis, PPA1 regulates metabolic homeostasis: PPA1 knockout mice develop glucose intolerance and insulin resistance under high-fat diets [10], while yeast PPA1 silencing disrupts NAD+ metabolism, triggering cell cycle arrest and autophagic death [11, 12]. PPA1 is also known to promote early-stage adipocyte differentiation and regulate adipose tissue development and systemic metabolic homeostasis [13]. However, whether PPA1 directly influences tumor metabolism and the precise mechanisms underlying its potential oncogenic effects remain poorly understood.

Historically, the Warburg effect—characterized by suppressed oxidative phosphorylation (OXPHOS) and enhanced glycolysis—dominated the understanding of cancer metabolism [14]. However, emerging evidence reveals that mitochondrial respiration remains functional in many tumors, with OXPHOS activity upregulated in certain cancers to fuel proliferation, metastasis, and chemoresistance [15–17]. In CRC, OXPHOS serves as the primary ATP source, and its inhibition suppresses tumor growth and metastasis [18–20]. Notably, CRC cells with elevated OXPHOS and mitochondrial fitness exhibit resistance to 5-fluorouracil (5-FU) and antimycin A, promoting aggressive phenotypes [21]. OXPHOS upregulation further drives stemness and epithelial-mesenchymal transition (EMT) in chemoresistant cells, while OXPHOS inhibition restores 5-FU sensitivity [22]. Mitophagy, the selective autophagic clearance of damaged mitochondria, is critical for maintaining mitochondrial quality and OXPHOS. Dysfunctional mitochondria are encapsulated into autophagosomes and degraded via lysosomes [23]. Under metabolic stress (hypoxia, nutrient deprivation), mitophagy reduces mitochondrial ROS, sustains cellular homeostasis, and inhibits apoptosis [24]. In cancer stem cells, mitophagy enhances plasticity, chemoresistance, and survival [25–27]. Despite the recognized role of mitochondrial metabolism in tumor bioenergetics, the interplay between mitophagy and OXPHOS in CRC remains poorly understood.

FUNDC1-mediated mitophagy, regulated through direct activation by Unc-51-like autophagy-activating kinase 1(ULK1), plays a critical role in tumorigenesis. Hydrogen peroxide upregulates FUNDC1 expression via ERK1/2 signaling, triggering mitophagy to promote laryngeal cancer progression [28]. Additionally, FUNDC1-associated mitochondria-associated membranes (MAMs) facilitate calcium flux into the cytosol, leading to calcineurin-dependent dephosphorylation of nuclear factor of activated T cells 1 (NFAT1) and subsequent upregulation of the oncogene Bmi1, thereby driving breast cancer progression [29]. In hepatocellular carcinoma (HCC), FUNDC1-mediated mitophagy suppresses early tumor initiation by inhibiting mtDNA/mtROS-induced inflammasome activation, while its upregulation in advanced stages may paradoxically support tumor growth [30]. However, the role of FUNDC1-mediated mitophagy in colorectal cancer has not yet been fully elucidated.

Our study identifies PPA1 as a critical mediator of CRC adaptation to nutrient stress. PPA1 is overexpressed in epithelial cells of CRC tissue and correlates with patient survival. Under glucose restriction, PPA1 facilitates AMPK phosphorylation at Thr172 and ULK1 phosphorylation at Ser467/Ser556, activating FUNDC1-mediated protective mitophagy. This process sustains OXPHOS activity, providing bioenergetic support for CRC proliferation, migration, and invasion. These findings highlight PPA1 as a novel therapeutic target for CRC, particularly in nutrient-deprived microenvironments.

## Results

### PPA1 is overexpressed in epithelial cells of CRC and correlates with patient prognosis

**Fig. 1:**
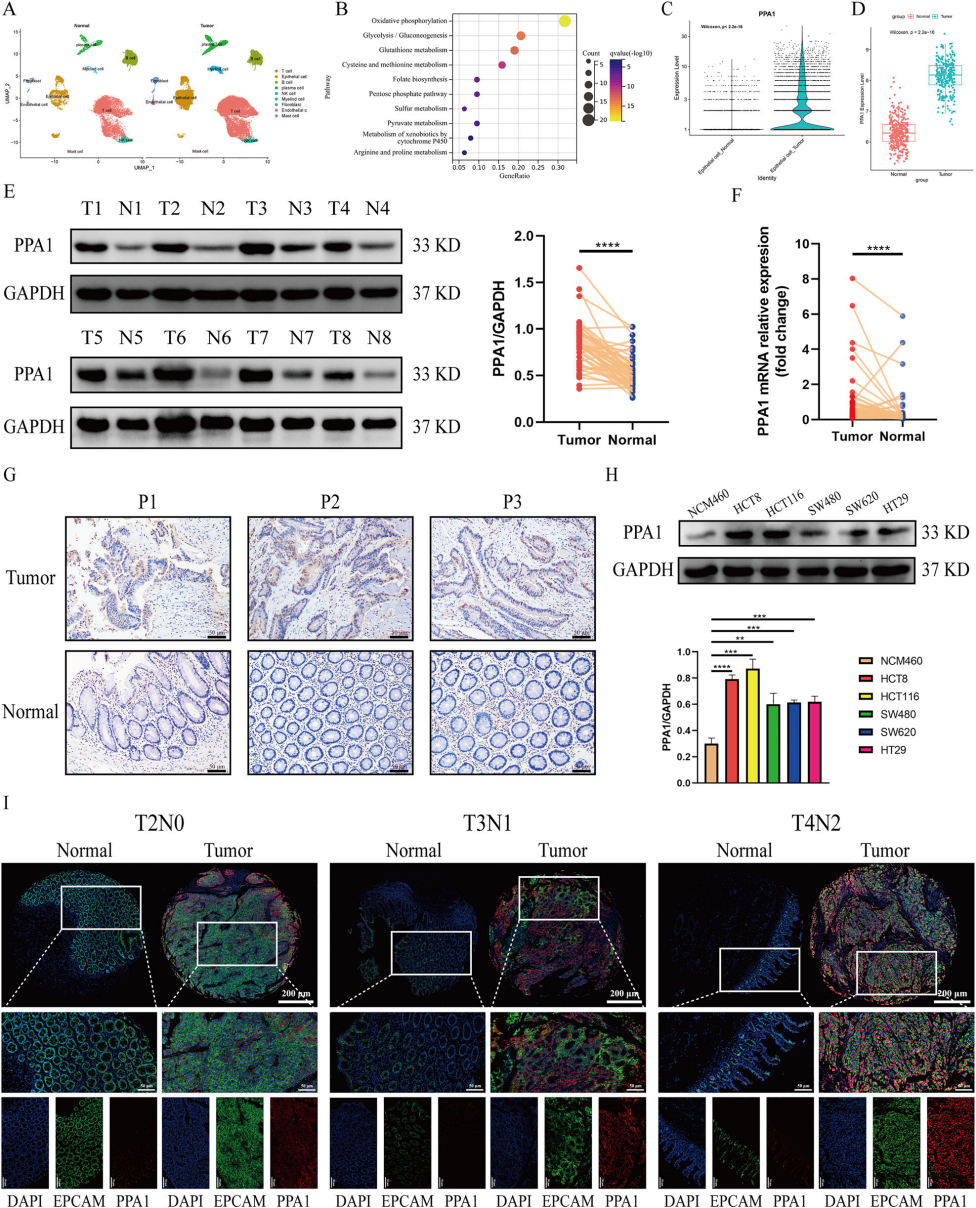
The highly expression of PPA1 in epithelial cells of CRC. **A** UMAP plot displays the 9 major cell clusters in CRC and adjacent normal tissues from the GSE200997 dataset; **B** Bubble plot highlights the top 10 enriched metabolic pathways among 63 metabolism-related DEGs; **C** Violin plot compares PPA1 expression in epithelial cells between CRC and adjacent normal tissues. **D** Western blotting analysis of PPA1 protein expression in CRC and adjacent normal tissues. **E** Expression of PPA1 in 288 CRC tissues from the TCGA database and 308 normal colon tissues from the GTEx database. **F** The mRNA expression levels of PPA1 in 48 paired CRC and adjacent normal tissues, normalized to β-actin and analyzed relative to the control group. **G** Immunohistochemical staining of PPA1 expression in CRC and adjacent normal tissues (200× magnification). **H** The expression levels of PPA1 protein in Normal intestinal epithelial cell line-NCM460 and CRC cell lines—HCT8, HCT116, HT29, SW480 and SW620 are evaluated by Western blotting (Mean ± SD, *n* = 3). **I** Immunofluorescence staining of PPA1 expression in epithelial cells (EpCAM + ; upper panel: 50×, lower panel: 200× magnification) between CRC and adjacent tissues. **p < 0.01, ***p < 0.001, ****p < 0.0001.

To explore tumor-specific transcriptional alterations between CRC cells and normal intestinal epithelial cells, we obtained the GSE200997 dataset from the GEO database, encompassing scRNA-seq data from 16 CRC tissues and 7 paired adjacent normal tissues. Following rigorous quality control (cells with>100 and <4000 measured genes, cells with <25% mitochondrial contamination) (Fig. S1A), we performed dimensionality reduction via PCA and UMAP, stratifying cells into nine distinct clusters (Fig. 1A). Cluster annotation was validated using canonical markers: B cells (*CD79A*, *CD19)*, Plasma cells (*CD79A*, *MZB1*, *IGHA1)*, T cells (*CD3D*, *CD3E*, *IL7R*), NK cells (*GNLY*, *KLRD1*, *NKG7*), Endothelial cells (*PECAM1*, *VWF*, *CD34*), Epithelial cells (*EPCAM*, *KRT8*), Fibroblasts (*ACTA2*, *COL1A1*, *COL1A2*), Myeloid cells (*CD14*, *LYZ*, *CD68*, *CD163*), Mast cells (*KIT*, *MS4A2*)(Fig. S1B) [31]. The “FindAllMarkers” function was employed to explore the DEGs between epithelial cells of CRC tissues and epithelial cells of adjacent normal tissues and a total of 437 DEGs met the thresholds of |log2foldchange | > 0.585 and adjusted *p*-value < 0.05 (Fig. S1C). To delineate metabolic reprogramming in CRC cells, we performed KEGG enrichment analyses using the “clusterProfiler” R package on 63 metabolism-associated DEGs. Strikingly, 20 DEGs were enriched in OXPHOS pathway, with majority demonstrating elevated expression in tumor-derived epithelial cells (Fig. 1B, Fig. S1D), which contrary to canonical Warburg effect [32]. Moreover, among the 20 OXPHOS-associated DEGs, *PPA1* emerged as the most significantly upregulated in epithelial cells of CRC tissues compared to epithelial cells of adjacent normal tissues (Fig. 1C, Fig. S1D, E). Simultaneously, analysis of bulk-RNAseq data from the TCGA and GTEx databases revealed significantly higher PPA1 expression in colorectal cancer (CRC) tissues compared to normal colon tissues (Fig. 1D).

To further validate expression differences of PPA1 between CRC and adjacent normal tissues, we collected 48 paired CRC and adjacent normal tissues. The results of Western blotting and qPCR demonstrated that expression of PPA1 significant upregulated in CRC tissues versus matched adjacent normal tissues (Fig. 1E, F). In addition, immunohistochemical staining showed that more epithelial cells in CRC were positive for PPA1 compared with matched adjacent normal tissues (Fig. 1G). Next, Western blotting was used to detect the protein expression levels of PPA1 in normal intestinal epithelial cells (NCM460) and CRC cell lines (HCT8, HT116, SW480, SW620, and HCT29). As shown in Fig. 1H, all the expressions of PPA1 in the five CRC cells were remarkably higher than that in NCM460 cells.

Finally, to precisely validate the expression of PPA1 in epithelial cells of CRC and its correlation with clinical characteristics of CRC patients, we obtained a tissue microarray (TMA) containing 80 pairs of CRC and adjacent normal tissues. Dual immunofluorescence co-staining using EPCAM as an epithelial lineage marker was performed to evaluate PPA1 expression in epithelial cells of CRC. As shown in Fig. 1I and Fig. S1F, the mean fluorescence intensity (MFI) of PPA1 in epithelial cells of CRC tissues was significantly higher than that in the adjacent normal tissues. Patients with CRC were stratified into PPA1-high and PPA1-low groups based on the median expression level of PPA1. Combined analysis of PPA1 expression and the clinical data revealed that the CRC patients in PPA1-high group exhibited higher T stage (*p* = 0.0033; Fig. S1G), lymph node metastasis rate (*p* < 0.0001; Fig. S1H), and TNM stage (*p* < 0.0001; Fig. S1I), whereas PPA1 expression showed no correlation with age (*p* = 0.2318), gender (*p* = 0.2337), or M stage (*p* = 0.2862) of patients (Table S2). Kaplan-Meier survival analysis indicated that the overall survival rate of CRC patients in the PPA1-high group was significantly lower than that in the PPA1-low group (Fig. S1J).

In summary, we found that PPA1 was highly expressed in epithelial cells of CRC patients and it was associated with the survival rate of CRC patients. Therefore, we believe that PPA1 affects the malignant behavior of CRC and may be a potential target for the treatment of CRC patients.

### PPA1 promotes the proliferation, migration and invasion of CRC cells

To investigate the effects of PPA1 on the biological functions of CRC cells, we transfected HCT8 and HCT116 cells, which had the highest expression of PPA1, with ShRNA knockdown lentivirus for PPA1 and screened three stable cell lines including the control group (Sh-NC) and the knockdown group (Sh1-PPA1, Sh2-PPA1) cell lines.

**Fig. 2:**
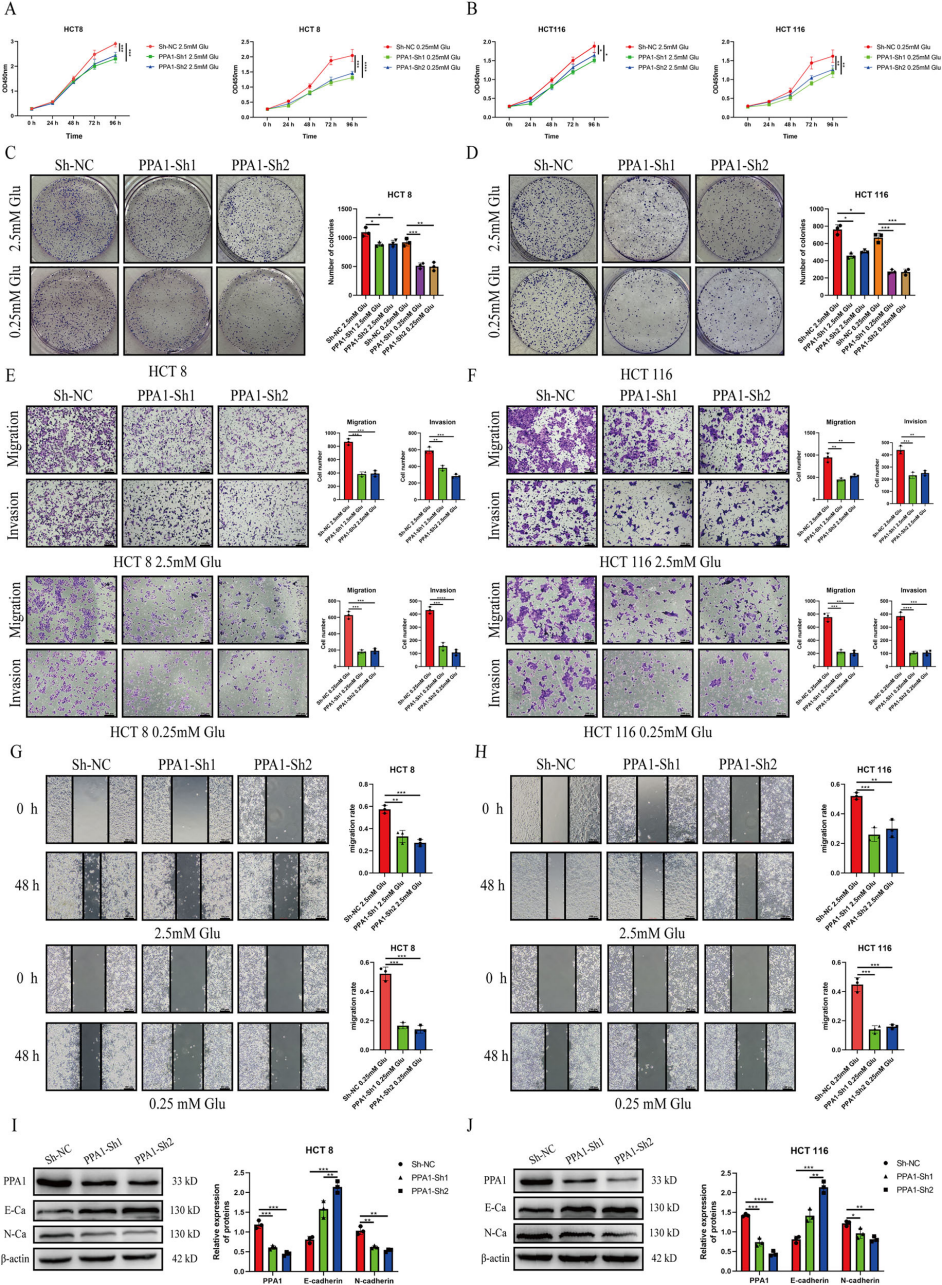
Effects of PPA1 knockdown on proliferation, invasion, and migration abilities of CRC cells. **A**, **B** CCK-8 assay assessing the impact of PPA1 knockdown on the proliferative capacity of HCT8 cells (**A**) and HCT116 cells (**B**); (**C**, **D**) Colony formation assay demonstrating the effect of PPA1 knockdown on the clonogenic ability of HCT8 cells (**C**) and HCT116 cells (**D**); (**E**, **F**) Transwell assay evaluating the migratory and invasive abilities of HCT8 cells (**E**) and HCT116 cells (**F**) following PPA1 knockdown(100× magnification); (**G**, **H**) Wound healing assay illustrating the influence of PPA1 knockdown on the migratory capacity of HCT8 cells (**G**) and HCT116 cells (**H**) (100× magnification); (**I, J**) Western blotting analysis of PPA1, E-cadherin, and N-cadherin expression levels in HCT8 cells (**I**) and HCT116 cells (**J**) after PPA1 knockdown. All data are presented as Mean ± SD, *n* = 3. *p < 0.05, **p < 0.01, ****p < 0.0001.

Through CCK-8 and colony formation assays, we found that PPA1 knockdown significantly inhibited the proliferation and colony-forming ability of CRC cells compared to control group (Fig. 2A–D). Furthermore, under glucose restriction condition (0.25 mM glucose), PPA1 knockdown more strongly suppressed these effects (Fig. S2A, B). To investigate the metastatic potential of CRC cells following PPA1 knockdown, we performed Transwell migration and invasion assays. As shown in Fig. 2E, F, the number of migrating and invading cells in the Sh1-PPA1 and Sh2-PPA1 groups was significantly less than that in the Sh-NC group. At the same time, the wound healing assay further revealed that compared with the Sh-NC group, the migration rates of cells in the Sh1-PPA1 and Sh2-PPA1 groups were significantly decreased (Fig. 2G, H). Consistently, under glucose-restricted conditions, PPA1 knockdown further suppressed the migratory and invasive abilities of CRC cells (Fig. S2C–E). Finally, we verified the expressions of E-cadherin and N-cadherin, two EMT markers, in HCT8 and HCT116 cells by Western blotting. As shown in Fig. 2I, J, following the knockdown of PPA1, E-cadherin expression was upregulated markedly, whereas N-cadherin expression was downregulated significantly. These findings collectively demonstrate that PPA1 silencing attenuates proliferation, suppresses metastatic behaviors, and reverses EMT progression of CRC cell.

**Fig. 3:**
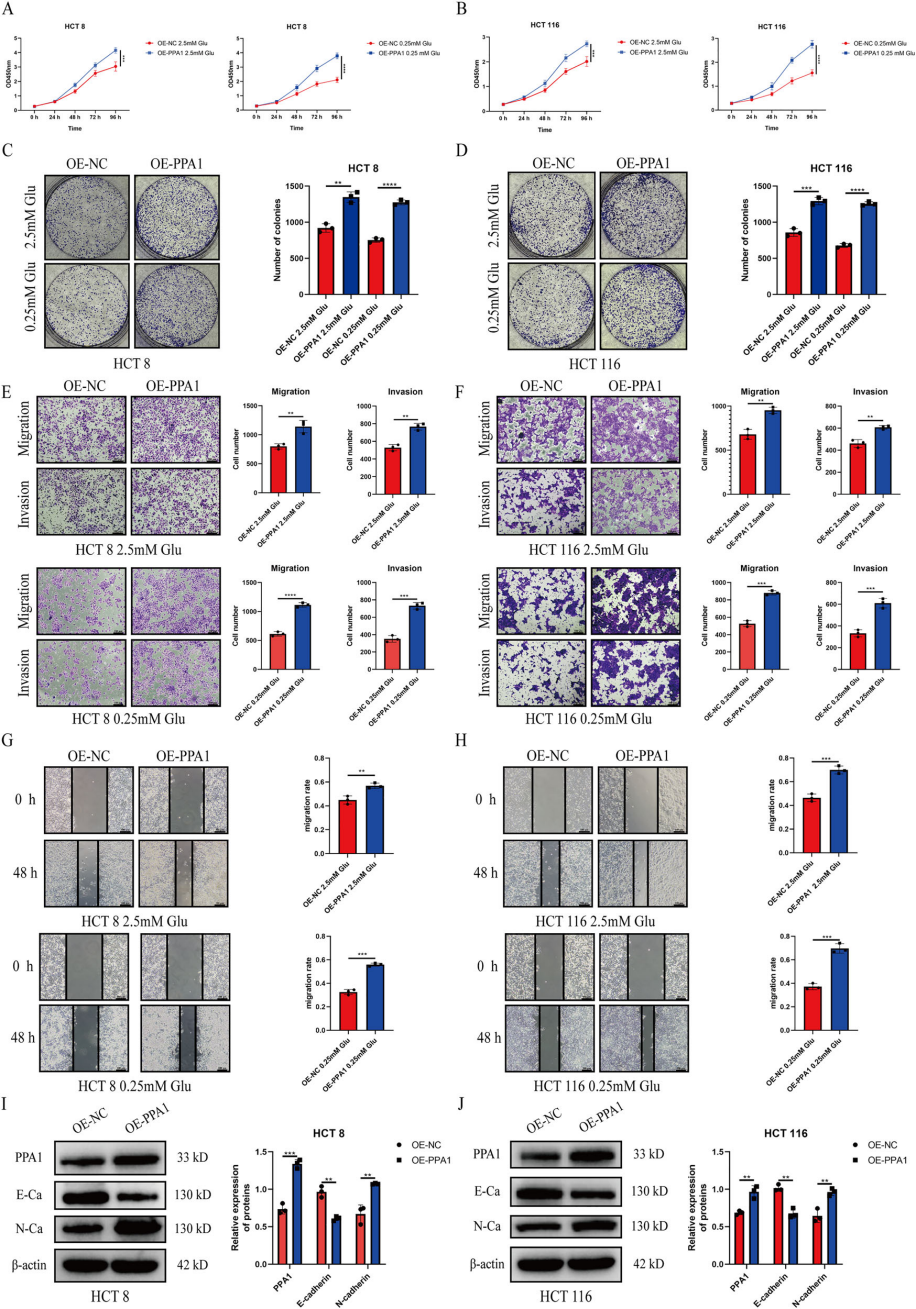
Effects of PPA1 overexpression on proliferation, invasion, and migration abilities of CRC cells. **A**, **B** CCK-8 assay evaluating the impact of PPA1 overexpression on the proliferative capacity of HCT8 cells (**A**) and HCT116 cells (**B**). (**C**, **D**) Colony formation assay demonstrating the effect of PPA1 overexpression on the clonogenic ability of HCT8 cells (**C**) and HCT116 cells (**D**); (**E**, **F**) Transwell assay analyzing the migratory and invasive abilities of HCT8 cells (**E**) and HCT116 cells (**F**) following PPA1 overexpression (100× magnification); (**G**, **H**) Wound healing assay illustrating the effect of PPA1 overexpression on the migratory capacity of HCT8 cells (**G**) and HCT116 cells (**H**) (100× magnification); (**I**, **J**) Western blotting analysis of PPA1, E-cadherin, and N-cadherin expression levels in HCT8 cells (**I**) and HCT116 cells (**J**) after PPA1 overexpression. All data are presented as Mean ± SD, *n* = 3. *p < 0.05, **p < 0.01, ***p < 0.001, ****p < 0.0001.

To functionally validate the tumor-promoting role of PPA1, lentiviral overexpression constructs encoding PPA1 were transduced into HCT8 and HCT116 cells. Similarly, overexpression of PPA1 promoted the proliferation and colony-forming ability of CRC cells, and this promoting effect was more significant under glucose restriction conditions (0.25 mM glucose) (Fig. 3A–D, Fig. S2F, G). Moreover, PPA1 overexpression significantly enhanced the migration and invasion capacities of CRC cells in the OE-PPA1 group compared to the OE-NC group, as demonstrated by Transwell assay and wound healing assay (Fig. 3E–H). Consistently, under glucose-restricted conditions (0.25 mM glucose), overexpression of PPA1 further enhanced the pro-invasive and migratory effects of CRC cells (Fig. S2H–J). These findings suggest that PPA1 may regulate proliferation, migration and invasion of CRC cells through glucose metabolism-related pathways. We further examined the alterations in PPA1 protein levels under glucose-restricted condition and found that the expression of PPA1 was significantly upregulated in this context (Fig. S2K, L). In addition, PPA1 overexpression significantly suppressed E-cadherin expression and enhanced N-cadherin expression, indicative of EMT progression (Fig. 3I, J).

Collectively, the above findings demonstrate that PPA1 functionally drives multiple oncogenic processes in CRC cells, and this oncogenic effect is amplified under glucose-restricted conditions.

### PPA1 promotes the mitochondrial OXPHOS level in CRC cells

While the aforementioned cellular functional assays provide preliminary validation of PPA1’s critical role in CRC, the precise molecular mechanisms remain elusive. Notably, under glucose restriction conditions (0.25 mM glucose), both PPA1 knockdown and overexpression demonstrated more pronounced inhibitory or promotive effects on proliferation, migration, and invasion of CRC, respectively. These glucose-dependent phenotypic modulations strongly suggest that PPA1 regulates biology of CRC through glucose-related metabolism pathways, potentially involving metabolism reprogramming and energy homeostasis control.

**Fig. 4:**
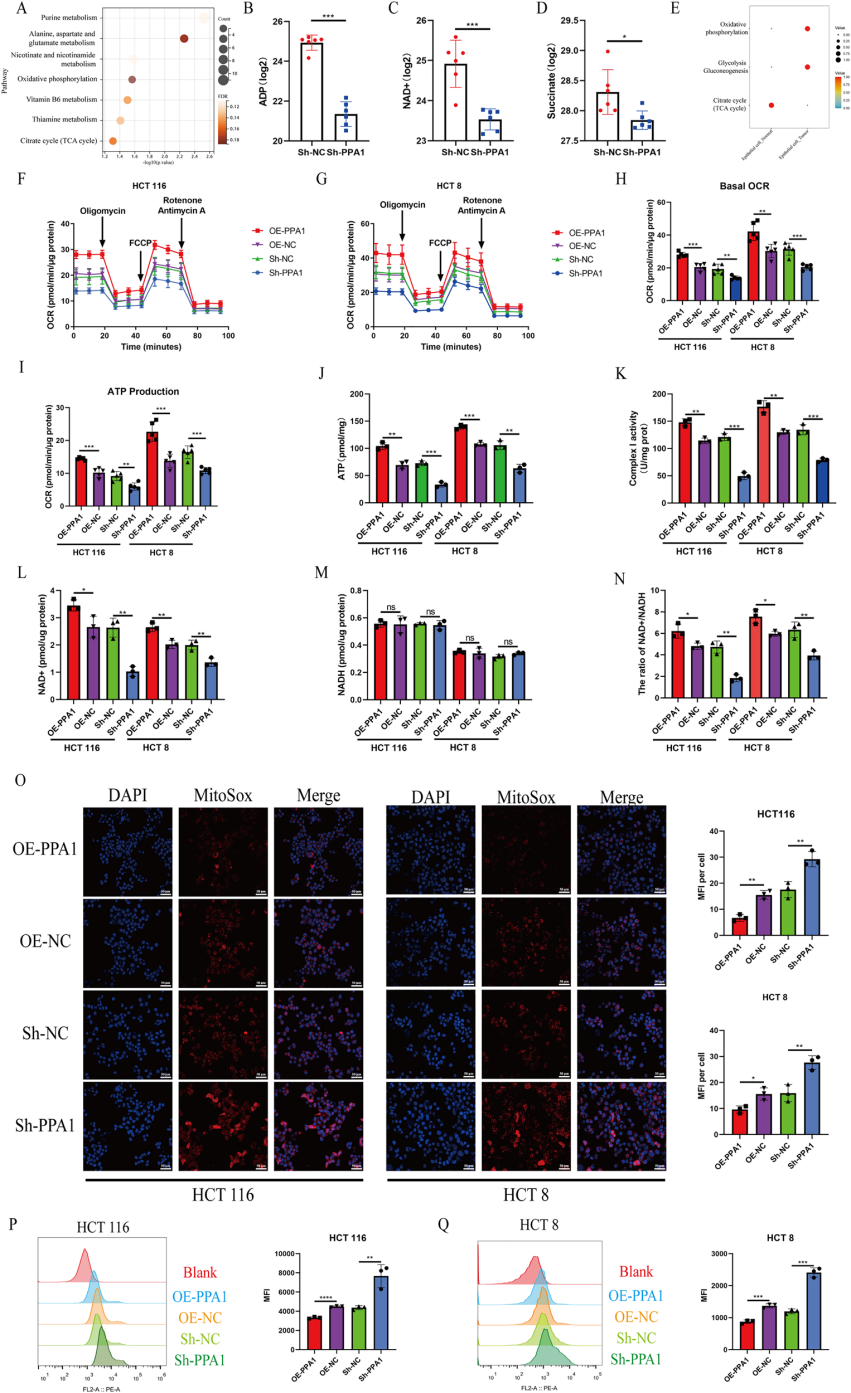
Effects of PPA1 on OXPHOS in CRC cells. Under Glucose-restricted conditions, (**A**) Pathways enriched with differentially expressed metabolites in HCT116 cells after PPA1 knockdown; (**B**–**D**) Effects of PPA1 knockdown on levels of ADP (**B**), NAD+ (**C**), and succinate (**D**) (Mean ± SD, *n* = 6); (**E**) Activity of major energy metabolism pathways in epithelial cells of CRC and adjacent normal tissues from the GSE200997 dataset; (**F**, **G**) Mitochondrial OXPHOS activity in HCT8 (**F**) and HCT116 (**G**) cells measured using the Seahorse XF24 Analyzer. Arrows indicate sequential addition of oligomycin (1 μM), FCCP (1.5 μM), rotenone (0.5 μM), and antimycin A (0.5 μM) after baseline establishment; (**H**, **I**) Quantification of basal OCR(H) and ATP production (**I**) (Mean ± SD, *n* = 5); (**J**, **K**) Measurement of ATP levels (**J**), mitochondrial respiratory chain complex I activity (**K**), NAD+ levels (**L**), NADH levels (**M**) and NAD + /NADH ratio (**N**) in HCT8 and HCT116 cells using a microplate reader (Mean ± SD, *n* = 3); (**O**) Mitochondrial ROS levels in CRC cells assessed by confocal microscopy (Mean ± SD, *n* = 4, 100× magnification); (**P**, **Q**) Flow cytometry analysis of mitochondrial ROS levels in HCT8 (**P**) and HCT116 (**Q**) cells (Mean ± SD, *n* = 3); All data are presented as Mean ± SD. *p < 0.05, **p < 0.01, ***p < 0.001, ****p < 0.0001.

Hence, we performed metabolomic profiling to assess metabolic alterations in HCT116 cells between Sh-PPA1 and Sh-NC groups cultured under glucose restriction conditions for 24 h. The KEGG analysis revealed that differentially altered metabolites were predominantly enriched in pathways including purine metabolism, amino acid metabolism, OXPHOS, and the tricarboxylic acid (TCA) cycle (Fig. 4A). Notably, OXPHOS-associated metabolites—adenosine diphosphate (ADP), nicotinamide adenine dinucleotide (NAD + ), and succinate—exhibited significantly lower levels in the Sh-PPA1 group compared to the Sh-NC group (Fig. 4B–D). Concurrently, we employed the “scMetabolism” R package to calculate metabolic pathway activity in epithelial cells from CRC tissues and adjacent normal tissues within the GSE20997 dataset. The results demonstrated that epithelial cells of CRC tissues exhibited significantly elevated activity in both OXPHOS and glycolytic pathways compared with their normal counterparts (Fig. 4E). These findings align with recent studies identifying a marked OXPHOS propensity in CRC cells to meet their substantial energetic demands, establishing OXPHOS as a crucial energy source in colorectal carcinogenesis [19, 20, 33]. Collectively, we believe that PPA1 may modulate the biological behaviors of CRC cells through the OXPHOS pathway.

Next, we evaluated OXPHOS activity in HCT116 and HCT8 cells after 24 h low-glucose culture using the Seahorse Mito Stress Test. In both cell lines, PPA1 knockdown significantly attenuated OXPHOS activity, with the Sh-PPA1 group showing reduced basal OCR and ATP production compared to the Sh-NC group. Conversely, PPA1 overexpression markedly enhanced OXPHOS activity, as evidenced by elevated OCR and ATP production in the OE-PPA1 group relative to the OE-NC group (Fig. 4F–I). Parallel measurements of intracellular ATP levels, NAD + , NAD + /NADH ratios, and mitochondrial respiratory chain complex I activity under low-glucose conditions revealed consistent patterns. The Sh-PPA1 group exhibited significantly decreased ATP production, diminished NAD + /NADH ratios, and suppressed complex I activity compared to Sh-NC group (Fig. 4J–N), corroborating our metabolomic findings. In contrast, PPA1 overexpression substantially increased these metabolic parameters in both CRC cell lines (Fig. 4J–N). We also found that the alteration in the NAD⁺/NADH ratio was due specifically to changes in NAD⁺ levels, indicating that PPA1 affects only NAD⁺ production without significantly influencing NADH in both CRC cell lines (Fig. 4L–N). Finally, we investigated the impact of PPA1 on mitochondrial ROS using MitoSOX™ Red fluorescence staining. Quantitative analysis revealed significantly higher mitochondrial ROS levels (measured by mean fluorescence intensity, MFI) in Sh-PPA1 group compared to Sh-NC group, whereas PPA1-overexpressing cells showed markedly reduced MFI relative to OE-NC groups (Fig. 4O). FCM analysis confirmed this bidirectional regulation: PPA1 knockdown substantially increased mitochondrial ROS accumulation, while PPA1 overexpression effectively suppressed ROS generation (Fig. 4P, Q).

In conclusion, our findings demonstrate that PPA1 modulates the malignant behaviors of CRC cells through regulation of the OXPHOS pathway.

### PPA1 promotes ULK1/FUNDC1-mediated mitophagy

To elucidate the specific mechanisms by which PPA1 regulates OXPHOS, we conducted integrated proteomic and phosphoproteomic profiling of HCT116 cells (Sh-NC vs. Sh-PPA1 groups) under low-glucose culture conditions. Phosphoproteomic analysis identified 665 differentially phosphorylated sites (Fig. S3A), mapping to 154 distinct proteins. These phosphoproteins revealed predominant enrichment in mitophagy and canonical autophagy pathways (Fig. S3B). Within the mitophagy pathway, ULK1 exhibited significantly reduced phosphorylation at three critical sites—Ser467, Ser556, and Ser638—upon PPA1 knockdown, accompanied by diminished phosphorylation at Ser17 of FUNDC1 (Fig. S3C). ULK1 serves as the master initiator of autophagosome formation and directly coordinates mitophagy through phosphorylation cascades [34, 35]. FUNDC1, a pivotal receptor for ubiquitin-independent mitophagy, resides on the mitochondrial outer membrane where it recruits LC3 via direct binding to initiate mitophagy [36]. Crucially, ULK1-mediated phosphorylation enhances FUNDC1 phosphorylation at Ser17, establishing a hierarchical signaling axis that triggers mitophagy [37]. Our findings position PPA1 as an upstream regulator of this phospho-regulatory network, promoting ULK1/FUNDC1 phosphorylation to activate mitophagy.

**Fig. 5:**
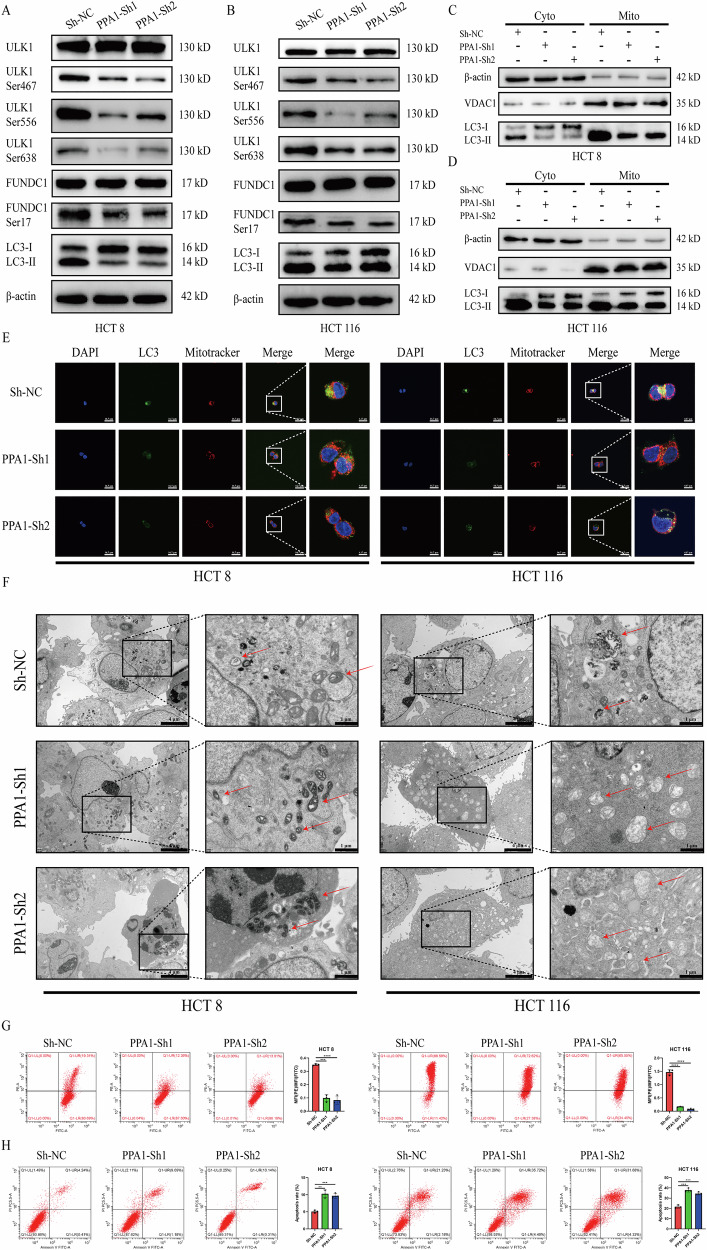
Effects of PPA1 on mitophagy in CRC cells under glucose restriction. Under glucose-restricted conditions, (**A**, **B**) The expression levels of mitophagy-related proteins and phosphorylation sites of HCT8(A) and HCT116(B) cells were detected by Western blotting; (**C**, **D**) Western blotting detecting LC3II and LC3I expression levels in cytoplasmic and mitochondrial fractions of HCT8 (C) and HCT116 (**D**) cells; (**E**) Transmission electron microscopy images showing mitophagy and mitochondria in CRC cells after PPA1 knockdown (red arrows indicate mitophagosomes or mitochondria; left: 8000×, right: 25000× magnification); (**F**) Confocal microscopy imaging of LC3 fluorescence and its co-localization with mitochondria (left: 600×, right: 2400× magnification). **G** Flow cytometry detection of mitochondrial membrane potential in CRC cells (Mean ± SD, *n* = 3). **H** Flow cytometry analysis of apoptosis rates in HCT8 and HCT116 cells (Mean ± SD, *n* = 3). All data are presented as Mean ± SD. **p < 0.01, ***p < 0.001, ****p < 0.0001.

To validate these findings, we conducted a series of functional assays of CRC ells under glucose restriction. As demonstrated in Fig. 5A and Fig. S3D, G, PPA1 knockdown did not significantly alter total protein expression levels of ULK1 or FUNDC1. However, it caused marked reductions in phosphorylation at three critical sites (Ser467, Ser556, and Ser638) of ULK1and the Ser17 site of FUNDC1 in the PPA1-Sh1 and PPA1-Sh2 groups compared to Sh-NC group. Concomitantly, the LC3II/LC3I ratio—a key indicator of autophagic flux—was substantially diminished in both PPA1-Sh1 and PPA1-Sh2 groups relative to the Sh-NC group (Fig. 5A, B, Fig. S3E, H).

To whether mitophagy levels were altered, we analyzed examined changes in LC3II and LC3I expression in mitochondrial and cytosolic fractions of HCT8 and HCT116 cells under glucose restriction. We found that LC3II protein aggregated on mitochondria was significantly reduced in PPA1-Sh1 and PPA1-Sh2 groups compared to the Sh-NC group across both cell lines (Fig. 5C, D, Fig. S3F, I), indicating impaired recruitment of LC3II—a critical step in mitophagy initiation. Confocal microscopy revealed diminished LC3 puncta formation and attenuated colocalization with MitoTracker-labeled mitochondria in PPA1-Sh1 and PPA1-Sh2 groups (Fig. 5E, Fig. S3J–M). Then, we directly observed mitochondrial changes in CRC cells of each group by transmission electron microscopy (TEM). Under low-glucose culture conditions, cells in the Sh-NC group exhibited numerous autophagosomes containing mitochondria and autolysosomes with digested mitochondrial remnants. In contrast, cells in the PPA1-Sh1 and PPA1-Sh2 groups displayed markedly fewer autolysosomes, with the majority of mitochondria showing swelling and structural condensation—morphological hallmarks of mitochondrial dysfunction that may predispose cells to apoptosis (Fig. 5F). Finally, we assessed mitochondrial membrane potential (ΔΨm) across each group using JC-1 fluorescent probe. As shown in Fig. S3N, the Sh-NC group exhibited a significantly higher MFI ratio of JC-1 aggregates (red fluorescence) to JC-1 monomers (green fluorescence) compared with PPA1-Sh1 and PPA1-Sh2 groups. FCM analysis confirmed this pattern, demonstrating that PPA1 knockdown substantially reduced ΔΨm (Fig. 5G). Severe ΔΨm dissipation disrupts mitochondrial functionality, ultimately triggering cell death [38]. We subsequently quantified apoptosis rates via FCM after 24 h glucose restriction. Both CRC cell lines showed significantly elevated apoptosis in PPA1-Sh1 and PPA1-Sh2 groups versus Sh-NC group (Fig. 5H).

In summary, we demonstrate that PPA1 knockdown suppresses ULK1-FUNDC1-mediated mitophagy in CRC cells under low-glucose culture, thereby inducing mitochondrial dysfunction and ultimately promoting apoptosis.

### Phosphorylation of ULK1 at Ser467 and Ser556 sites promotes phosphorylation of FUNDC1 at Ser17 site

**Fig. 6:**
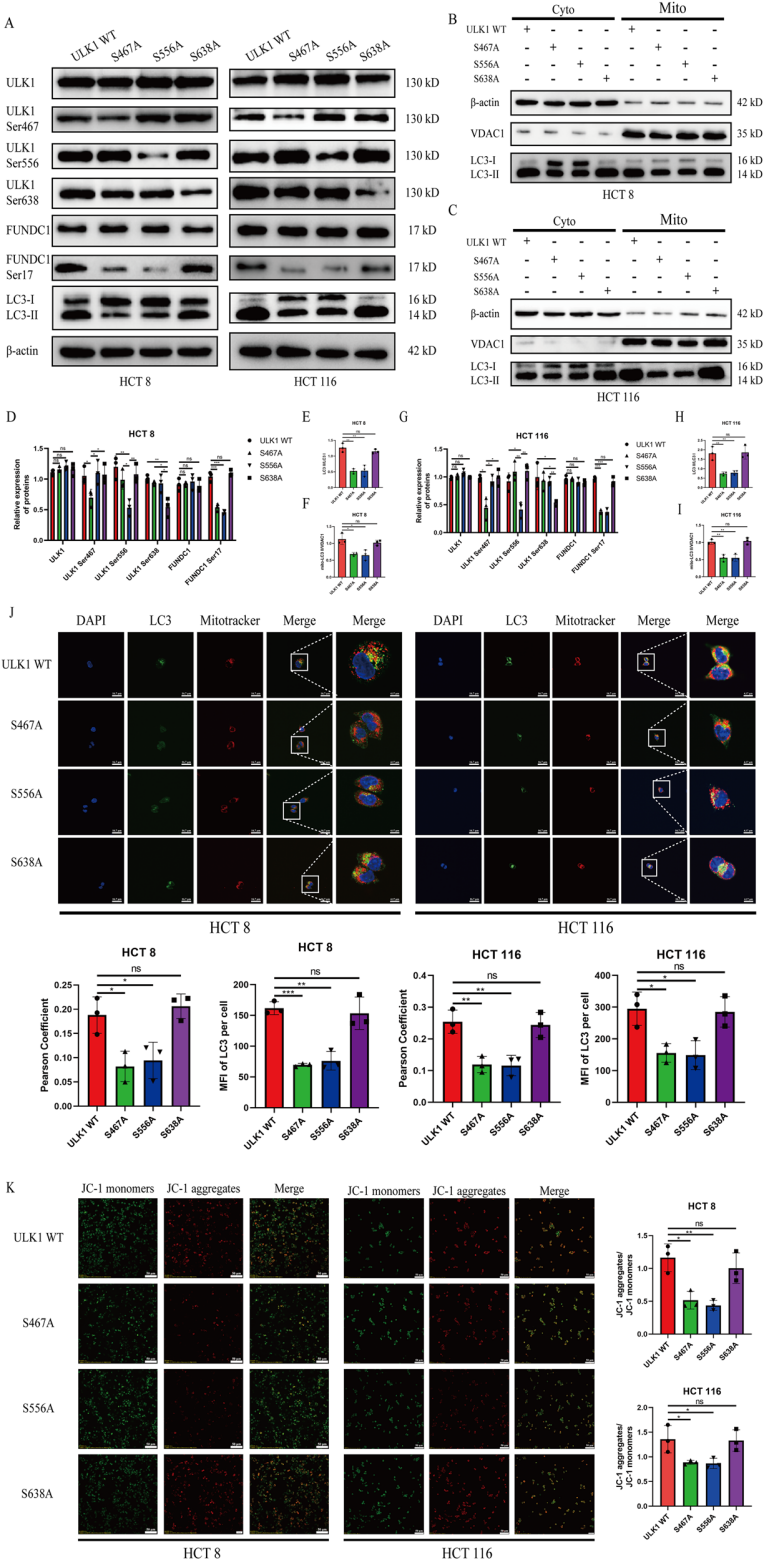
The Ser467 and Ser556 sites of ULK1 regulate FUNDC1-mediated mitophagy. Under glucose-restricted conditions, (**A**) Western blotting analysis of mitophagy-related proteins and phosphorylation levels in CRC cells with ULK1 mutations at Ser467 or Ser556 or Ser638 site; (**B**, **C**) Western blotting detection of LC3II and LC3I expression in cytoplasmic and mitochondrial fractions of HCT8 (B) and HCT116 (**C**) cells after mutating Ser467 or Ser556 or Ser638 site of ULK1; (**D**–**I**) Quantification of protein and phosphorylation site expression levels in HCT8 (**D**−**F**) and HCT116 (**G**−**I**) cells (Mean ± SD, *n* = 3); (**J**) Confocal microscopy imaging and quantitative analysis of LC3 fluorescence and its co-localization with mitochondria (left: 600×, right: 2400 × magnification); (**K**) Confocal microscopy analysis of mitochondrial membrane potential in CRC cells (Mean ± SD, *n* = 3, 200× magnification). All data are presented as Mean ± SD. *p < 0.05, **p < 0.01, ***p < 0.001, ns not significant.

To identify which ULK1 phosphorylation site(s) regulate phosphorylation of FUNDC1 at Ser17 site under glucose restriction, the cells in the Sh-PPA1 group were transfected with either wild-type ULK1 overexpression wild-type plasmid (ULK1 WT group) or phosphorylation-deficient mutants targeting specific residues (S467A or S556 A, or S638A group). As shown in Fig. 6A, D, and G, phosphorylation levels at Ser467 or Ser556 or Ser638 site of ULK1 were significantly reduced in each mutant group compared to the ULK1 WT group. Furthermore, we observed that the phosphorylation level of FUNDC1 at the Ser17 site was markedly decreased in S467A and S556 A groups relative to the ULK1 WT group, whereas no significant change was detected in the S638A group (Fig. 6A, D, G). Similarly, the LC3II/LC3I ratio was substantially reduced in S467A and S556A groups compared to ULK1 WT, while the S638A group showed no alteration (Fig. 6A, E, H). In addition, we observed significantly diminished LC3II enrichment on mitochondria in both S467A and S556A groups compared to the ULK1 WT group, whereas LC3II accumulation in the S638A group remained unaltered. (Fig. 6B, C, F, I). Immunofluorescence analysis revealed significantly reduced LC3 puncta accumulation and diminished colocalization with mitochondria in S467A and S556A mutants compared to ULK1 WT and S638A groups (Fig. 6J). Consistently, JC-1 probe analysis demonstrated a substantially decreased MFI ratio of JC-1 aggregates (red fluorescence) to JC-1 monomers (green fluorescence) in S467A and S556A mutants versus ULK1 WT and S638A groups (Fig. 6K), indicating pronounced ΔΨm dissipation.

In conclusion, we demonstrate that under glucose restriction, phosphorylation of ULK1 at Ser467 and Ser556 sites specifically induces phosphorylation of FUNDC1 at Ser17 site, thereby promoting mitophagy in CRC cells.

### ULK1 agonist LYN-1604 promotes FUNDC1-mediated mitophagy and malignant behavior in CRC cells

Next, we further investigated whether pharmacological activation of ULK1 using its agonist LYN-1604 could directly induce mitophagy of CRC cells under glucose restriction.

**Fig. 7:**
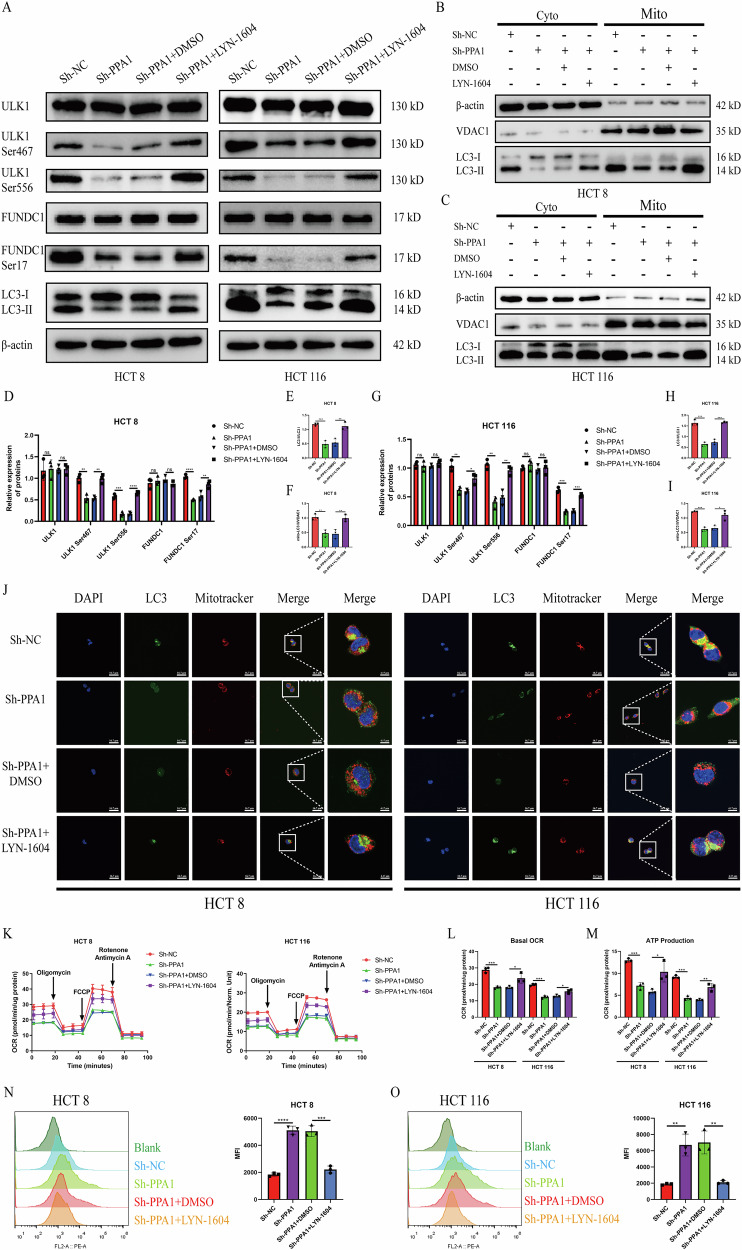
Effects of the ULK1 agonist LYN-1604 on FUNDC1-mediated mitophagy. Under glucose-restricted conditions, (**A**) Western blotting analysis of mitophagy-related proteins and phosphorylation levels in CRC cells treated with the ULK1 agonist LYN-1604 (1 μM); (**B**, **C**) Western blotting detection of LC3II and LC3I expression in cytoplasmic and mitochondrial fractions of HCT8 (**B**) and HCT116 (**C**) cells treated with LYN-1604 (1 μM); (**D**–**I**) Quantification of protein and phosphorylation site expression levels in HCT8 (D-F) and HCT116 (**G**−**I**) cells (Mean ± SD, n = 3); (**J**) Confocal microscopy imaging of LC3 fluorescence and its co-localization with mitochondria in cells treated with or without LYN-1604 (1 μM) (left: 600×, right: 2400 × magnification); (**K**) Impact of LYN-1604 (1 μM) on mitochondrial OXPHOS activity in CRC cells(oligomycin (1 μM), FCCP (1.5 μM), rotenone (0.5 μM), and antimycin A (0.5 μM)); (**L**, **M**) Quantification of basal OCR(L) and ATP production(M) (Mean ± SD, *n* = 3); (**N**, **O**) Flow cytometry analysis of mitochondrial ROS levels in HCT8 (N) and HCT116 (**O**) cells treated with LYN-1604 (Mean ± SD, *n* = 3). All data are presented as Mean ± SD. *p < 0.05, **p < 0.01, ***p < 0.001, ****p < 0.0001.

As shown in Fig. 7A, D, and G, phosphorylation levels of ULK1 at Ser467 and Ser556 sites were significantly elevated in both HCT8 and HCT116 cells treated with Sh-PPA1 and LYN-1604(1 μM) compared to the Sh-PPA1 + DMSO group under glucose restriction. Similarly, the phosphorylation level of FUNDC1 at Ser17 site and the LC3II/LC3I ratio were markedly increased in Sh-PPA1 + LYN-1604 group (Fig. 7A, E, H). Mitochondrial fraction analysis revealed significantly enhanced mitochondrial recruitment of LC3II in the Sh-PPA1 + LYN-1604 group compared to the Sh-PPA1 + DMSO group (Fig. 7B, C, F, I). Confocal microscopy further demonstrated enhanced LC3 puncta formation and increased colocalization with MitoTracker-labeled mitochondria in Sh-PPA1 + LYN-1604 group (Fig. 7J, Fig. S4A–D). These results collectively validate that LYN-1604 effectively rescues PPA1 knockdown-induced deficits in ULK1 phosphorylation and mitophagy.

We further investigated whether ULK1/FUNDC1-mediated mitophagy exerts protective effects on OXPHOS functionality. Seahorse Mito Stress assays demonstrated that Sh-PPA1 + LYN-1604 group in both CRC cell lines exhibited significantly higher OXPHOS activity compared to Sh-PPA1 + DMSO group, with marked increases in basal OCR and ATP production (Fig. 7K–M). FCM analysis further revealed substantially reduced ROS accumulation in Sh-PPA1 + LYN-1604 group versus the Sh-PPA1 + DMSO group (Fig. 7N, O).

Finally, the effects of ULK1 agonist LYN-1604 on the malignant behaviors of CRC cells under glucose restriction were assessed by a series of functional assays. CCK-8 assay demonstrated significantly increased proliferative capacity in Sh-PPA1 + LYN-1604 group compared to Sh-PPA1 + DMSO and Sh-PPA1 groups in both HCT8 and HCT116 cells (Fig. S4E). Colony formation assay revealed a higher number of colonies in the Sh-PPA1 + LYN-1604 group than in Sh-PPA1 + DMSO and Sh-PPA1 groups (Fig. S4F). Wound healing assays showed greater migration distances and rates in Sh-PPA1 + LYN-1604-treated CRC cells compared to controls (Fig. S4G, H). Transwell assays further confirmed increased numbers of migrated and invaded cells in the Sh-PPA1 + LYN-1604 group relative to Sh-PPA1 + DMSO and Sh-PPA1 groups (Fig. S4I, J).

Collectively, these findings demonstrate that under glucose restriction, LYN-1604-activated ULK1 effectively promotes mitophagy, preserves OXPHOS functionality, and consequently drives malignant progression of CRC cells.

### PPA1 promotes phosphorylation of ULK1 through AMPK

**Fig. 8:**
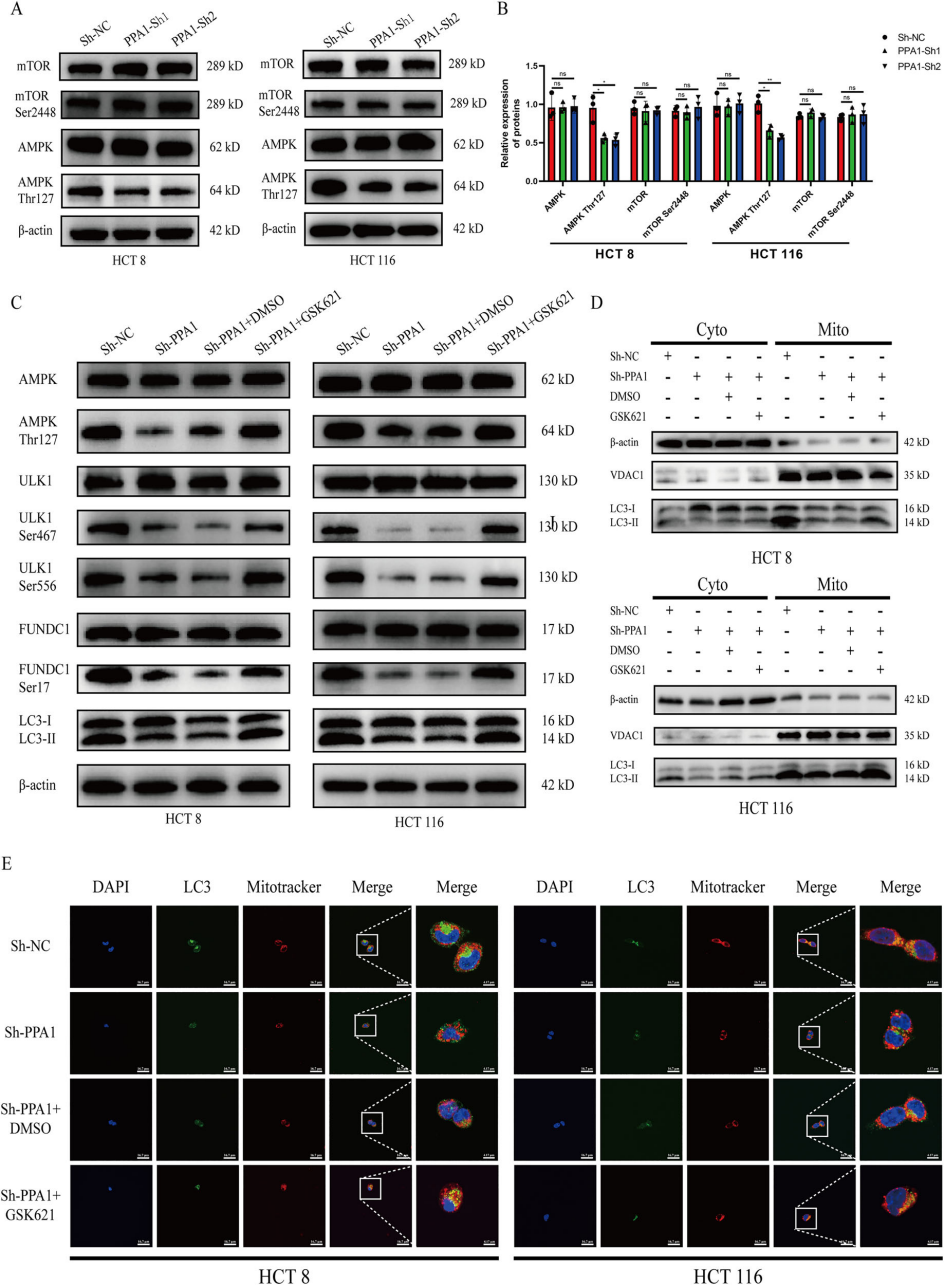
Effects of the AMPK agonist GSK621 on ULK1/FUNDC1-mediated mitophagy. Under glucose-restricted conditions, (**A**, **B**) The protein levels and phosphorylation status of both mTOR and AMPK after PPA1 knockdown; (**C**, **D**) The mitophagy-related proteins and phosphorylation levels in CRC cells treated with or without the AMPK agonist GSK621 (30 μM); (**E**) Confocal microscopy imaging of LC3 fluorescence and its co-localization with mitochondria in cells treated with or without GSK621 (30 μM) (left: 600×, right: 2400 × magnification). All data are presented as Mean ± SD. *p < 0.05, **p < 0.01, ns not significant.

AMPK and mTOR represent classical upstream pathways regulating ULK1 phosphorylation [39]. To further investigate whether PPA1-mediated regulation of ULK1 depends on AMPK or mTOR signaling, we examined both protein expression and phosphorylation levels of mTOR and AMPK. As shown in Fig. 8A, B, PPA1 knockdown did not alter AMPK protein expression, but significantly reduced its phosphorylation at Thr172 site—a critical site governing ULK1 phosphorylation. In contrast, neither protein level nor phosphorylation status of MTOR was affected (Fig. 8A). These results suggest that PPA1 modulates ULK1 phosphorylation potentially through regulating AMPK phosphorylation rather than via the MTOR pathway.

Next, we employed the AMPK agonist GSK621 to investigate whether AMPK activation could rescue the downstream effects induced by PPA1 knockdown. As demonstrated, GSK621 (30 μM) treatment markedly enhanced phosphorylation of AMPK at Thr172 site in both cell lines (Figs. 8C, S5A, B). Subsequently, phosphorylation levels at Ser467 and Ser555 sites of ULK1 and Ser17 site of FUNDC1 were correspondingly elevated (Fig. 8C, Fig. S5A, B). As expected, total autophagy (LC3II/LC3 I ratio) and mitophagy levels (LC3II level in mitochondrion) were significantly increased accordingly (Fig. 8C, D, Fig. S5C–F). Finally, we similarly observed that the Sh-PPA1 + GSK621 group exhibited increased LC3 puncta formation and enhanced co-localization with MitoTracker-labeled mitochondria compared to the Sh-PPA1 + DMSO group (Fig. 8E, Fig. S5G–J).

Finally, we also investigated the impact of the AMPK agonist GSK621 on the malignant behavior of CRC cells. As shown in Fig. S6A, B and C, the cells in Sh-PPA1 + GSK621 group exhibited significantly enhanced proliferation and colony formation capabilities compared to the Sh-PPA1 + DMSO group. Transwell assays further indicated a marked increase in the number of invading and migrating cells in the Sh-PPA1 + GSK621 group relative to Sh-PPA1 + DMSO group (Fig. S6D, E). Similarly, wound healing assays demonstrated that GSK621 treatment significantly promoted the migratory capacity of both CRC cell lines (Fig. S6F, G).

Collectively, these results indicate that PPA1 modulates ULK1-dependent mitophagy and enhances the malignant behaviors—including proliferation, migration, and invasion—in CRC cells via AMPK.

### PPA1 promotes the growth and hepatic metastasis of CRC cells in vivo

To further validate the impact of PPA1 on CRC biology in vivo, we established subcutaneous xenograft and hepatic metastasis models in nude mice.

**Fig. 9:**
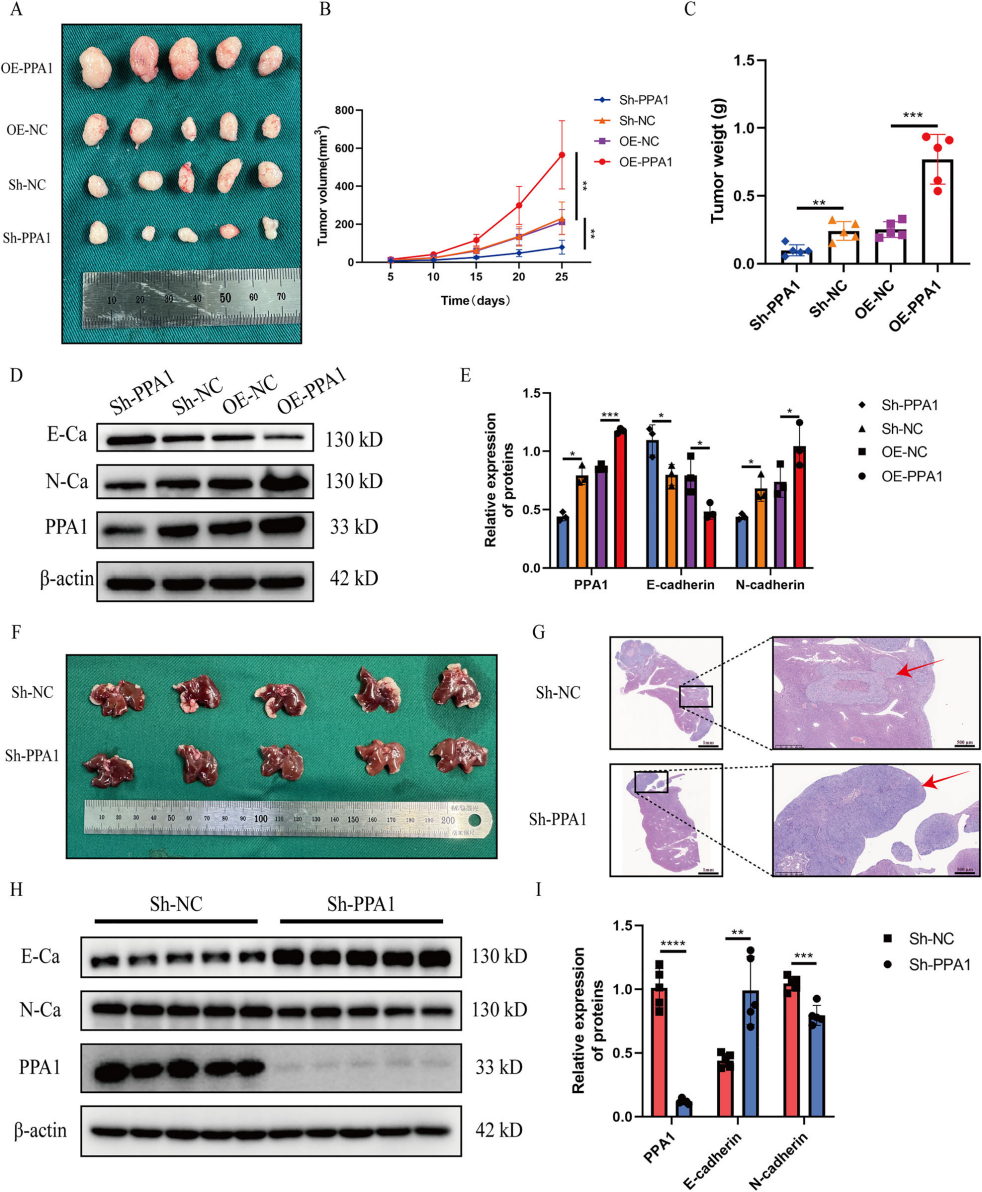
Effects of PPA1 on subcutaneous tumorigenesis and liver metastasis of CRC cells in nude mice. **A** Impact of PPA1 knockdown or overexpression on subcutaneous xenograft tumors; (**B**) Tumor volume growth curves of subcutaneous xenografts in each group; (**C**) Tumor weight of subcutaneous xenografts in each group; (**D**, **E**) Expression levels and statistical analysis of PPA1, E-cadherin, and N-cadherin in subcutaneous tumors across groups; (**F**) Effect of PPA1 knockdown on liver metastasis of CRC; (**G**) HE staining showing CRC metastatic tissues in the liver of nude mice (red arrows: CRC tissues; left: 10×, right: 100 × magnification); (**H**, **I**) Expression levels and statistical analysis of E-cadherin and N-cadherin in liver metastatic CRC tissues following PPA1 knockdown. All data are presented as Mean ± SD, *n* = 5. *p < 0.05, **p < 0.01, ***p < 0.001, ****p < 0.0001.

Stably transfected HCT116 cells (Sh-PPA1, Sh-NC, OE-NC, and OE-PPA1 groups) were subcutaneously injected into nude mice. Tumor volumes were measured on days 5, 10, 15, 20, and 25. Growth curve analysis revealed that PPA1 knockdown significantly inhibited subcutaneous tumor growth, with the Sh-PPA1 group exhibiting smaller tumor volumes and lower weights compared to the Sh-NC group. Conversely, PPA1 overexpression markedly promoted tumor growth, as evidenced by larger tumor volumes and higher weights in the OE-PPA1 group versus the OE-NC group (Fig. 9A–C). Western Blotting analysis of tumor tissues revealed elevated E-cadherin and decreased N-cadherin expression in the Sh-PPA1 group compared to the Sh-NC group, whereas the OE-PPA1 group exhibited the opposite trend compared to the OE-NC group (Fig. 9D, E). A CRC liver metastasis model was established via splenic injection of stably transfected HCT116 cells (Sh-PPA1 and Sh-NC groups). Macroscopic examination showed fewer metastatic CRC lesions in the livers of the Sh-PPA1 group compared to the Sh-NC group (Fig. 9F). H&E staining further confirmed a reduced number of metastatic foci in the Sh-PPA1 group (Fig. 9G). As demonstrated in Fig. 9H, I, following the knockdown of PPA1, E-cadherin expression was upregulated markedly, whereas N-cadherin expression was downregulated significantly in CRC metastatic foci. The above results confirm that PPA1 can also promote the growth and metastasis of CRC cells in vivo.

**Fig. 10:**
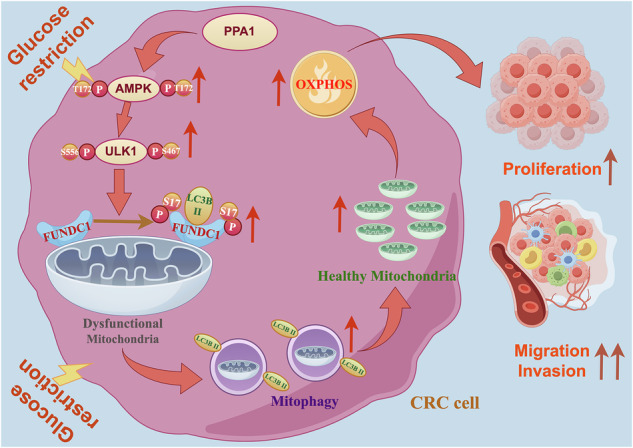
The specific mechanism by which PPA1 affects the proliferation, migration and invasion of CRC cells under glucose restriction.

In summary, PPA1 serves as a critical cytoprotective protein in CRC cells under glucose restriction. Under such metabolic stress, PPA1 promotes phosphorylation of AMPK at Thr172 site and thereby facilitates phosphorylation of ULK1 at Ser467 and Ser555 sites. These phosphorylation events specifically induce phosphorylation of Ser17 on FUNDC1, a mitophagy receptor localized at the mitochondrial outer membrane. Phosphorylated FUNDC1-Ser17 recruits LC3II proteins to initiate mitophagy. This selective mitochondrial clearance mechanism maintains intracellular homeostasis and mitochondrial pool stability, thereby preserving OXPHOS functionality to generate sufficient energy for driving CRC malignant behaviors—including proliferation, migration, and invasion (Fig. 10).

## Discussion

Despite the continued improvement in CRC mortality with the application of chemotherapy and targeted therapies, the development of novel treatment strategies remains urgently needed due to challenges such as chemotherapy resistance and the rising incidence of early-onset CRC [40, 41]. Metabolic reprogramming plays a critical role in CRC pathogenesis, enabling cancer cells to acquire metabolic traits that support survival, immune evasion, and proliferative growth [42]. In some cases, specific metabolic vulnerabilities of cancer cells have been successfully translated into effective therapies for cancers. Understanding and modulating the metabolic landscape of CRC represents a promising direction for targeted therapies [43, 44]. Through ScRNA-Seq data analysis, we identified that PPA1, a gene associated with OXPHOS, is highly expressed in epithelial cells of CRC. Accumulating clinical evidence reveals that CRC is not solely a glycolytic tumor, but rather demonstrates increased expression of OXPHOS-related enzymes that drive invasion and metastasis in advanced stages, particularly in microsatellite-stable and KRAS-mutant subtypes [45, 46]. The OXPHOS system serves as a primary ATP producer in CRC cells, and its inhibition has been shown to suppress tumor proliferation and metastasis [47]. Accordingly, therapeutic targeting of OXPHOS with specific inhibitors represents a promising strategy to effectively suppress CRC progression and metastatic dissemination [48]. We found that elevated PPA1 expression correlates with larger tumor size, lymph node status, advanced TNM stage, and poor patient prognosis. Knockdown of PPA1 significantly suppressed the malignant behaviors of CRC cells, including proliferation, migration, and invasion, both in vitro and in vivo. Mechanistically, this effect is attributed to the potent inhibition of OXPHOS activity in CRC cells upon PPA1 depletion.

ULK1, a serine-threonine kinase encoded at chromosome 12q24.3 and the mammalian ortholog of yeast Atg1 [49], regulates mitophagy through interactions with FUNDC1, BNIP3, and NIX receptors [50–52]. ULK1 assembles into a stable multimeric complex with ATG13, RB1CC1/FIP200, and ATG101, which is essential for initiating canonical autophagy [53]. Additionally, ULK1 directly phosphorylates mitophagy receptors including Nip3-like protein X (NIX), BCL2-interacting protein 3-like (BNIP3L), BCL2-interacting protein 3 (BNIP3), and FUNDC1, thereby activating mitophagy [37, 51, 54]. ULK1 activity is predominantly governed by dynamic phosphoregulation networks. Canonical studies have established its dual regulatory paradigm involving AMPK-mediated activation and mTORC1-dependent suppression, which operate reciprocally in response to cellular nutrient-sensing mechanisms [55]. Our study identifies PPA1 as a critical regulator of ULK1 phospho-activation (467/556/638 sites) in CRC cells under nutrient stress imposed by glucose limitation. Whether the PPA1-ULK1 regulatory axis operates independently of the canonical AMPK signaling pathway remains to be mechanistically elucidated.

Mitophagy can be categorized into ubiquitin-dependent and ubiquitin-independent pathways [56]. Ubiquitin-dependent mitophagy is primarily mediated by the PINK1-Parkin pathway and other autophagy receptors such as OPTN and NDP52 [57–62]. In contrast to ubiquitin-dependent pathways, mitophagy independent of ubiquitination utilizes OMM-localized receptors (e.g., FUNDC1, BNIP3, NIX, BCL2L13) that engage LC3 via direct binding, bypassing the requirement for ubiquitin tagging [63–69].

FUNDC1 activity is tightly regulated by post-translational modifications. Under normoxia, phosphorylation of FUNDC1 at Tyr18 and Ser13 within its LC3-interacting region (LIR) by Src and CK2 kinases, respectively, blocks its interaction with LC3; Prolonged hypoxia inactivates Src and CK2 kinases, leading to FUNDC1 dephosphorylation at these sites and subsequent mitophagy activation [70]. Hypoxia also induces BCL2L1 degradation, releasing PGAM5 to dephosphorylate FUNDC1-Ser13 and enhance LC3 binding [71]. Under hypoxia, ULK1 translocates to fragmented mitochondria and phosphorylates FUNDC1 at Ser17, promoting its interaction with LC3B to initiate mitophagy [52, 72]. However, the specific ULK1 phosphorylation sites responsible for FUNDC1 activation remain undefined. Our study reveals that under glucose restriction, phosphorylation of ULK1 at Ser467 and Ser556 specifically triggers FUNDC1-Ser17 phosphorylation in CRC cells, driving FUNDC1-dependent mitophagy.

We demonstrate that FUNDC1-mediated mitophagy is not only hypoxia-inducible but also essential for maintaining CRC cell homeostasis under glucose deprivation. Mechanistically, glucose restriction activates the phosphorylation of AMPK at Thr 172 site and ULK1 at Ser467/Ser556 sites, which in turn phosphorylates FUNDC1-Ser17 to initiate mitophagy. PPA1 knockdown disrupts this axis, suppressing phosphorylation of AMPK and ULK1 activation, impairing mitophagy, and causing mitochondrial dysfunction (OXPHOS collapse, ROS accumulation). These metabolic disruptions ultimately inhibit CRC proliferation, migration, and invasion. Our findings establish PPA1 as a critical regulator of the AMPK/ULK1/FUNDC1-mitophagy-OXPHOS axis, enabling CRC adaptation to nutrient stress and highlighting its therapeutic potential.

## Conclusions

This study identifies PPA1 as a novel metabolic dependency in CRC, offering a strategic target for disrupting tumor survival under glucose-limited conditions characteristic of the tumor microenvironment.

## Materials and methods

### Data collection

Single-cell RNA-sequencing (ScRNA-seq) data on 16 CRC samples and 7 adjacent normal tissues were obtained from the GSE200997 datasets in the Gene Expression Omnibus (GEO) database (https://www.ncbi.nlm.nih.gov/geo/). RNA-seq data from 288 CRC patients were obtained from The Cancer Genome Atlas (TCGA) database (https://portal.gdc.cancer.gov/), while RNA-seq data from 308 normal colon tissues were retrieved from the Genotype-Tissue Expression (GTEx) database (https://www.gtexportal.org/home/).

### ScRNA-seq data analysis

Following the standard single-cell RNA sequencing (scRNA-seq) analysis workflow, a merged Seurat object was generated, integrating data from 16 CRC tissues and 7 matched adjacent normal tissues [73]. To obtain high-quality scRNA-seq data, cells with <100 measured genes, cells with >25% mitochondrial contamination, and cells with >4000 measured genes were removed, with the remaining 48,681 high-quality cells selected for subsequent analysis. Then, the merged object was normalized via the “NormalizeData” function, and the batch effect of the 23 samples was corrected using the “Harmony” package. Twenty harmony dimensions were evaluated, and the top two uniform manifold approximation and projection (UMAP) dimensions were visualized at a clustering resolution of 0.5. The 18 cell clusters were annotated as 9 cell clusters each based on previously described cell markers [31] and on CellMarker 2.0 (http://bio-bigdata.hrbmu.edu.cn/CellMarker/). The cell markers included B cells (CD79A, CD19), plasma cells (CD79A, MZB1, IGHA1), T cells (CD3D, CD3E, IL7R), NK cells (GNLY, KLRD1, NKG7), endothelial cells (PECAM1, VWF, CD34), epithelial cells (EPCAM, KRT8), fibroblasts (ACTA2, COL1A1, COL1A2), myeloid cells (CD14, LYZ, CD68, CD163), mast cells (KIT, MS4A2). The differentially expressed genes (DEGs) with |log2FC | > 0.58 and *P*-value < 0.05 of epithelial cells between CRC and adjacent tissues were identified using the “FindAllMarkers” function of the “Seurat” package.

### Clinical samples collection

A total of 48 paired CRC and adjacent normal tissue samples were collected from the Third Affiliated Hospital of Chongqing Medical University between October 2022 and March 2023 for subsequent experiments. The protocols used in this research were evaluated and approved by the Ethics Committee of the Third Affiliated Hospital of Chongqing Medical University. Written informed consent was obtained from all subjects. All methods of this study were carried out in accordance with the Declaration of Helsinki.

### qRT-PCR

The detailed methodologies have been comprehensively described in our prior study [74]. All samples were normalized according to β-actin expression. The specific primer sequences for each gene are listed below: β-actin Forward: 5’-CTACCTCATGAAGAT CCTGACC-3’; Reverse: 5’-CACAGCTTCTCTTTGATGTC AC-3’; PPA1 Forward: 5’-TTGCGAATTTGTTCCCGTATAAAGG-3’; Reverse: 5’-TGTCACCACAACAGC CAGTATG-3’. The relative expression of PPA1 across all tissue samples was quantified using the 2^(-ΔΔCt) method, followed by statistical analysis.

### Western blotting

The detailed methodologies have been comprehensively described in our prior study [74]. All primary antibodies were commercially sourced as follows: PPA1 (ab181126, 1:10,000), LC3B (ab192890, 1:2,000), and ULK1 phosphorylated at Ser638 (ab179458, 1:1,000) from Abcam; mTOR(66888-1-Ig, 1:5,000), mTOR phosphorylated at Ser2448(80596-1-RR, 1:5,000), β-actin (66009-1-Ig, 1:5,000) and GAPDH (60004-1-Ig, 1:5,000) from Proteintech; AMPK(A27740, 1:5,000), AMPK phosphorylated at Thr172(AP0116, 1:1,000), and ULK1 phosphorylated at Ser467 (AP1262, 1:1,000) and Ser556 (AP1495, 1:1,000) from ABclonal Technology; FUNDC1 (AF0002, 1:1,000) and FUNDC1 phosphorylated at Ser17 (AF0001, 1:1,000) from Affinity Biosciences; N-Cadherin (#13116, 1:1,000) and E-Cadherin (#3195, 1:1,000) from Cell Signaling Technology; and ULK1 (R381887, 1:1,000) from ZenBio. The incubated PVDF membranes were thoroughly coated with ECL working solution, developed using a Bio-Rad ChemiDoc Imaging System, and subsequently analyzed with ImageJ software for quantitative assessment.

### Cell culture

The CRC cell lines (HCT116, HCT8, HT29, SW480, SW620) and normal intestinal epithelial cell line NCM460 were purchased from the American Type Culture Collection (ATCC). All cell lines were identified by STR and tested for mycoplasma contamination. All cells were cultured in DMEM medium (Gibco, USA) supplemented with 10% fetal bovine serum (FBS, BI, Israel) and 1% penicillin-streptomycin (Gibco, USA) at 37 °C in a 5% CO₂ incubator. For glucose-restricted conditions, HCT8 and HCT116 cells were cultured in medium containing 10% FBS, 1% penicillin-streptomycin, and 0.25 mM glucose under the same temperature and CO₂ conditions.

### Lentivirus and plasmid transfection

Lentivirus-mediated short hairpin RNA (shRNA) targeting PPA1 and the PPA1-overexpressing lentivirus were purchased from Hanbio Biotechnology, with detailed sequences provided in Table S1. Cells were cultured to 70-80% confluence and transduced with the respective lentiviral supernatants at a multiplicity of infection (MOI) of 15, supplemented with 5 μg/mL polybrene as a transduction enhancer. At 72 h post-transduction, stable transfected cell lines were selected using puromycin (10 μg/mL for HCT8 cells, 2 μg/mL for HCT116 cells) for 1–2 weeks.

The ULK1 overexpression plasmids and phosphorylation site mutant plasmids (S467A, S556A, and S638A plasmids) were obtained from Sangon Biotech (Shanghai, China), with detailed sequences provided in Table S2. According to the manufacturer’s instructions (Invitrogen, Carlsbad, CA, USA), plasmids were transfected into HCT8 and HCT116 cell lines using Lipofectamine 3000 reagent.

### Immunohistochemical staining

Paraffin-embedded tissue sections were deparaffinized by incubation in a 60 °C oven to melt the wax, followed by two 10 min immersions in xylene for dewaxing. Sections were rehydrated through a graded ethanol series (100%, 95%, 80%, 75%, 60%) and treated with 3% hydrogen peroxide (H₂O₂) at room temperature for 10 min to quench endogenous peroxidase activity. Antigen retrieval was performed by immersing sections in boiling EDTA antigen retrieval buffer for 10 min, followed by natural cooling. Non-specific binding was blocked with 5% bovine serum albumin (BSA) at 37 °C for 60 min. Sections were incubated with primary antibody against PPA1 (1:1000, 14985-1-AP) overnight at 4 °C. The next day, slides were washed three times with phosphate-buffered saline (PBS; 10 min per wash) and incubated with a species-matched secondary antibody at room temperature for 60 min. After three additional PBS washes, staining was developed using 3,3′-diaminobenzidine (DAB) substrate, counterstained with hematoxylin, and dehydrated through an ascending ethanol series (60%, 75%, 80%, 95%, 100%). Finally, sections were mounted with neutral balsam and imaged under a light microscope.

### Cell counting Kit-8

The 2 × 10^3^ CRC cells were seeded into 96-well plates and cultured with complete medium at 37 °C in 5% CO_2_. Then, we assessed the cell viability of CRC cells on days 0, 1, 2, 3, and 4 after being cocultured with CCK-8 solution at 37 °C in 5% CO_2_ for 2 h. The absorbance at 450 nm was measured via a microplate reader. The CCK-8 kit was obtained from GlpBio Company Ltd. (Shanghai, China).

### Colony formation assays

The 2 × 10^3^ CRC cells were seeded into 6-well plates and cultured with complete medium in 5% CO_2_ for 10 days. After colony formation, the colonies were fixed with 4% paraformaldehyde, stained with 0.1% crystal violet, and subsequently observed and photographed under a microscope.

### Transwell assay

The transwell assays were performed in Boyden chambers and placed in 24-well cell culture plates, with pore sizes of 8 μm in polycarbonate membranes. Boyden chambers with a pre-coated layer of basement membrane matrix were used for the invasion assay, and those without a coated matrix were used for the migration assay. After transfection for 48 h, 1 × 10^5^ CRC cells were added to the upper chamber with 200 μL serum-free medium, and 500 μL 20% FBS-containing medium used as the chemical attractant was added to the lower chamber. After incubation for 48 h, the remaining cells in the upper chamber were removed using the cotton swab. Paraformaldehyde (4%) was used to fix the cells in the bottom membrane, and the 0.1% crystal violet was used for cell staining. The fields were randomly selected on an inverted light microscope (100×), and the cell numbers were counted using ImageJ software.

### Wound healing assay

Transfected CRC cells were seeded into 6-well plates and cultured to ≥ 90% confluence. A sterile pipette tip was used to create a uniform scratch in the cell monolayer. After washing with PBS to remove detached cells, the cells were incubated in high-glucose medium (2.5 mM glucose) or low-glucose medium (0.25 mM glucose) (both supplemented with 2% FBS) for 48 h. Wound closure was assessed by capturing brightfield images at 0 h and 48 h post-scratch, and cell migration was quantified using ImageJ software.

### Animals and protocols

Female BALB/c nude mice (4−6 weeks, weighing 18 ± 2 g) were purchased from Jiangsu GemPharmatech Co., Ltd. All animal procedures were reviewed and approved by the Institutional Animal Care and Use Committee (IACUC) of Chongqing Medical University (Approval No. IACUC-CQMU-2024-0259). All mice were ear-notched for identification and then randomly assigned to experimental groups through computer-generated randomization of ear notch codes, with five mice per group (*n* = 5). All experimental data collection and analysis were recorded and tracked using the unique ear-notch codes assigned to each mouse. For subcutaneous xenograft model, 1 × 10⁷ CRC cells were subcutaneously injected into the right flank of nude mice. Tumor volumes were measured every 5 days (days 5, 10, 15, 20, and 25 post-inoculation) using the formula: *V* = (width^2^ × length)/2. Mice were euthanized on day 25, and tumors were excised, weighed, and photographed for volumetric analysis. To establish hepatic metastasis model, 3 × 10⁶ CRC cells were intrasplenically injected into nude mice. After 15 days, mice were euthanized, and liver tissues were harvested. Tissue sections were stained with H&E for histopathological examination of metastatic foci under a light microscope.

### Histopathological examination

The paraffin sections were placed in a 60 °C oven to melt the paraffin and soaked in xylene and different concentrations of ethanol to elute the paraffin. Next, the sections were stained with hematoxylin for 5 min, differentiated by incubation with 5% acetic acid for 1 min, and then washed separately with running water. After staining with eosin and washing with running water, the sections were sequentially immersed in 60%, 75%, 80%, 95%, and 100% ethanol for dehydration. Finally, the sections were sealed with neutral gum and observed with a light microscope.

### Mitochondrial stress test

Mitochondrial oxygen consumption rate (OCR) of each experimental group was measured using the XF24 Seahorse Extracellular Flux Analyzer (Agilent Seahorse Bioscience, USA). Cells (20,000 per well) were seeded into cell culture microplates and cultured overnight. Simultaneously, the sensor cartridge was hydrated with calibration solution in a non-CO₂ incubator at 37 °C overnight. One hour prior to measurement, the growth medium in the microplate was replaced with 500 μL XF Assay Medium (DMEM supplemented with 10 mM glucose, 2 mM glutamine, and 1 mM sodium pyruvate, pH 7.4), and the microplate was equilibrated in a 37 °C non-CO₂ incubator. Oligomycin (1 μM), FCCP(1.5 μM), and rotenone/antimycin A (0.5 μM) were loaded into ports A, B, and C of the sensor cartridge, respectively. The mitochondrial stress test was performed using the standard Seahorse XF Cell Mito Stress Test protocol.

### Intracellular ATP assay

Intracellular ATP levels in CRC cells across experimental groups were quantified using an ATP Assay Kit (Beyotime, China) following the manufacturer’s protocol. Relative luminescence units (RLUs) were measured with a multimode microplate reader (Thermo fisher scientific, USA).

### Mitochondrial respiratory chain complex I activity assay

Mitochondrial respiratory chain complex I activity in CRC cells across experimental groups was measured using a Mitochondrial Respiratory Chain Complex I Activity Assay Kit (Solarbio, China) according to the manufacturer’s protocol. The oxidation rate of NADH was spectrophotometrically monitored at 340 nm, and enzyme activity was calculated based on the linear decrease in absorbance over time (ΔA340/min).

### NAD+/NADH ratio assay

The NAD + /NADH ratio in CRC cells across experimental groups was determined using an NAD + /NADH Assay Kit (Beyotime, China) following the manufacturer’s protocol. Absorbance values were spectrophotometrically measured at 450 nm to quantify NAD, NADH, and their ratio.

### Mitochondrial membrane protein extraction

Cells from each experimental group were cultured to 90% confluence and incubated in low-glucose medium for 24 h. To prepare the homogenization buffer (HB), 2.5 mL of 1 M sucrose solution was mixed with 100 μL of 1 M HEPES buffer (pH 7.4), and the volume was adjusted to 10 mL with triple-distilled water. After treatment, cells were washed three times with ice-cold PBS and harvested in 1 mL of pre-chilled HB buffer. Cell suspensions were kept on ice for 10 min to facilitate lysis, followed by 30 passes through a 28-gauge needle to mechanically disrupt cell membranes. The lysate was centrifuged at 1,100 × g for 10 min at 4 °C to remove nuclei and unbroken cells. The supernatant was further centrifuged at 11,000 × g for 15 min at 4 °C to pellet mitochondria. The resulting supernatant (cytosolic fraction) and mitochondrial pellet (resuspended in 200 μL HB buffer) were collected.

### Detection of mitochondrial ROS

Cells from each experimental group were seeded into confocal dishes or 6-well plates and cultured to appropriate density. Cells were incubated with 500 μL of MitoSOX™ Red working solution (5 μM, HY-D1055, MCE) at 37 °C for 20 min. Following incubation, cells in confocal dishes were immediately imaged under a laser scanning confocal microscope. For flow cytometric analysis, cells were detached, centrifuged at 300 × g for 5 min, resuspended in PBS, and analyzed using a flow cytometer.

### Detection of mitochondrial membrane potential assay

Cells from each experimental group were seeded into confocal dishes or 6-well plates and cultured to appropriate density. Cells were then incubated with 1 mL of JC-1 working solution (C2006, Beyotime) at 37 °C for 20 min. Following incubation, cells were washed twice with pre-warmed JC-1 assay buffer. For imaging, cells in confocal dishes were directly visualized under a laser scanning confocal microscope to capture mitochondrial membrane potential-dependent fluorescence shifts. For flow cytometry, cells were detached using trypsin-EDTA, centrifuged at 300×g for 5 min, resuspended in JC-1 assay buffer, and analyzed immediately using a flow cytometer.

### Immunofluorescence staining

To observe LC3 puncta formation and its colocalization with mitochondria, cells from each group were cultured on sterile glass coverslips. After treatment, cells were incubated with 200 nM MitoTracker working solution (100 μL per coverslip, HY-D1783, MCE) at 37 °C for 30 min. Following PBS washes, cells were fixed with ice-cold methanol for 20 min and permeabilized with 0.3% Triton X-100 in PBS for 10 min. Non-specific binding was blocked with 5% BSA for 1 h at room temperature. Cells were then incubated with a primary antibody against LC3B (ab192890, 1:200) overnight at 4 °C. The next day, coverslips were washed with PBS and incubated with DyLight 488-conjugated goat anti-rabbit secondary antibody (A23220, Abbkine) for 1 h at room temperature, followed by nuclear counterstaining with DAPI (1 μg/mL, 10 min). Finally, coverslips were mounted using anti-fade mounting medium and imaged under a confocal microscope with sequential channel acquisition to minimize cross-talk.

### Statistical analysis

Statistical analyses were performed using GraphPad Prism 8.0 and R version 4.3.1. Normality of data distributions was assessed by the Shapiro-Wilk test. Data with *P* > 0.05 were considered normally distributed, and differences between groups were analyzed using Student’s *t*-test. For non-normally distributed data (*P* < 0.05 by Shapiro-Wilk test), the Wilcoxon rank-sum test was applied. A threshold of *P* < 0.05 was used to define statistical significance.

## Supplementary information

Supplementary Figure Legends

Western Blotting

The TME image

Figure S1

Figure S2

Figure S3

Figure S4

Figure S5

Figure S6

Table S1 The Sequences for PPA1 shRNA Knockdown and Overexpression Lentiviral

Table S2 The Sequences for ULK1 overexpression plasmids and phosphorylation site mutant plasmids

Table S3 The clinical characteristics of colorectal cancer patients in in tissue microarrays

Table S4 Metabolomics Expression Matrix Following PPA1 Knockdown

Table S5 Phosphorylation Expression Matrix of Proteins Following PPA1 Knockdown

## Data Availability

Processed metabolomics and phosphoproteomics matrices are available in the supplementary materials (Table S4, S5). Raw data files can be requested from the corresponding author. The download links for additional publicly available datasets are provided in the Methods section. The full length uncropped original western blots are shown in the ‘Supplementary Material’.
