# Supplementary material for: PPA1 promotes oxidative phosphorylation and malignant progression of colorectal cancer under glucose restriction via AMPK/ULK1/FUNDC1-mediated mitophagy
Source: Cell Death Discov. 2025 Nov 28;11:549. doi: 10.1038/s41420-025-02816-y (PMC12663196; doi:10.1038/s41420-025-02816-y)
Supplement: Supplementary file 1 — Supplementary Figure Legends [file 41420_2025_2816_MOESM1_ESM.docx]

**Supplementary Figure Legends**

**Figure S1 Expression of PPA1 in CRC and its correlation with clinical features. (A)** Cells with gene counts > 100 and < 4,000, and mitochondrial contamination < 20% were identified as high-quality cells and retained; **(B)** Dot plot illustrates the expression of marker genes for each cell cluster; **(C)** Volcano plot shows 437 DEGs in epithelial cells between CRC and adjacent normal tissues; **(D)** Heatmap displays the expression of the top 10 DEGs in the oxidative phosphorylation pathway; **(E)** Dot plot demonstrates PPA1 expression across the 9 cell clusters in CRC and adjacent normal tissues; **(F)** Statistical analysis of the mean fluorescence intensity of PPA1 in epithelial cells (EpCAM+) from CRC and adjacent normal tissues; **(G-I)** Relationship between PPA1 expression in epithelial cells of patients with CRC and T stage (G), N stage (H), and TNM stage (I); **(J)** Kaplan-Meier survival analysis showing the association between PPA1 expression levels and overall survival in CRC patients. **: *p* < 0.01, ****: *p* < 0.0001.

**Figure S2 Effects of low-glucose culture conditions and high-glucose culture conditions on CRC cells. (A, B)** Quantification of the inhibition ratio of PPA1 knockdown on cell proliferation(A) and numbers of colonies(B) in CRC cells under low- or high-glucose culture conditions; **(C, D)** Quantification of the inhibition ratio of PPA1 knockdown on number of migrating(C) and invading(D) cells under low- or high-glucose culture conditions; **(E)** Quantification of the inhibition ratio of PPA1 knockdown on migration distance in CRC cells under low- or high-glucose culture conditions; **(F-J)** Quantification of the promotion ratio of PPA1 knockdown on cell proliferation(F), numbers of colonies(G), number of migrating(H) and invading(I) cells, and migration distance(J) under low- or high-glucose culture conditions; **(K-L)** Expression levels(K) and statistical analysis(L) of PPA1 in CRC cells under low- or high-glucose culture conditions; All data are presented as Mean ± SD, n=3. *: *p*<0.05, **: *p*<0.01, ***: *p*<0.001, ****: *p*<0.0001.

**Figure S3 Effects of PPA1 knockdown on phosphorylation in CRC cells. (A)** Volcano plot illustrating 665 differentially phosphorylated sites in HCT116 cells after PPA1 knockdown; **(B)** Bubble plot showing pathways enriched with 154 differentially phosphorylated proteins; **(C)** Heatmap displaying differential phosphorylation expression in the mitophagy pathway; **(D-I)** Quantification of protein and phosphorylated site expression levels in HCT8 (D-F) and HCT116 (G-I) cells (Mean ± SD, n=3); **(J, K)** Quantitative analysis of LC3 fluorescence(J) and its co-localization(K) with mitochondria in HCT8 cells; **(L, M)** Quantitative analysis of LC3 fluorescence(L) and its co-localization(M) with mitochondria in HCT116 cells; **(N)** Confocal microscopy analysis of mitochondrial membrane potential in CRC cells (Mean ± SD, n=3, 200× magnification); *:  *p*<0.05, **:  *p*<0.01, ***: *p*<0.001, ****: *p*<0.0001.

**Figure S4 Effects of the ULK1 agonist LYN-1604 on CRC proliferation, migration, and invasion.** Under glucose-restricted conditions, **(A-D)** Quantitative analysis of LC3 fluorescence and its co-localization with mitochondria in HCT8 and HCT116 cells; **(E)** CCK-8 assay evaluating the impact of the ULK1 agonist LYN-1604 (1μM) on the proliferative capacity of CRC cells; **(F)** Colony formation assay demonstrating the effect of LYN-1604 (1μM) on the clonogenic ability of CRC cells; **(G, H)** Wound healing assay illustrating the influence of LYN-1604 (1μM) on the migratory capacity of HCT8 cells (G) and HCT116 cells (H); **(I, J)** Transwell assay analyzing the migratory and invasive abilities of HCT8 cells (I) and HCT116 cells (J) treated with LYN-1604 (1μM). All data are presented as Mean ± SD, n=3. *: *p*<0.05, **: *p*<0.01, ***: *p*<0.001, ****: *p*<0.0001.

**Figure S5 Effects of the AMPK agonist GSK621 on ULK1/FUNDC1-** **mediated mitophagy. (A-F)** Quantitative analysis of mitophagy-related proteins and phosphorylation levels HCT8(A, C, D) and HCT116(B, E, F) cells; **(G-J)** Quantitative analysis of LC3 fluorescence and its co-localization with mitochondria in HCT8(G, H) and HCT116 cells (I, J); All data are presented as Mean ± SD, n=3. *: *p*<0.05, **: *p*<0.01, ***: *p*<0.001, ****: *p*<0.0001.

**Figure S6 Effects of the AMPK agonist GSK621 on CRC proliferation, migration, and invasion. (A, B)** CCK-8 assay evaluating the impact of the **AMPK agonist GSK621** (30μM) on the proliferative capacity of HCT8(A) and HCT116(B) cells; **(C)** Colony formation assay demonstrating the effect of **GSK621** (30μM) on the clonogenic ability of CRC cells; **(D, E)** Transwell assay analyzing the migratory and invasive abilities of HCT8 cells (D) and HCT116 cells (E) treated with **GSK621** (30μM). **(F, G)** Wound healing assay illustrating the influence of **GSK621** (30μM) on the migratory capacity of HCT8 cells (F) and HCT116 cells (G); All data are presented as Mean ± SD, n=3. *: *p*<0.05, **: *p*<0.01, ***: *p*<0.001, ****: *p*<0.0001.
