# Supplementary material for: PPA1 promotes oxidative phosphorylation and malignant progression of colorectal cancer under glucose restriction via AMPK/ULK1/FUNDC1-mediated mitophagy
Source: Cell Death Discov. 2025 Nov 28;11:549. doi: 10.1038/s41420-025-02816-y (PMC12663196; doi:10.1038/s41420-025-02816-y)
Supplement: Supplementary file 2 — Western Blotting [file 41420_2025_2816_MOESM2_ESM.pdf]

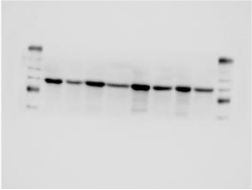 PPA1 33Kda 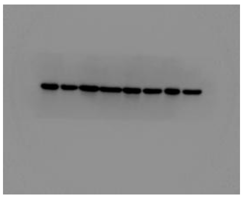 GAPDH 37Kda

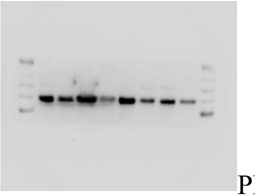 PPA1 33Kda 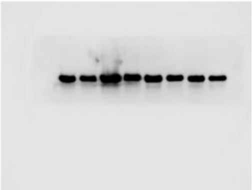 GAPDH 37Kda

Figure1E

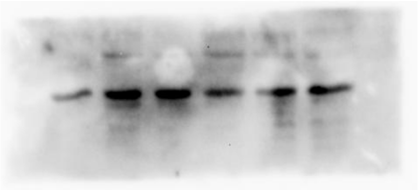 PPA1 33Kda 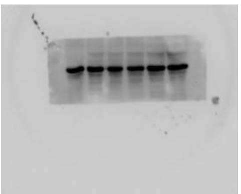 GAPDH 37Kda

Figure1H

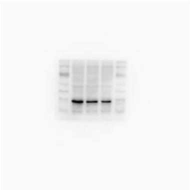 PPA1 33Kda 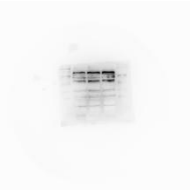 E-Ca 130Kda

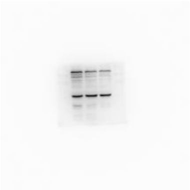 N-Ca130 Kda β-actin 42kDA

Figure2I

 PPA1 33Kda 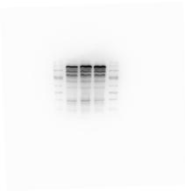 E-Ca 130Kda

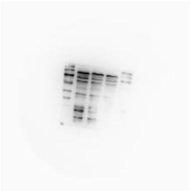 N-Ca130 Kda β-actin 42kDA

Figure2J

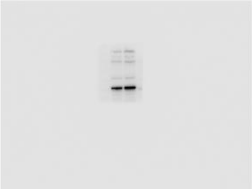
PPA1 33Kda
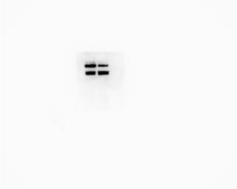
E-Ca 130Kda
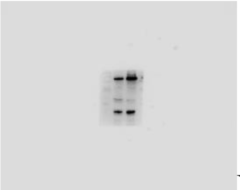
N-Ca130 Kda
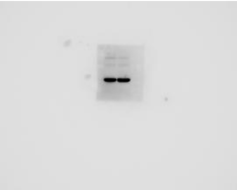
β-actin 42Kda

Figure 3I

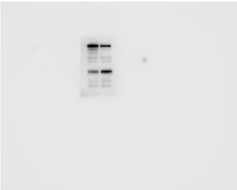
PPA1 33Kda
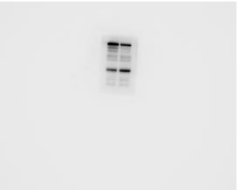
E-Ca 130Kda
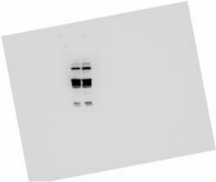
N-Ca130 Kda
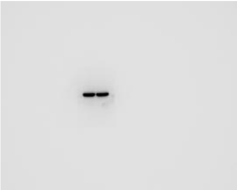
β-actin 42Kda

Figure 3J

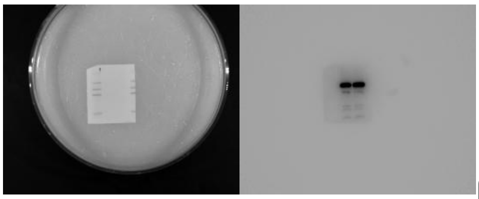
β-actin 42Kda
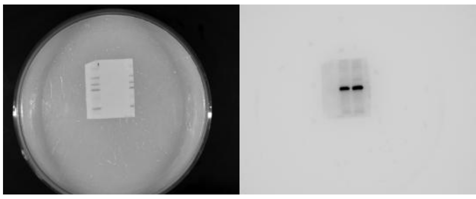
PPA1 33Kda

Figure S2K HCT8

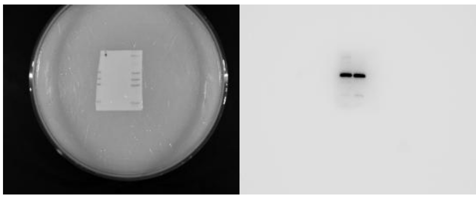
β-actin 42Kda

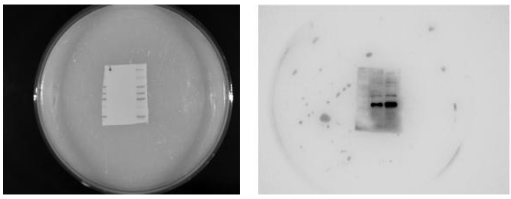

PPA1 33Kda

Figure S2K HCT116

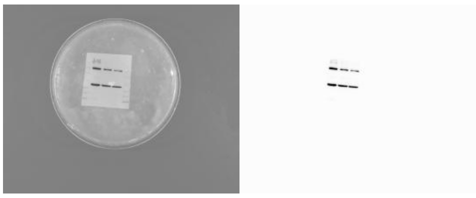

ULK1-Ser 467 130Kda

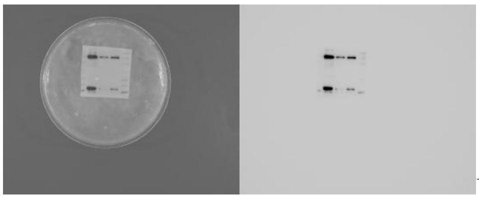

ULK1-Ser 556 130Kda

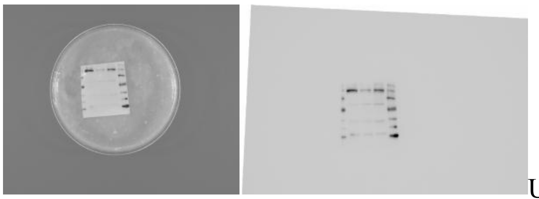

ULK1-Ser 638 130Kda

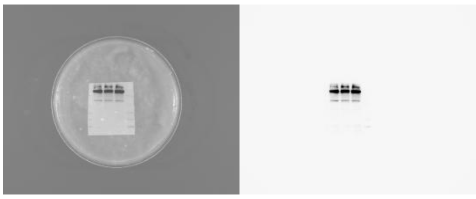

ULK1 130Kda

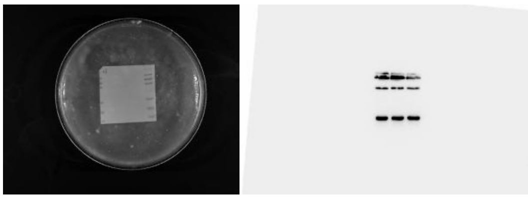

FUNDC1 17Kda

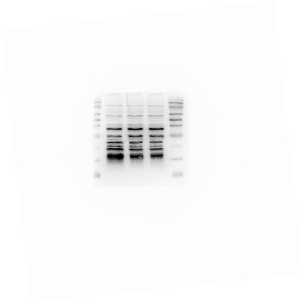

FUNDC1-Ser17 17Kda

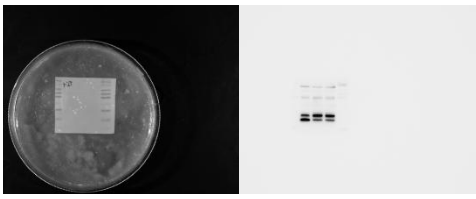

LC3 14/16Kda

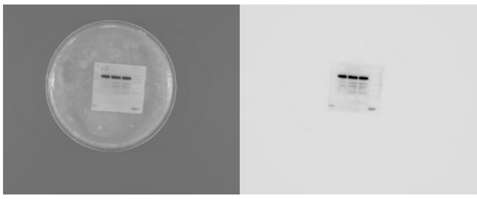

β-actin 42Kda

Figure 5A

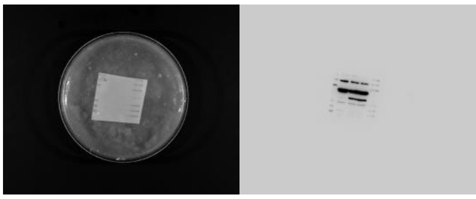

ULK1-Ser 467 130Kda

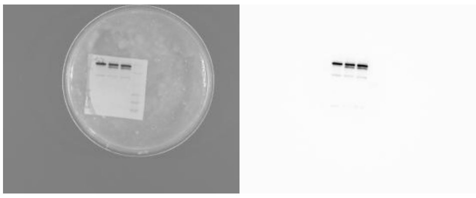

ULK1 130Kda

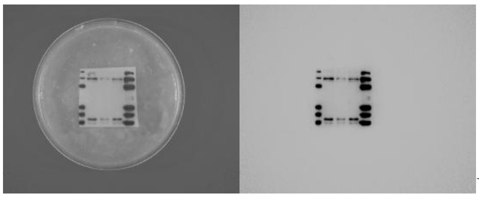

ULK1-Ser 556 130Kda

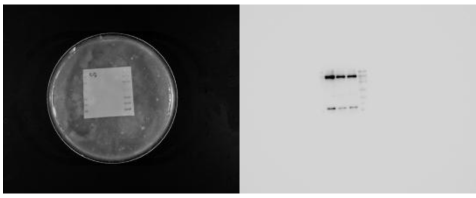

ULK1-Ser 638 130Kda

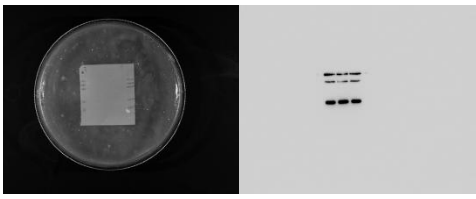

FUNDC1 17Kda

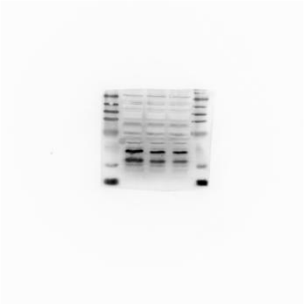

FUNDC1-Ser17 17Kda

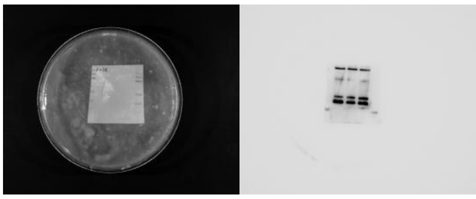

LC3 14/16Kda

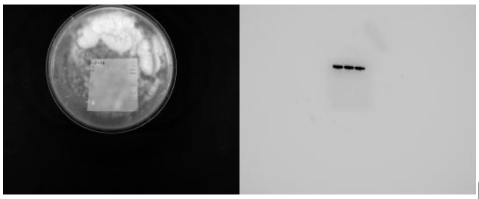

β-actin 42Kda

Figure 5B

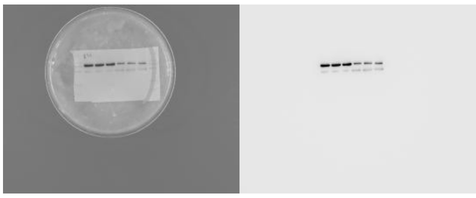

β-actin 42Kda

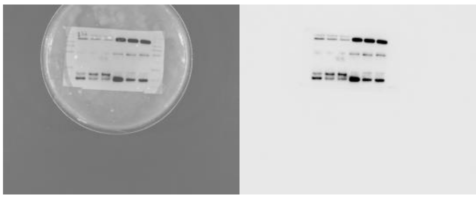

LC3 14/16Kda

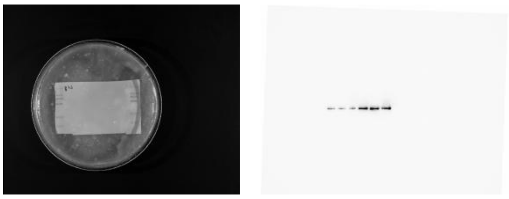

VDAC1 35 Kda

Figure 5C

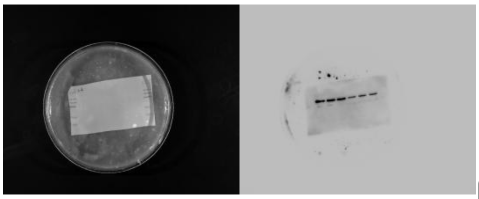
β-actin 42Kda

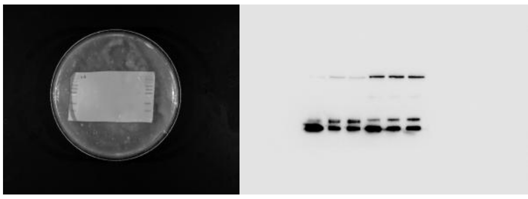
LC3 14/16Kda

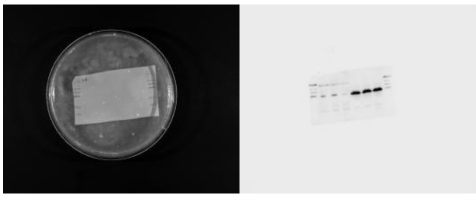
VDAC1 35 Kda

Figure 5D

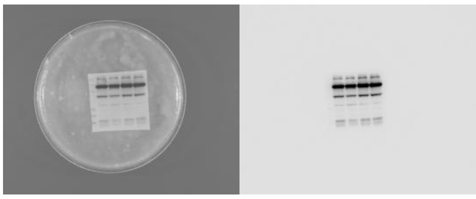
ULK1 130Kda

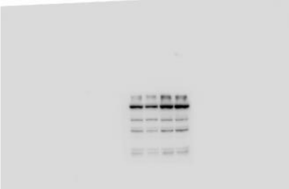
ULK1-Ser 467 130Kda

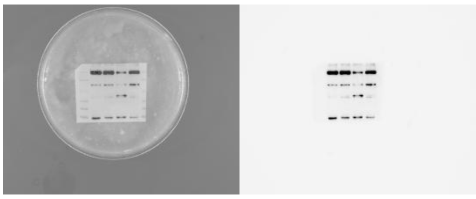
ULK1-Ser 556 130Kda

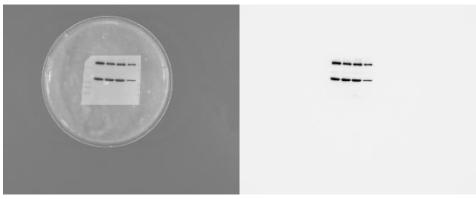
ULK1-Ser 638 130Kda

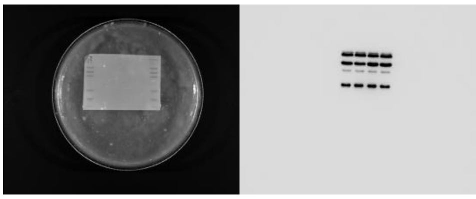
FUNDC1 17Kda

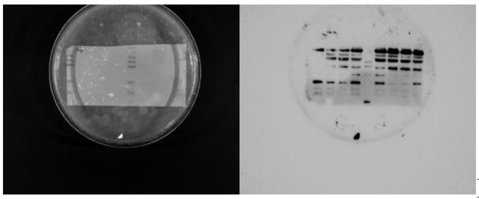
FUNDC1-Ser17 17Kda

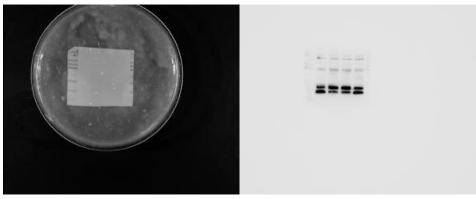
LC3 14/16Kda

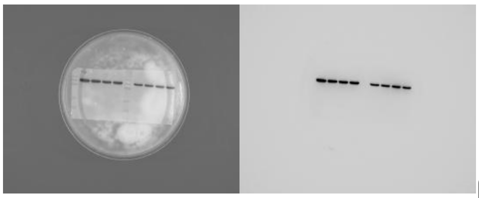
β-actin 42Kda

Figure 6A HCT8

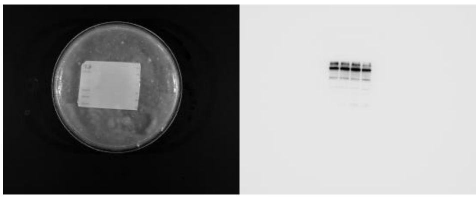
ULK1 130Kda

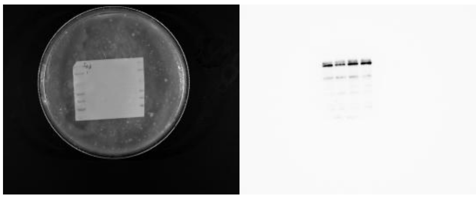
ULK1-Ser 467 130Kda

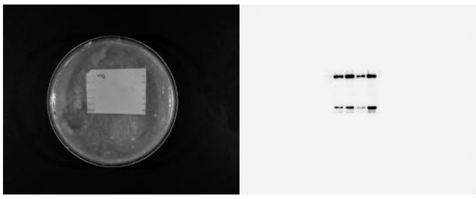
ULK1-Ser 556 130Kda

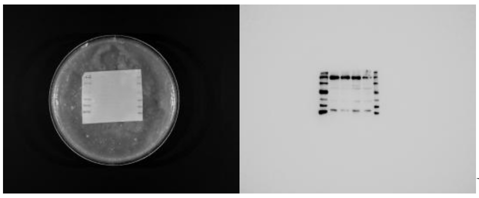
ULK1-Ser638 130Kda

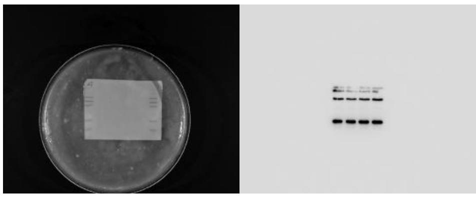
FUNDC1 17Kda

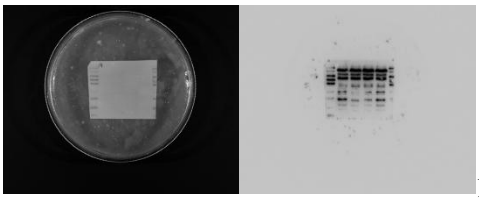

FUNDC1-Ser17 17Kda

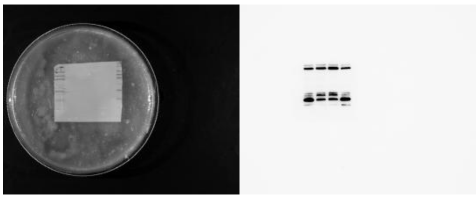

LC3 14/16Kda

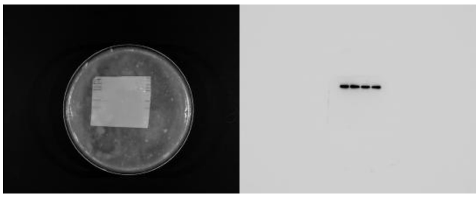

β-actin 42Kda

Figure 6A HCT116

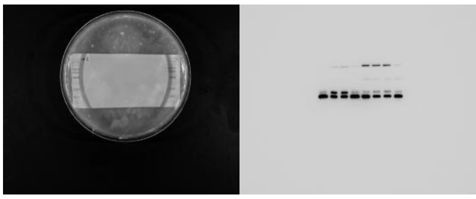

LC3 14/16Kda

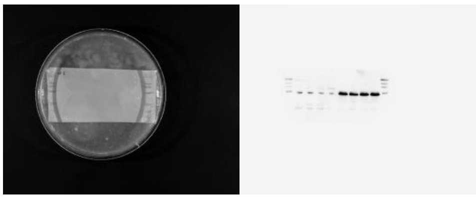

VDAC1 35 Kda

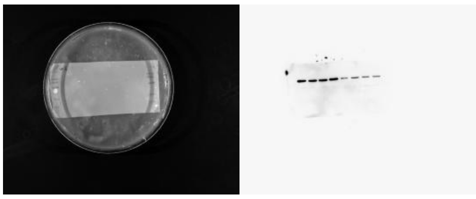

β-actin 42Kda

Figure 6B

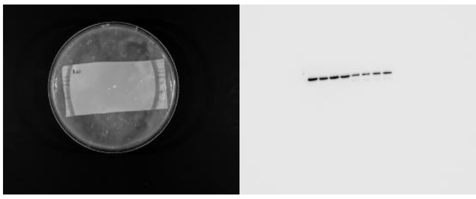

β-actin 42Kda

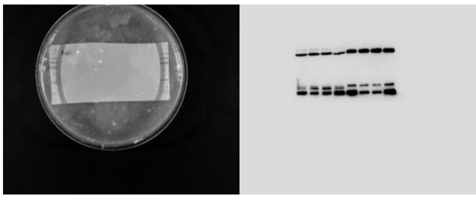
LC3 14/16Kda

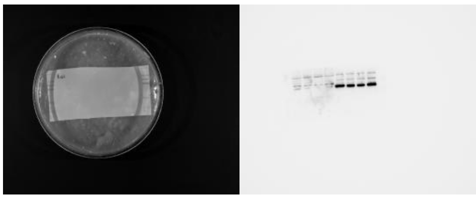
VDAC1 35 Kda

Figure 6C

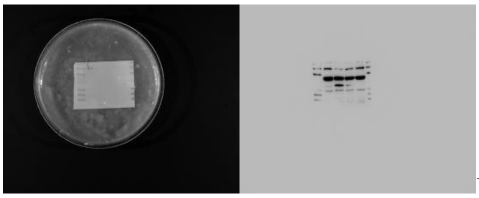
ULK1-Ser 467 130Kda

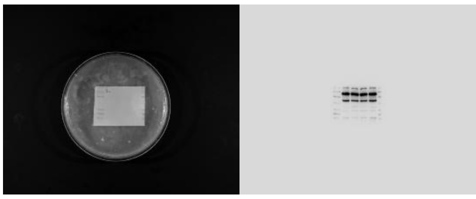
ULK1 130Kda

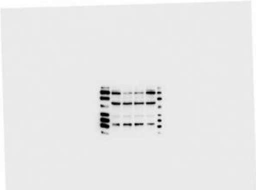
ULK1-Ser556 130Kda

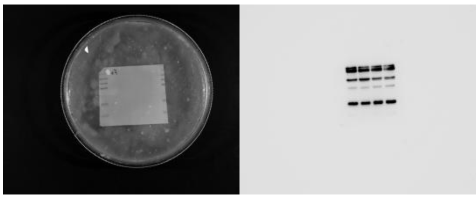
FUNDC1 17Kda

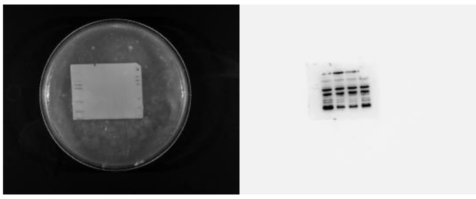
FUNDC1-Ser17 17Kda

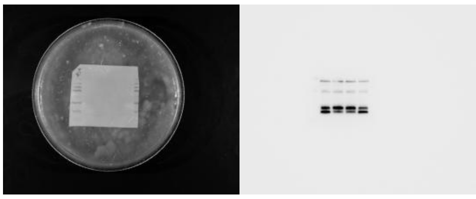
LC3 14/16Kda

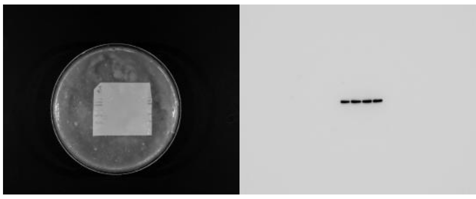
β-actin 42Kda

Figure 7A HCT8

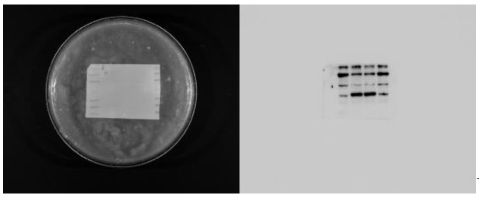
ULK1-Ser 467 130Kda

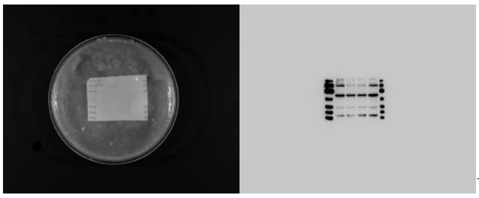
ULK1-Ser 556 130Kda

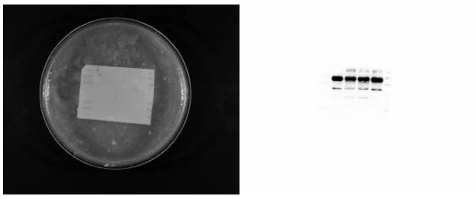
ULK1 130Kda

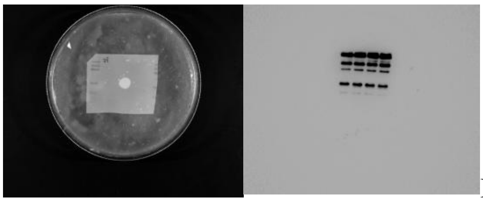
FUNDC1 17Kda

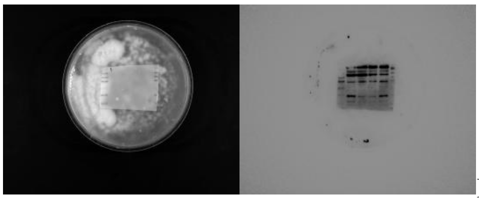
FUNDC1-ser17 17Kda

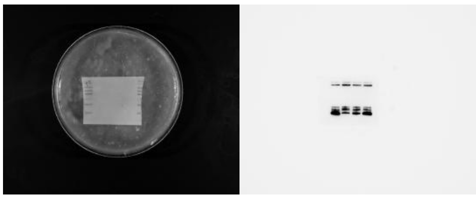
LC3 14/16Kda

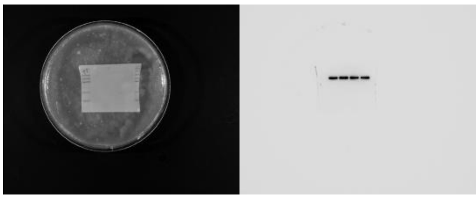
β-actin 42Kda

Figure 7A HCT116

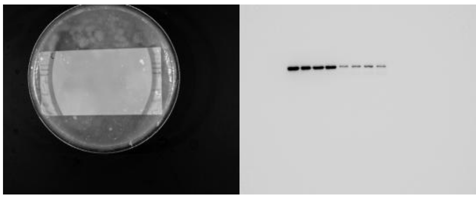
β-actin 42Kda

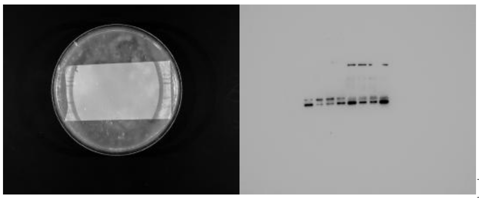
LC3 14/16Kda

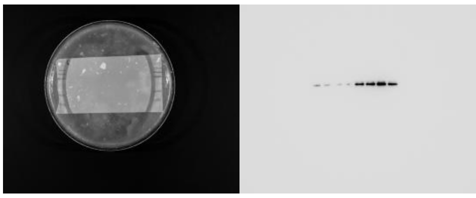
VDAC1 35 Kda

Figure 7B

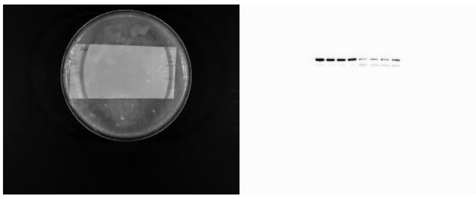
β-actin 42Kda

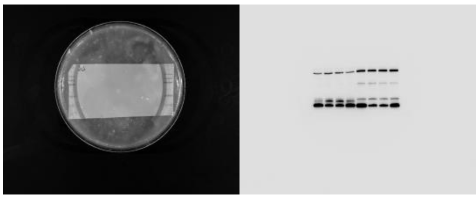
LC3 14/16Kda

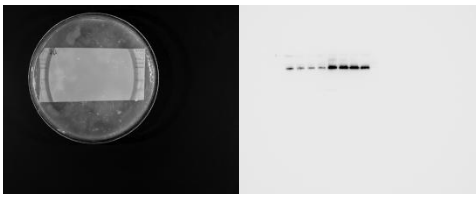
VDAC1 35 Kda

Figure 7C

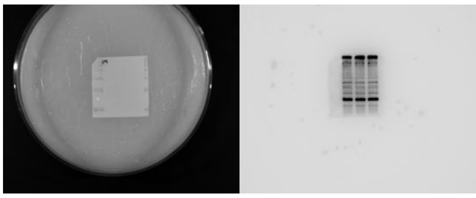
mTOR 289Kda

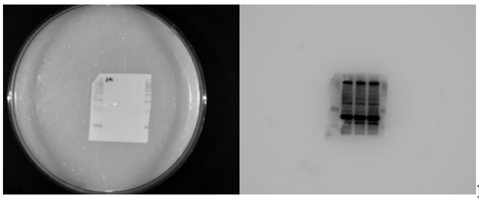
mTOR-Ser2448 289Kda

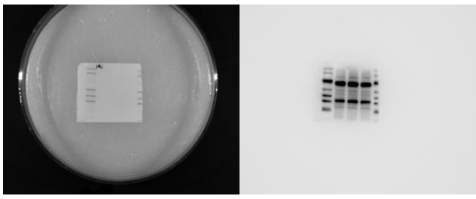
AMPK 62Kda

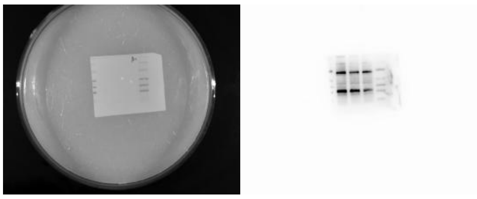
AMPK-Thr 172 64Kda

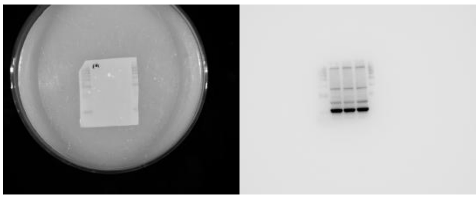
β-actin 42Kda

Figure 8A HCT8

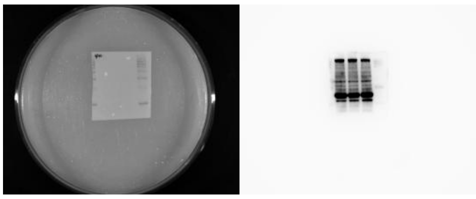
mTOR 289Kda

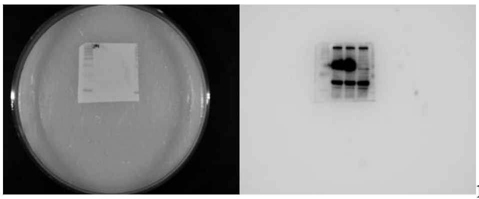
mTOR-Ser2448 289Kda

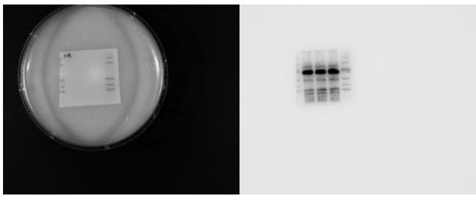
AMPK 62Kda

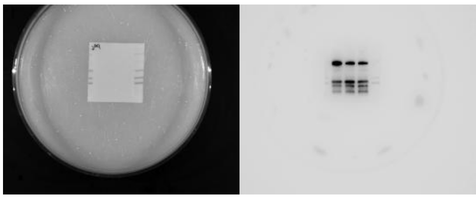
AMPK-Thr 172 64Kda

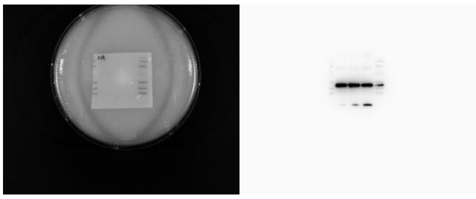

β-actin 42Kda

Figure 8A HCT116

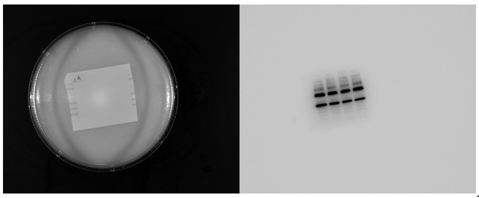

AMPK 62Kda

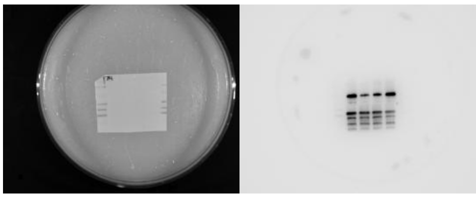

AMPK-Thr 172   64Kda

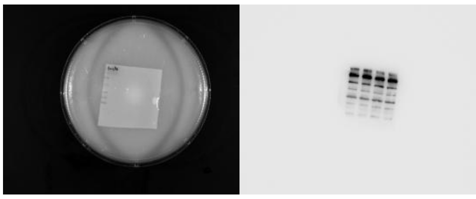

ULK1 130Kda

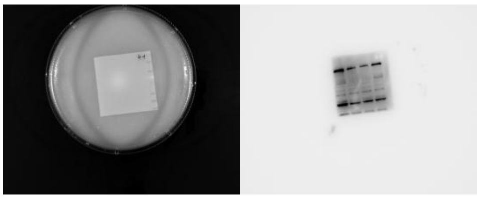

ULK1-Ser467 130Kda

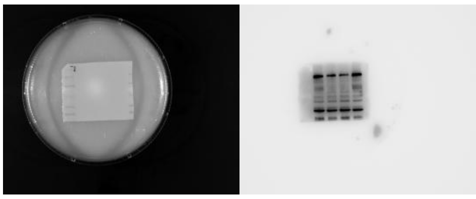

ULK1-Ser556 130Kda

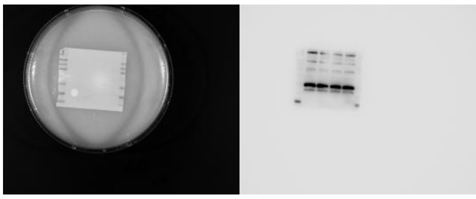

FUNDC1 17Kda

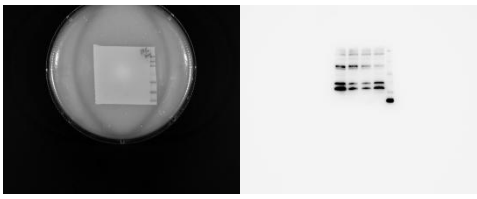

FUNDC1-ser17 17Kda

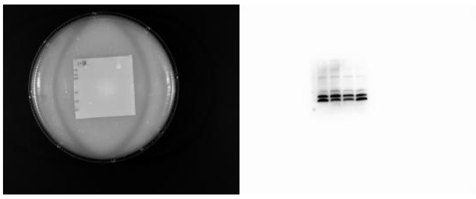
LC3 14/16Kda

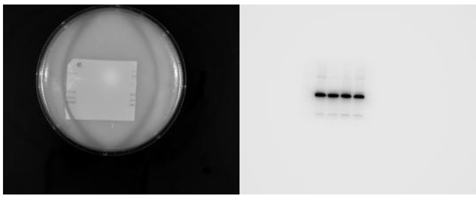
β-actin 42Kda

Figure 8C HCT8

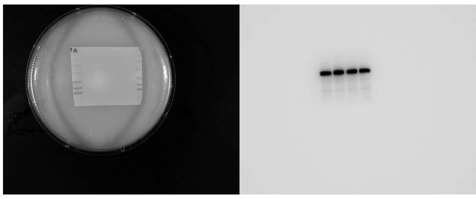
AMPK 62Kda

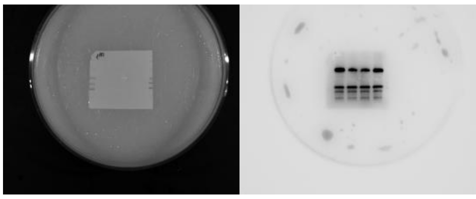
AMPK-Thr 172   64Kda

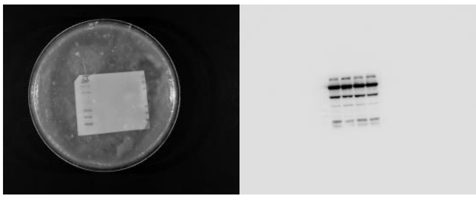
ULK1 130Kda

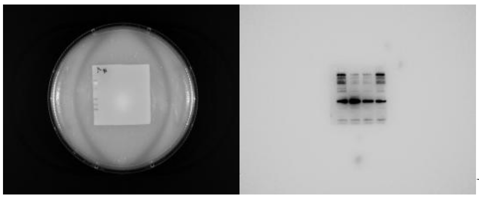
ULK1-Ser467 130Kda

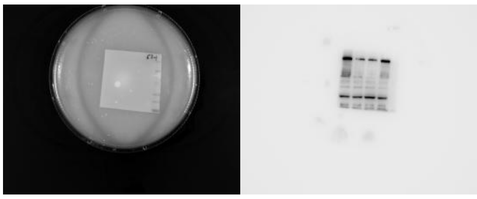
ULK1-Ser556 130Kda

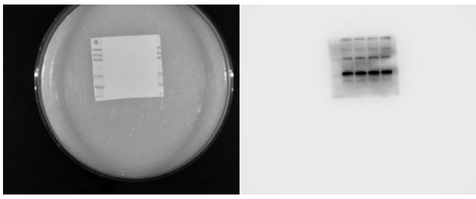

FUNDC1 17Kda

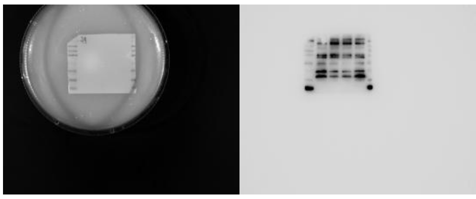

FUNDC1-ser17 17Kda

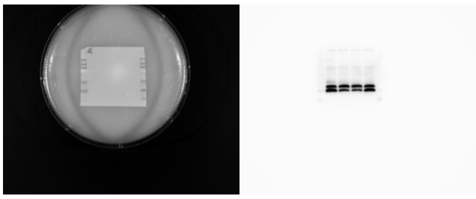

LC3 14/16Kda

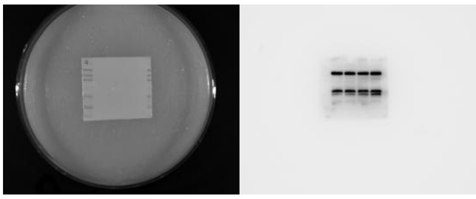

β-actin 42Kda

Figure 8C HCT116

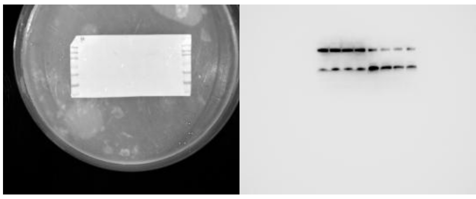

β-actin 42Kda

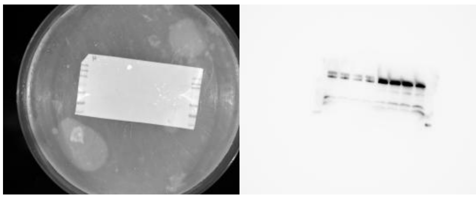

VDAC1 35 Kda

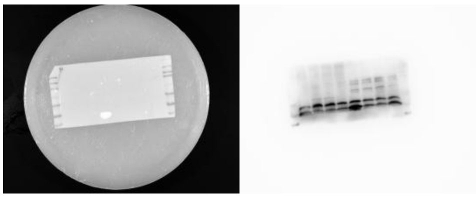

LC3 14/16Kda

Figure 8D HCT8

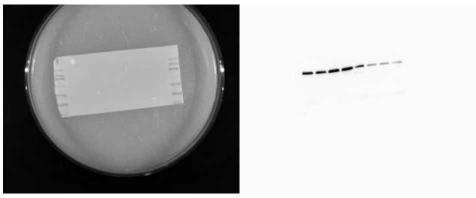
β-actin 42Kda

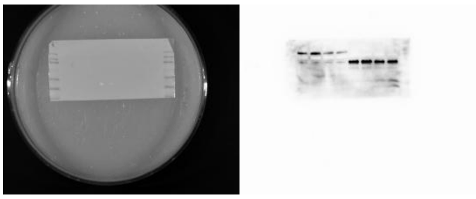
VDAC1 35 Kda

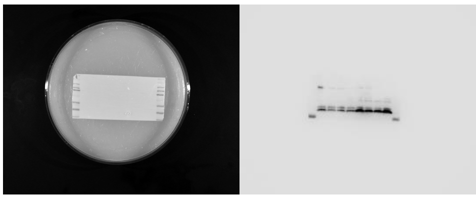
LC3 14/16Kda

Figure 8D HCT116

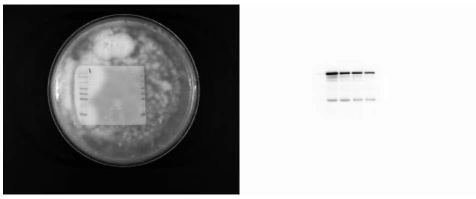

E-Ca 130Kda

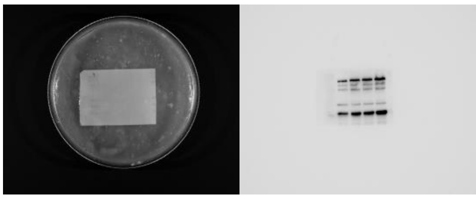

PPA1 33Kda N-ca 130kda

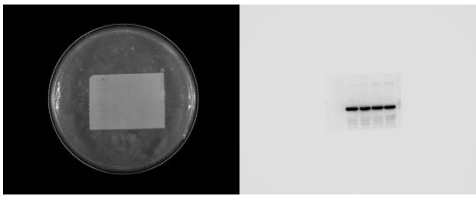

β-actin 42Kda

Figure 9D

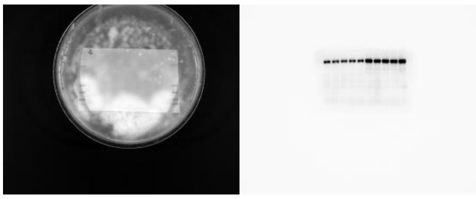

E-Ca 130Kda

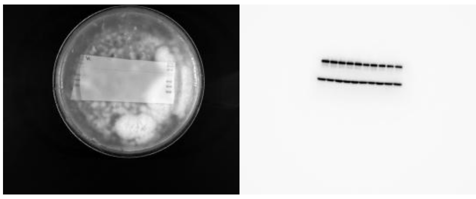

N-ca 130Kda β-actin 42Kda

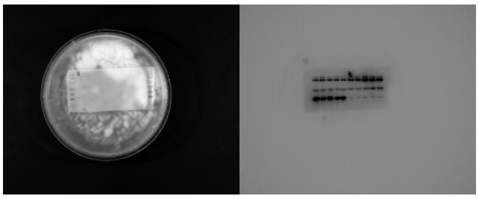

PPA1 33Kda

Figure 9H

Replicate blots：

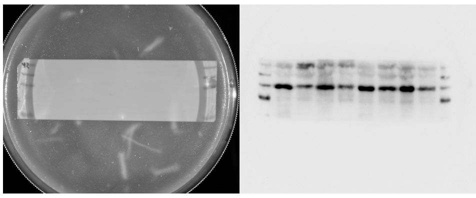

PPA1 33Kda

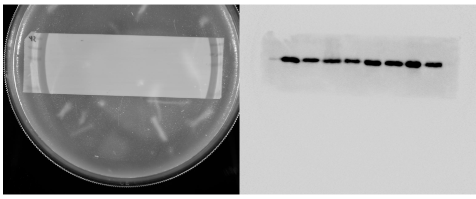

GAPDH 37Kda

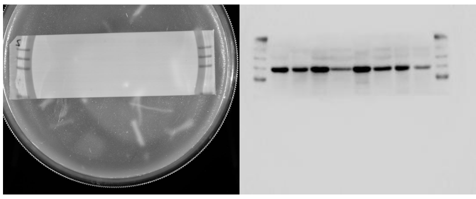

PPA1 33Kda

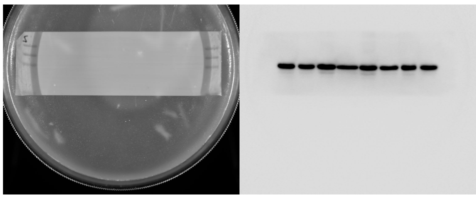

GAPDH 37Kda

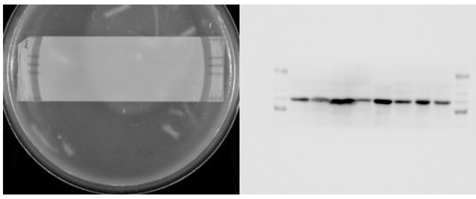

PPA1 33Kda

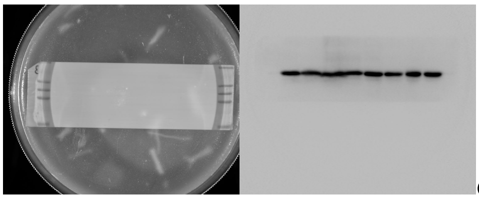

GAPDH 37Kda

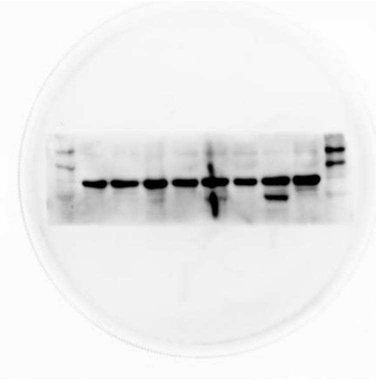

GAPDH 37Kda

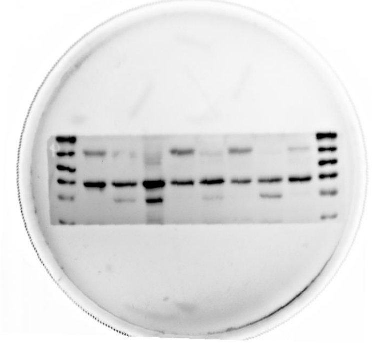

PPA1 33Kda

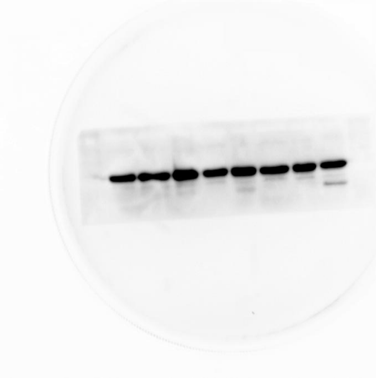

GAPDH 37Kda

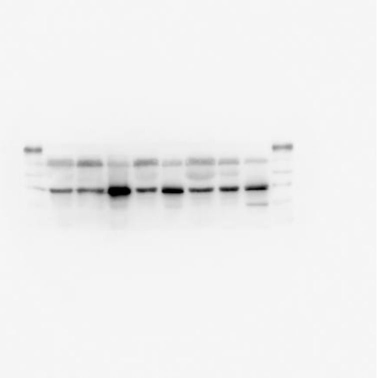

PPA1 33Kda

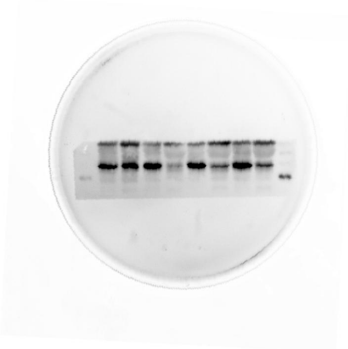

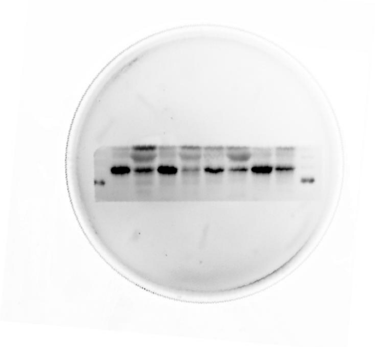

PPA1 33Kda

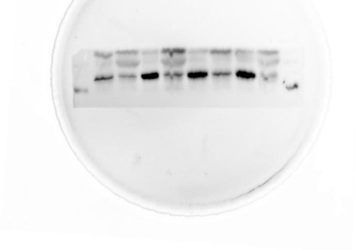

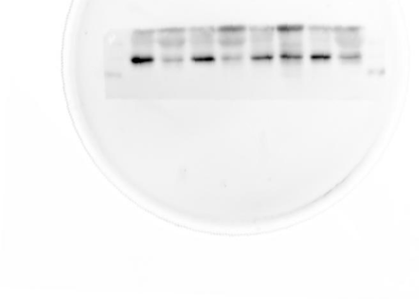

PPA1 33Kda

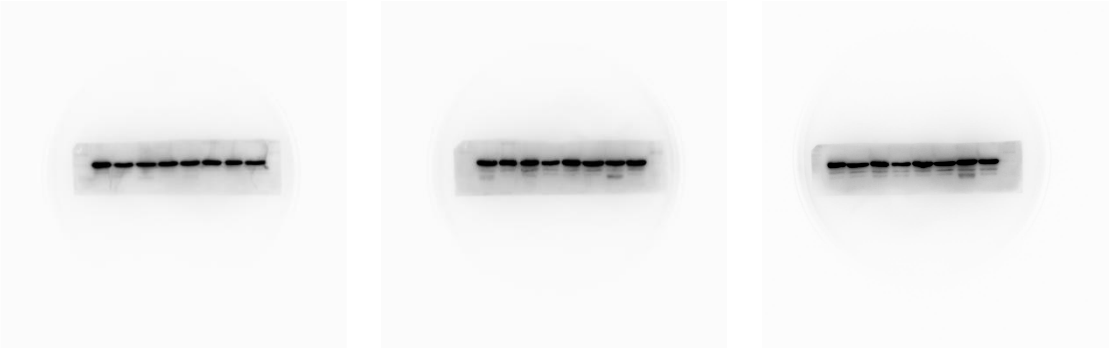

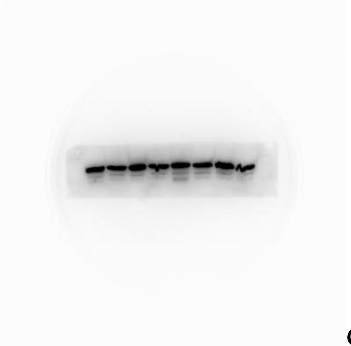

GAPDH 37Kda

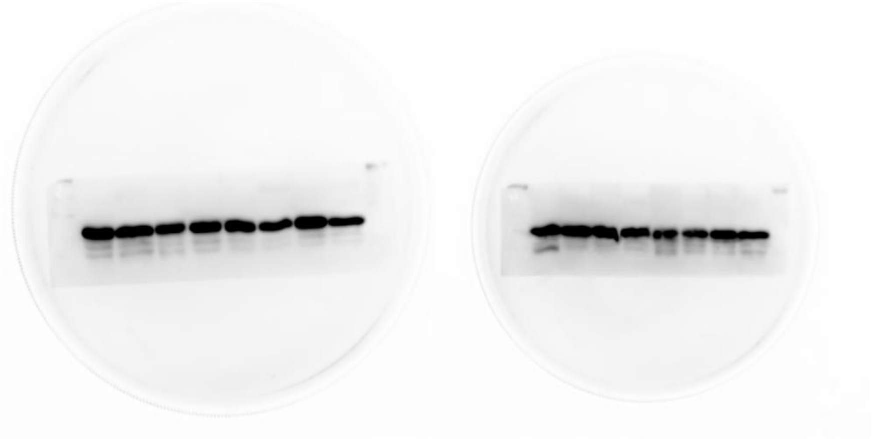

GAPDH   37Kda

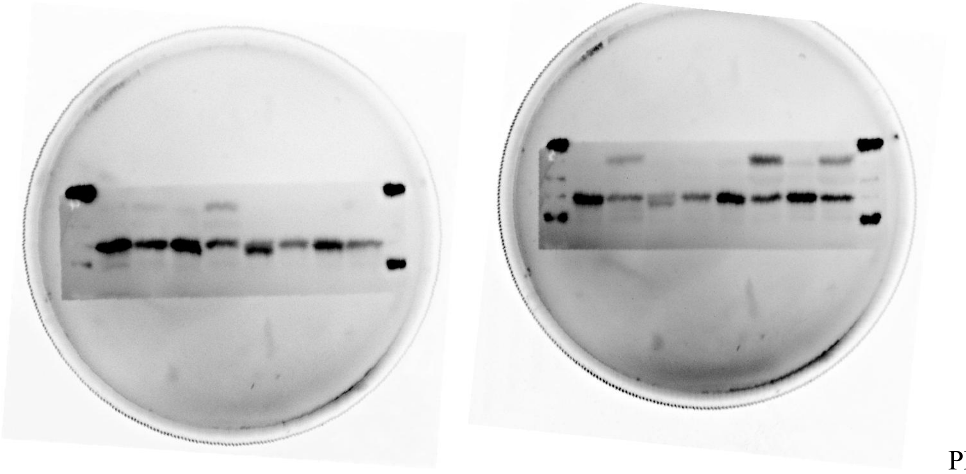

PPA1 33Kda

Figure 1E

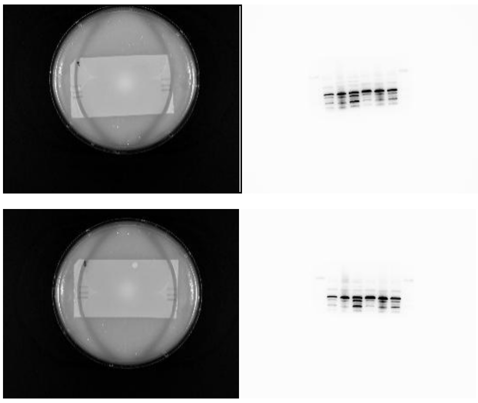

PPA1 33Kda

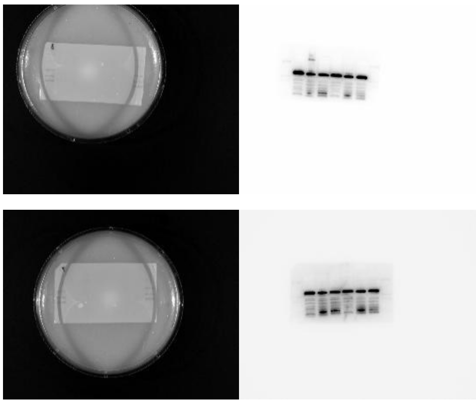

GAPDH 37Kda

Figure 1H

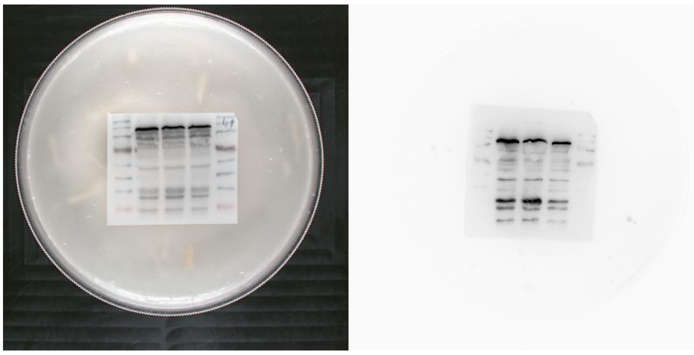

E-ca 130Kda

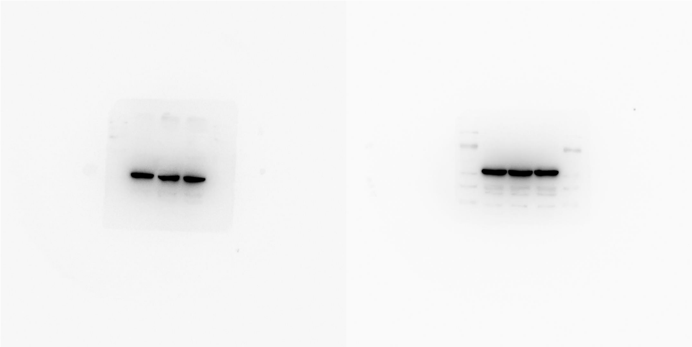

β-actin 42Kda

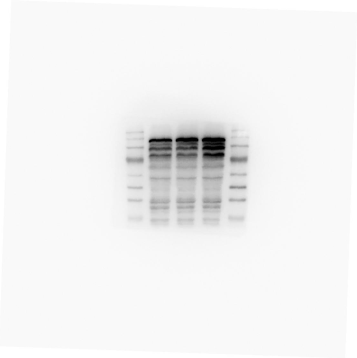
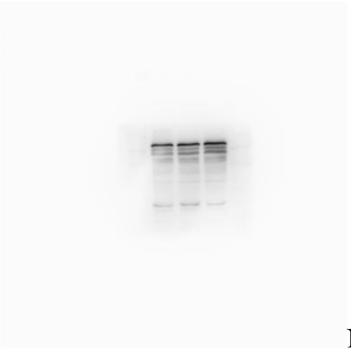
N-ca 130Kda

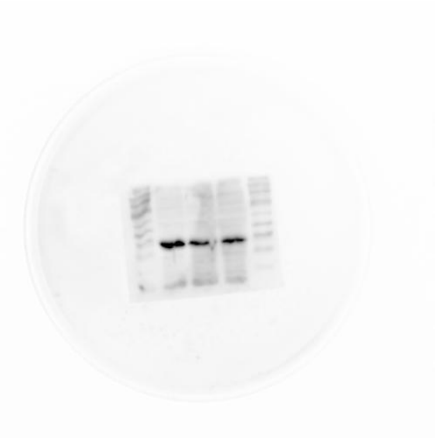
PPA1 33Kda

Figure 2I

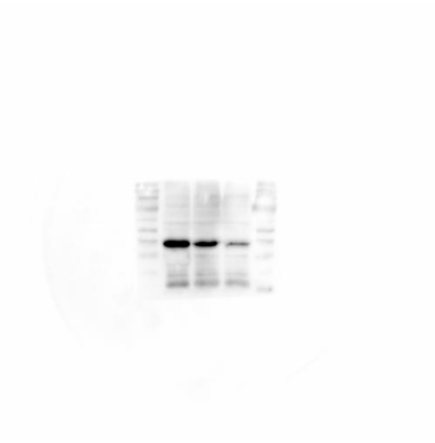
PPA1 33Kda

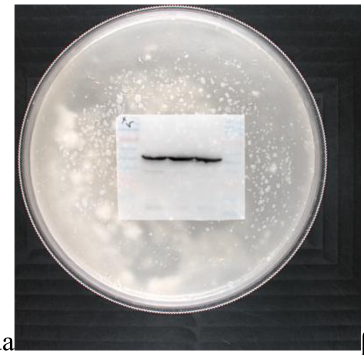
β-actin 42Kda

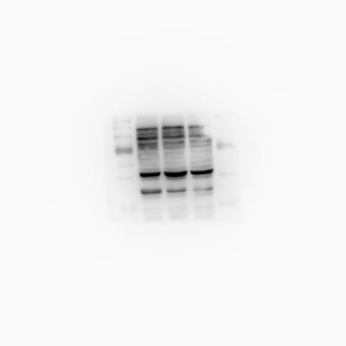
β-actin 42Kda

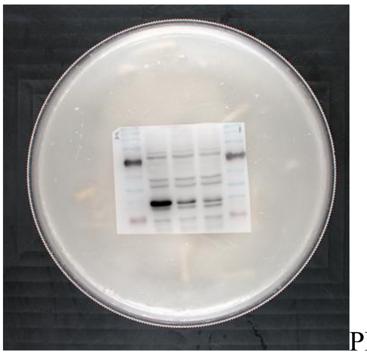
PPA1 33Kda

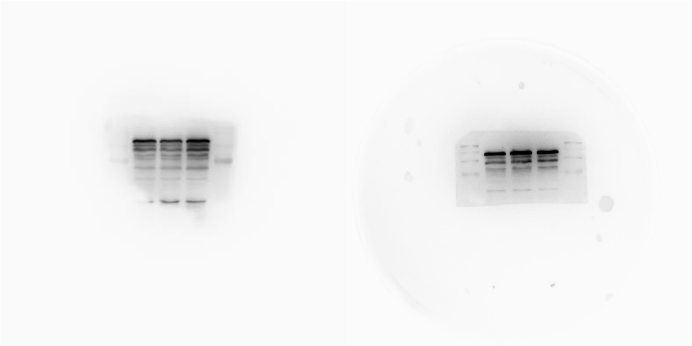
E-ca 130Kda

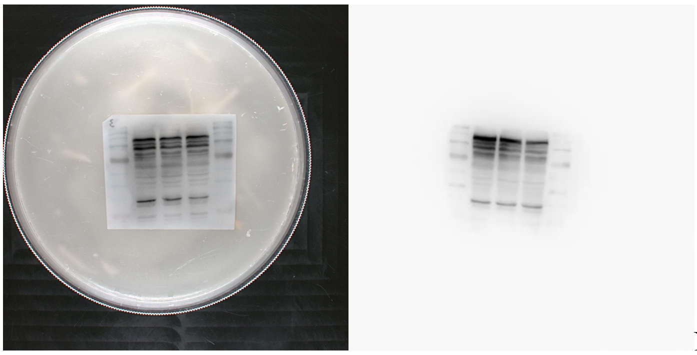
N-ca 130Kda

Figure 2J

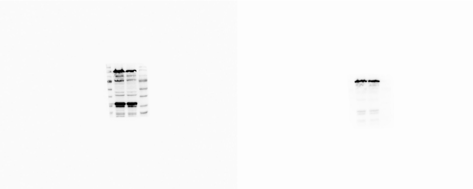
E-ca 130Kda

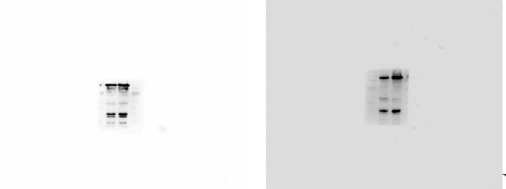
N-ca 130Kda

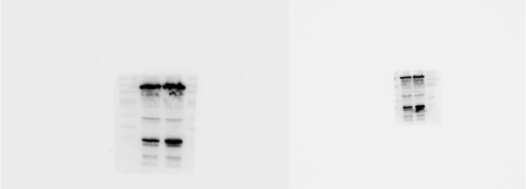
PPA1 33Kda

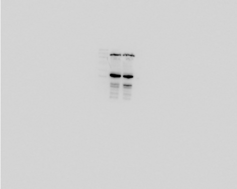
β-actin 42Kda

Figure 3I

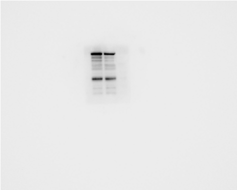
E-ca 130Kda
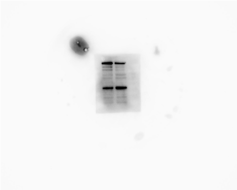
PPA1 33Kda

β-actin 42Kda
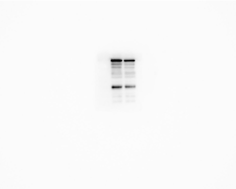
E-ca 130Kda
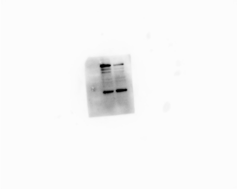
PPA1 33Kda

β-actin 42Kda
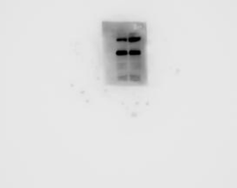

N-ca 130Kda

Figure 3J

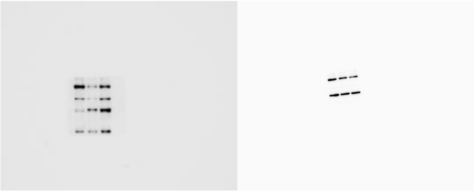
ULK1-Ser467 130Kda

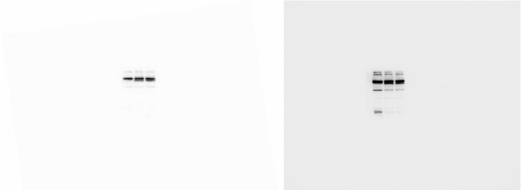
ULK1 130Kda

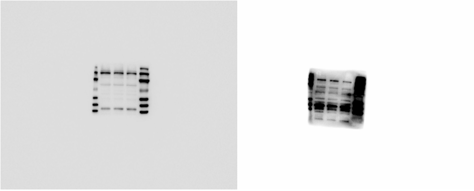
ULK1-Ser556 130Kda

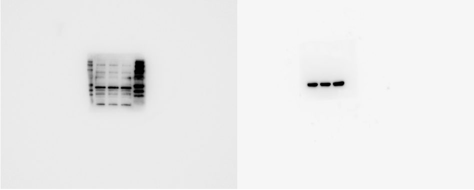
β-actin 42Kda

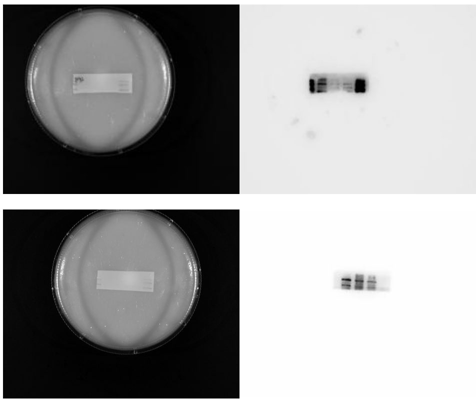
ULK1-Ser638 130Kda

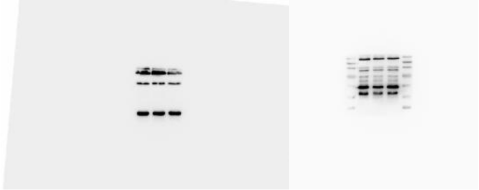
FUNDC1 17Kda

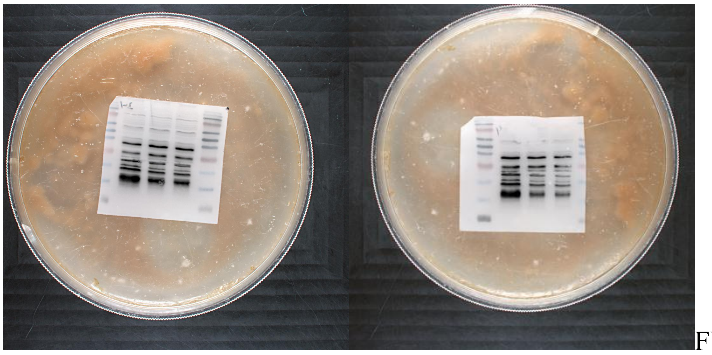
FUNDC1-ser17 17Kda

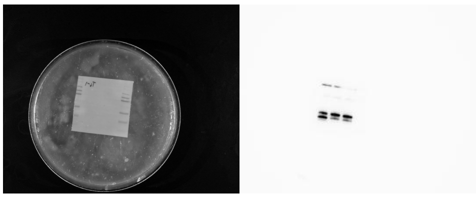

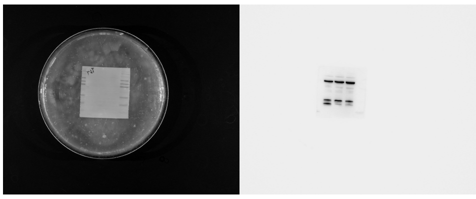

LC3 14/16Kda

Figure 5A

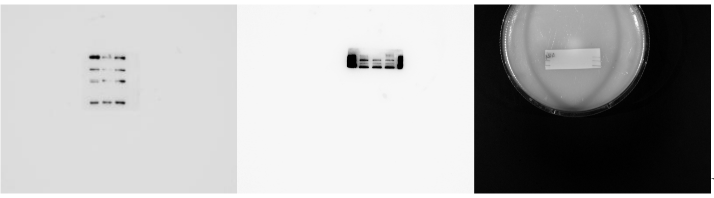

ULK1-Ser467 130Kda

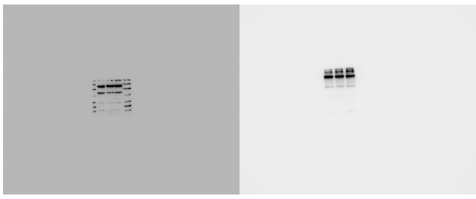

ULK1 130Kda

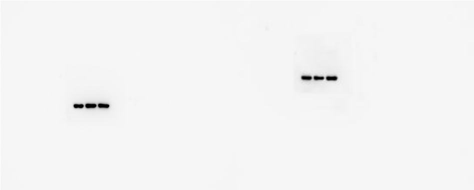

β-actin 42Kda

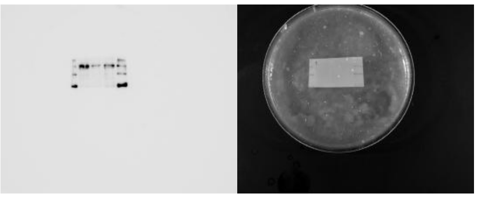

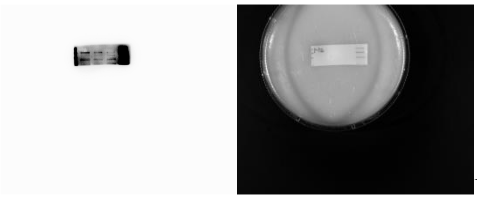

ULK1-Ser556 130Kda

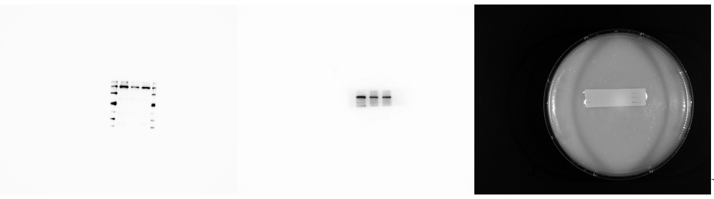

ULK1-Ser638 130Kda

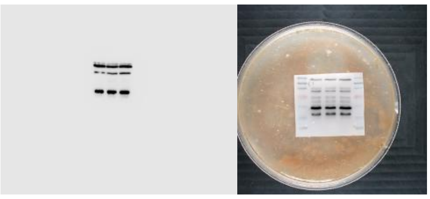
FUNDC1 17Kda

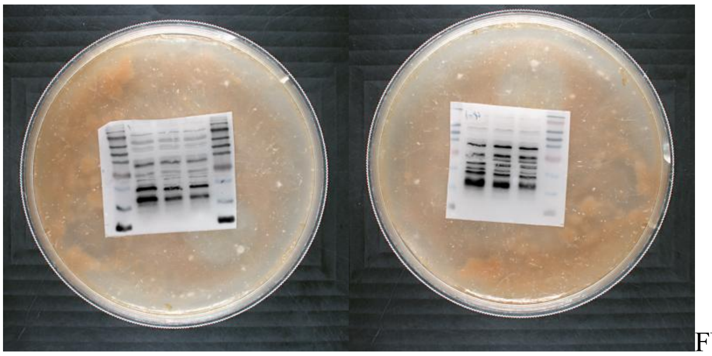
FUNDC1-Ser17 17Kda

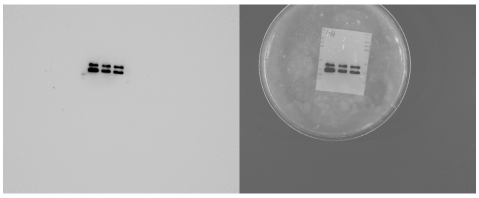

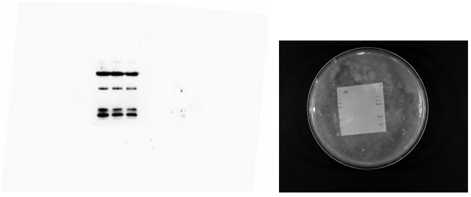
LC3 14/16Kda

Figure 5B

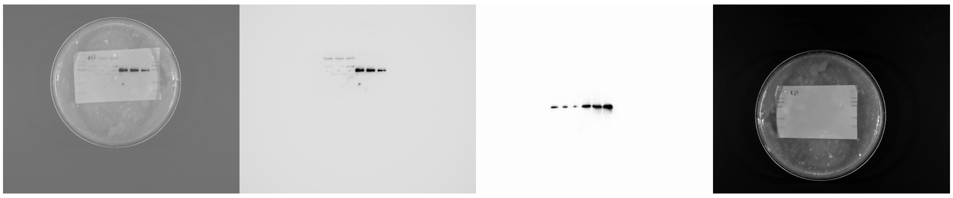

VDAC1 35Kda

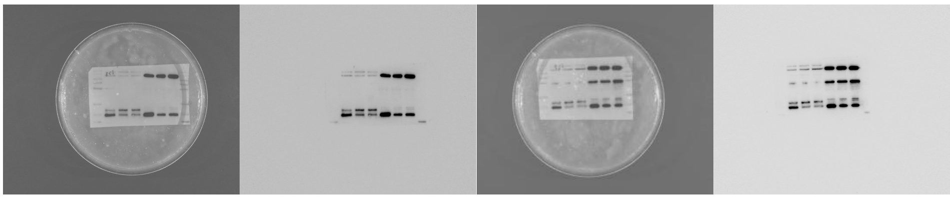

LC3 14/16Kda

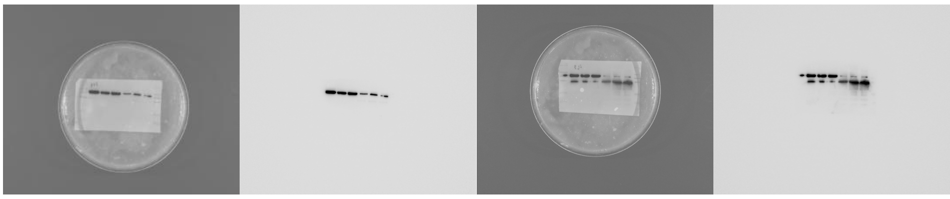

β-actin 42Kda

Figure 5C

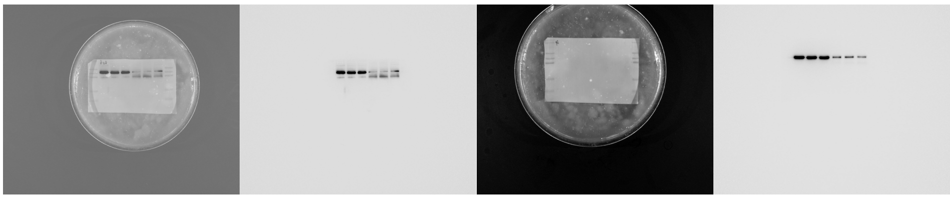

β-actin 42Kda

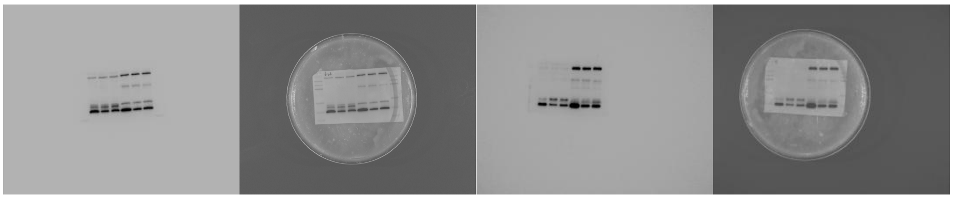

LC3 14/16Kda

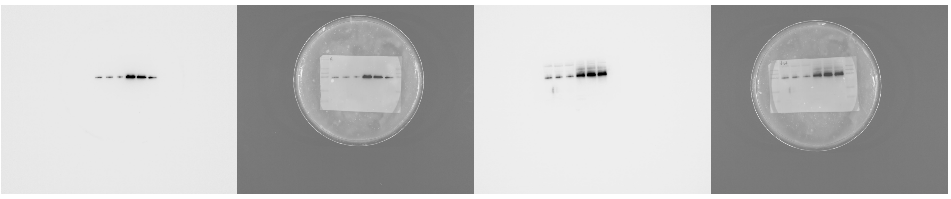

VDAC1 35Kda

Figure 5D

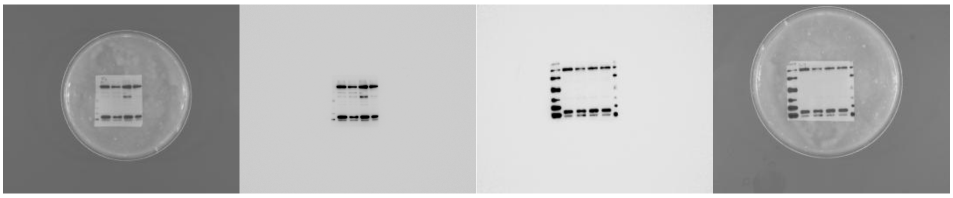

ULK1-Ser467 130Kda

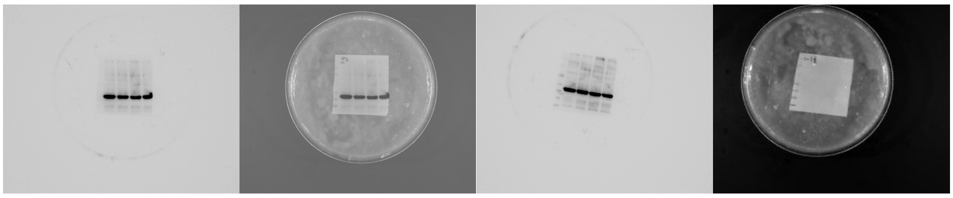

β-actin 42Kda

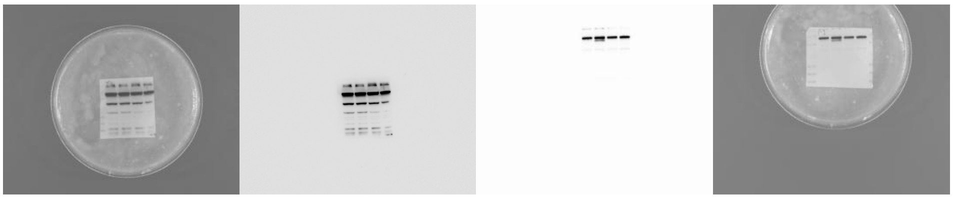

ULK1 130Kda

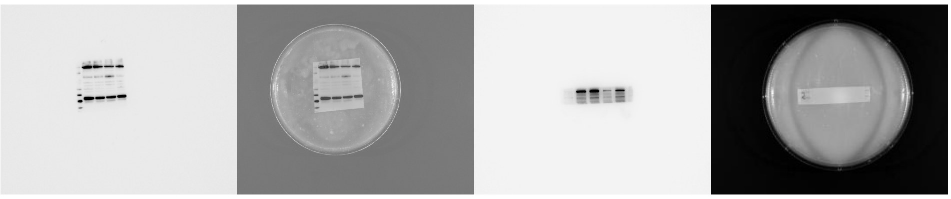

ULK1-Ser556 130Kda

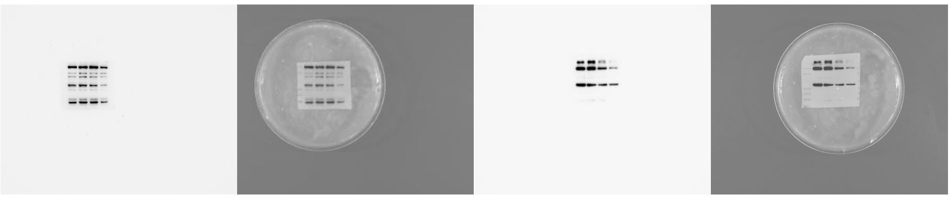

ULK1-Ser638 130Kda

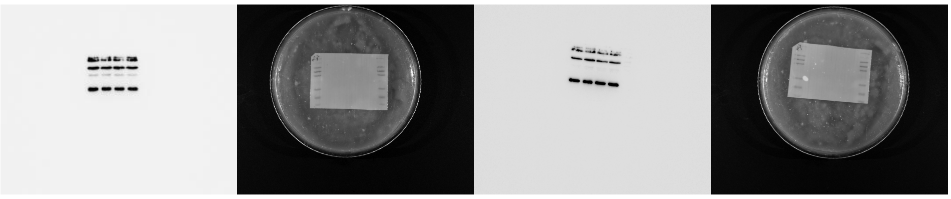

FUNDC1 17Kda

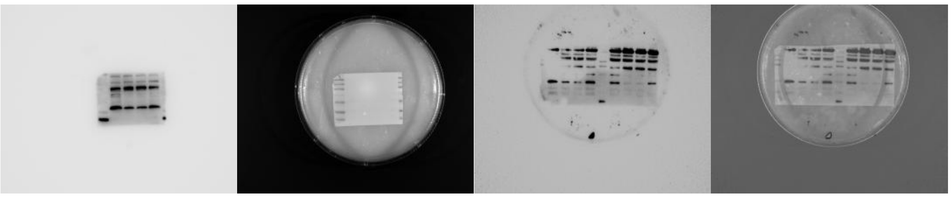

FUNDC1-ser17 17Kda

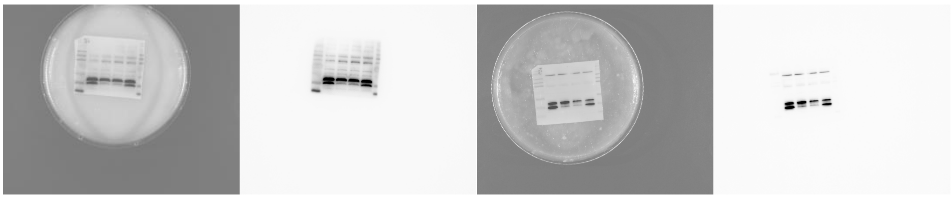

Figure 6A HCT 8

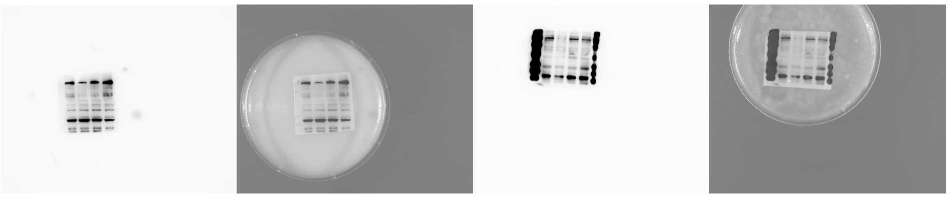

ULK1-Ser467 130Kda

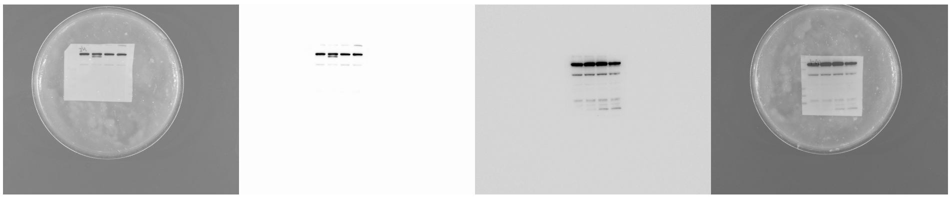

ULK1 130Kda

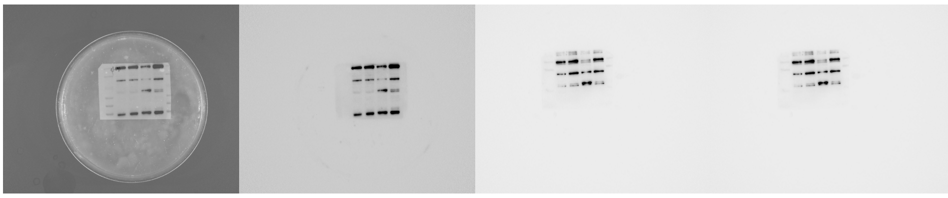

ULK1-Ser556 130Kda

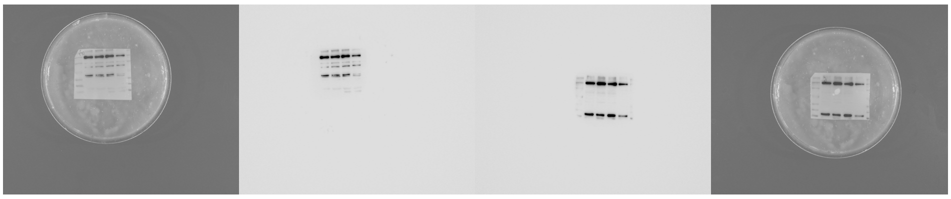

ULK1-Ser638 130Kda

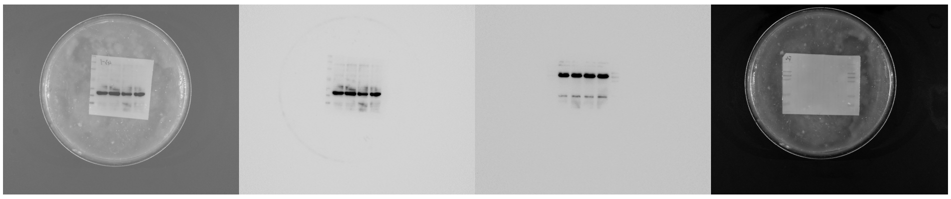

β-actin 42Kda

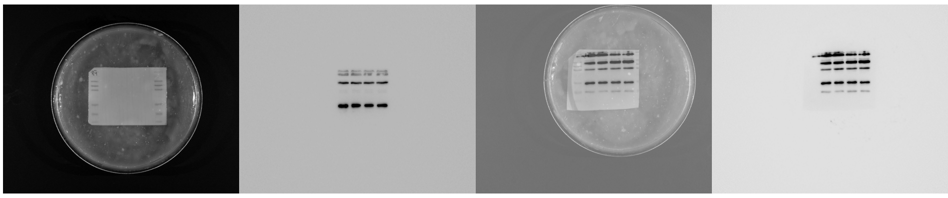

FUNDC1 17Kda

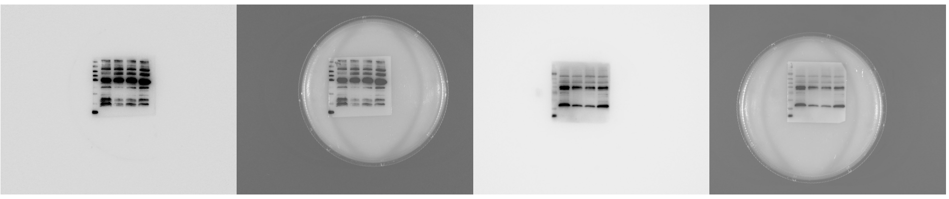

FUNDC1-Ser17 17Kda

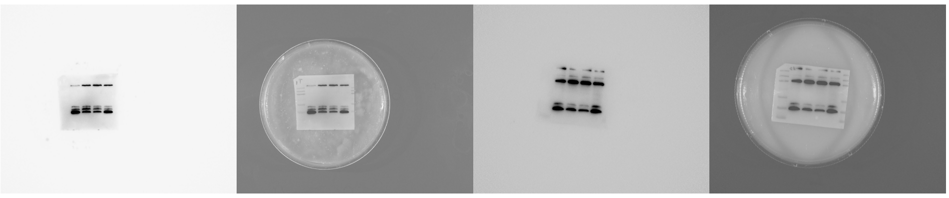

LC3 14/16Kda

Figure 6A HCT 116

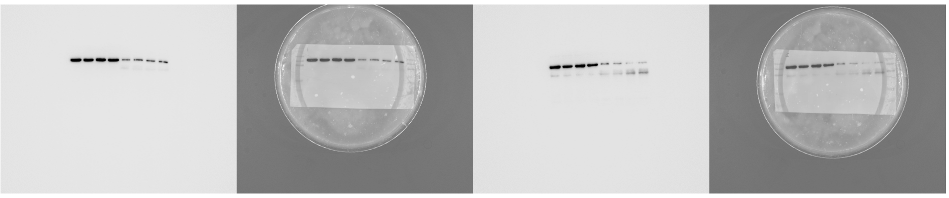

β-actin 42Kda

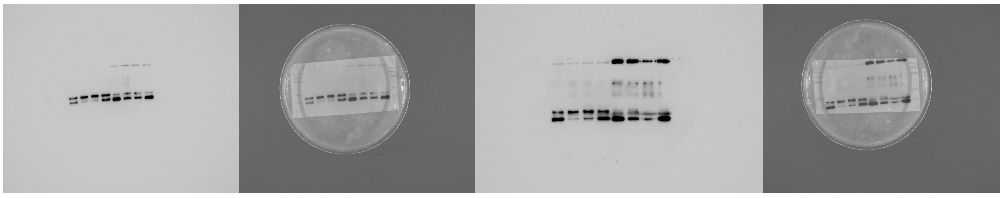

LC3 14/16Kda

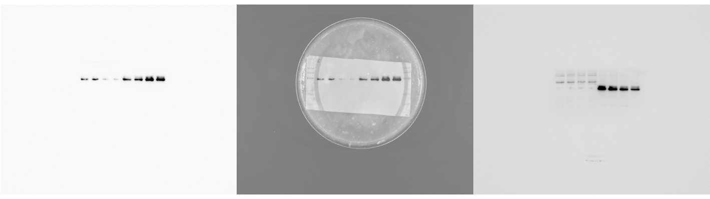

VDAC1 35Kda

Figure 6B

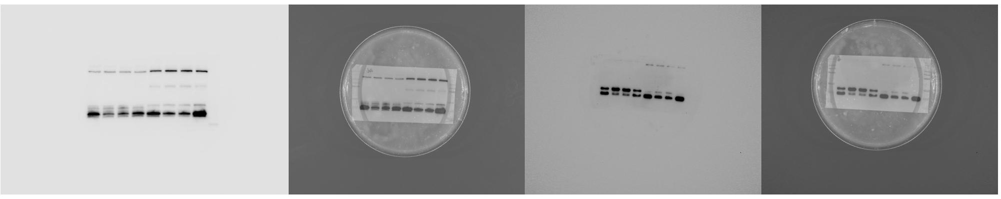

LC3 14/16Kda

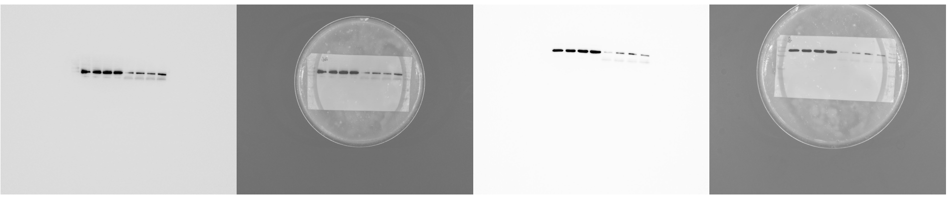

β-actin 42Kda

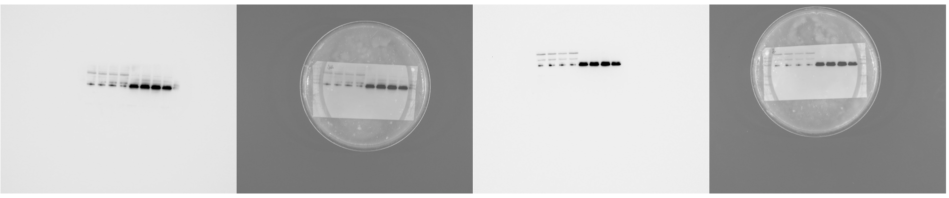

VDAC1 35Kda

Figure 6C

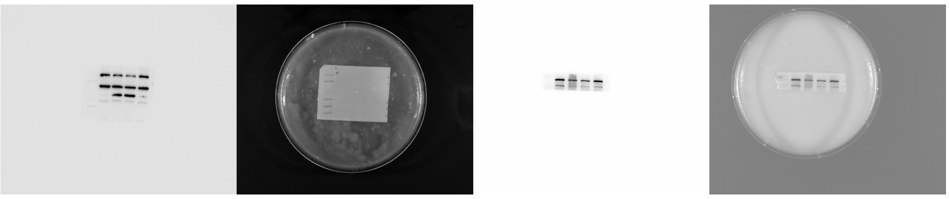

ULK1-Ser467 130Kda

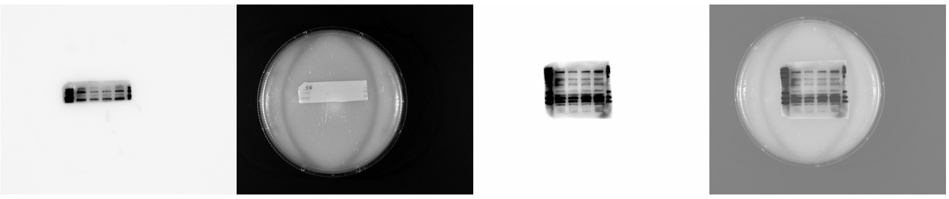

ULK1-Ser556 130Kda

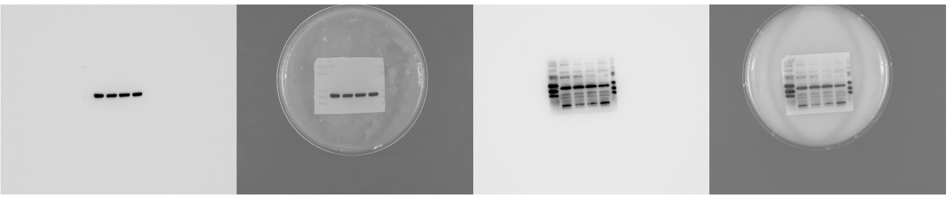

β-actin 42Kda

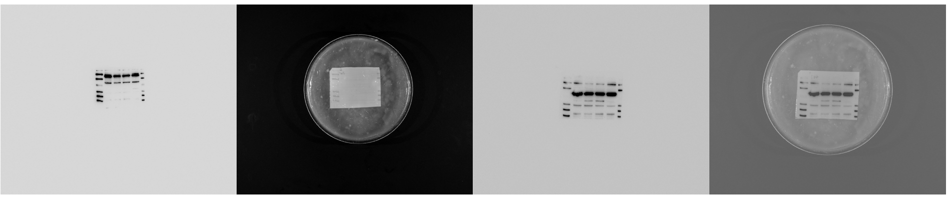

ULK1 130Kda

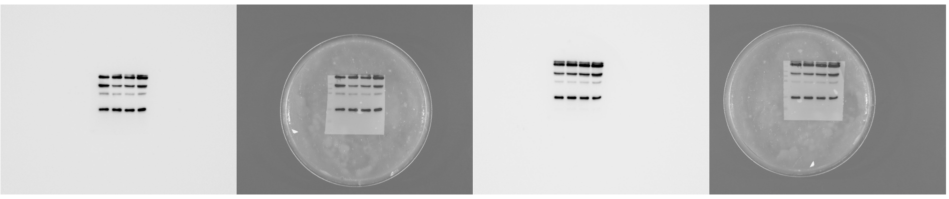

FUNDC1 17Kda

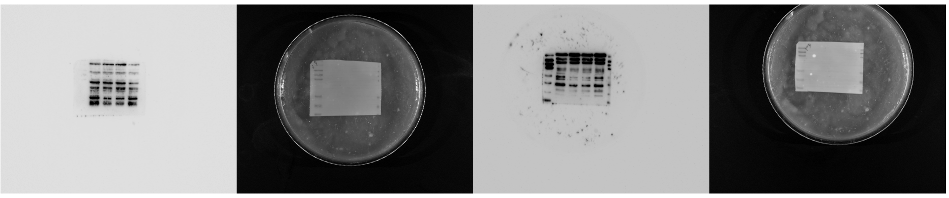

FUNDC1-Ser17   17Kda

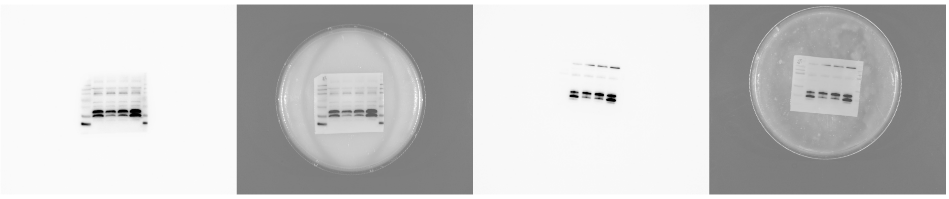

LC3   14/16Kda

Figure 7A   HCT8

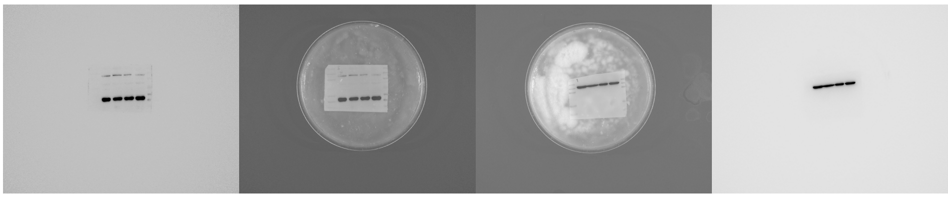

β-actin 42Kda

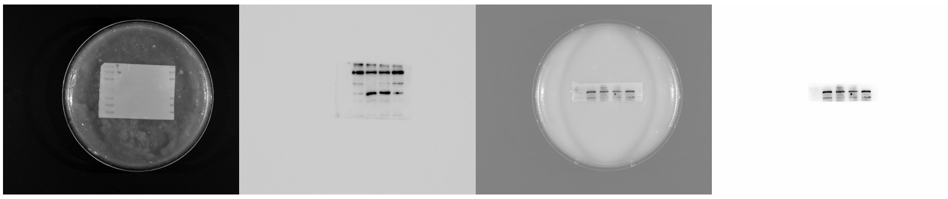

ULK1-Ser467 130Kda

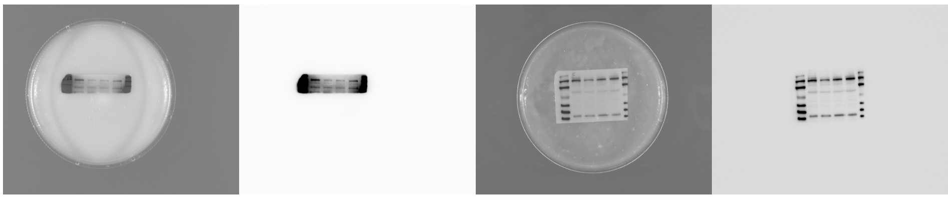

ULK1-Ser556 130Kda

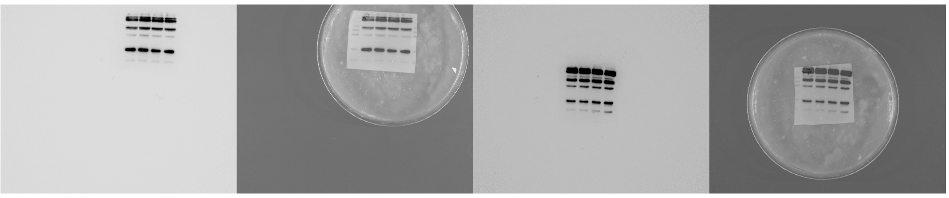

FUNDC1 17Kda

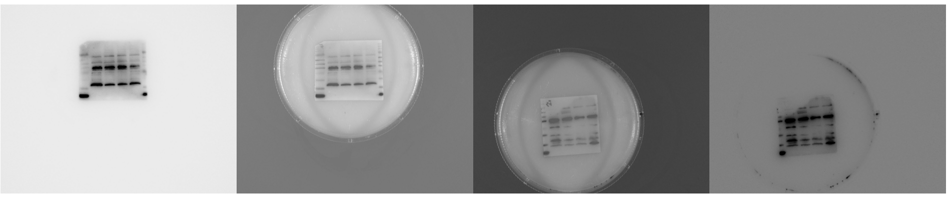

FUNDC1-Ser17 17Kda

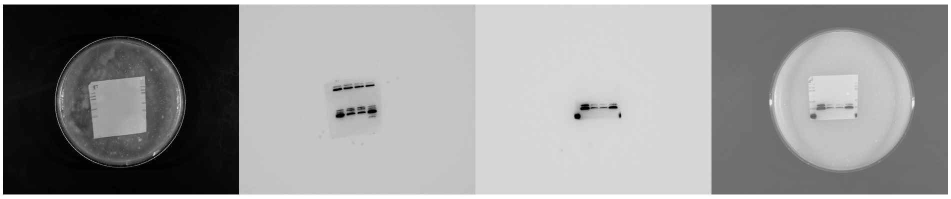

Figure 7A HCT116

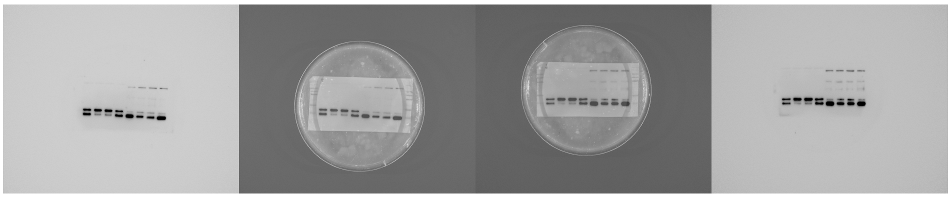

LC3 14/16Kda

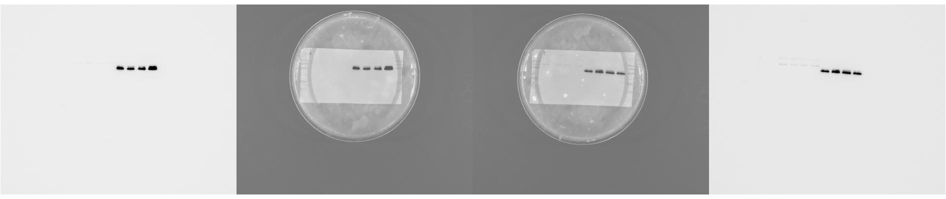

VDAC1 35Kda

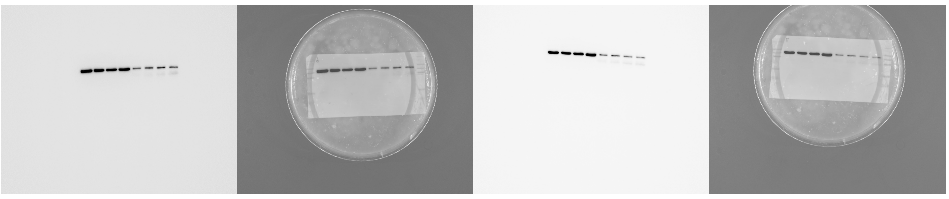

β-actin 42Kda

Figure 7B

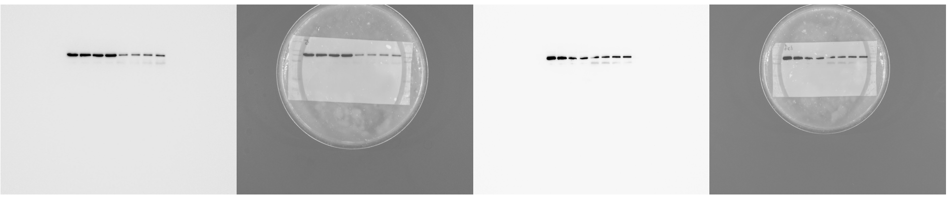

β-actin 42Kda

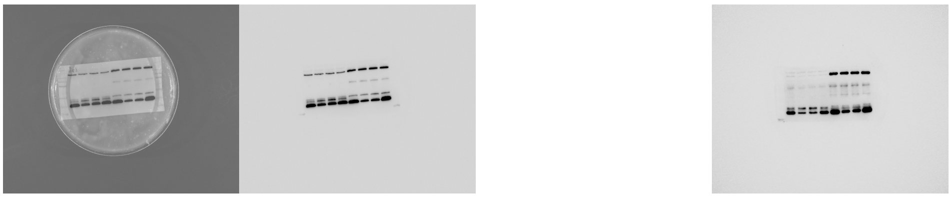

LC3 14/16Kda

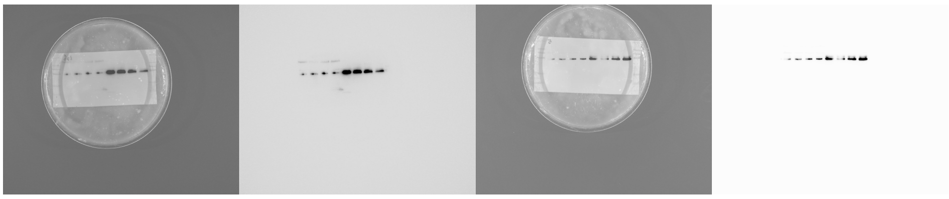

VDAC1 35Kda

Figure 7C

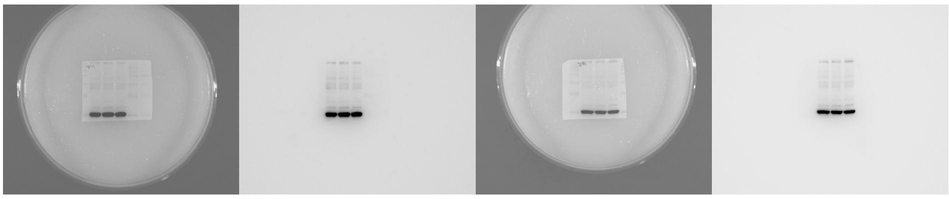

β-actin 42Kda

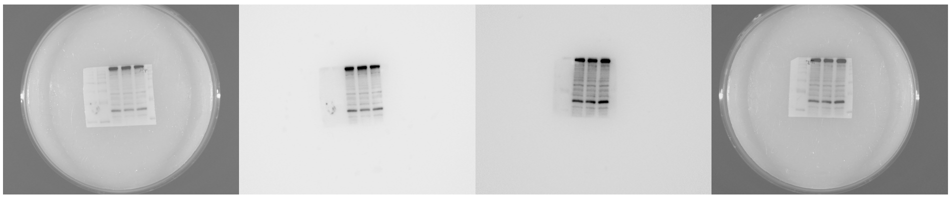

mTOR 289Kda

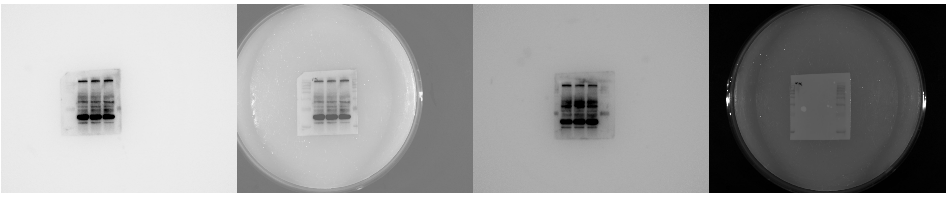

mTOR-Ser2448 289Kda

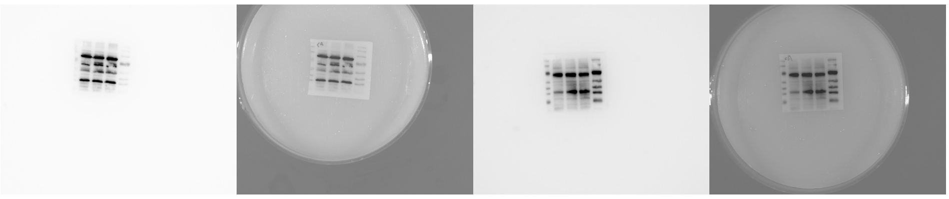

AMPK 62Kda

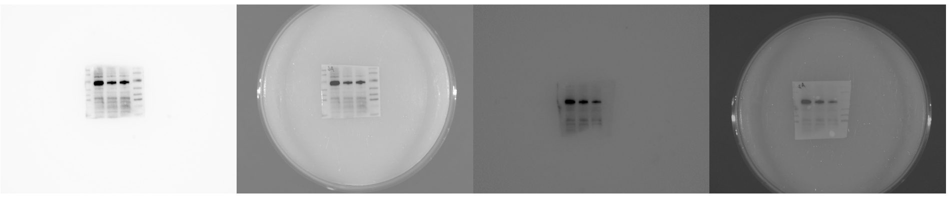

AMPK Thr172 64Kda

Figure 8A HCT8

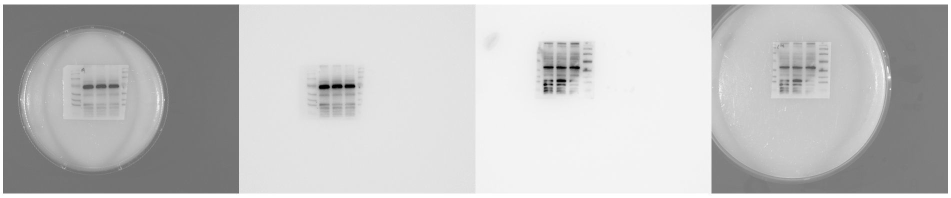

AMPK 62Kda

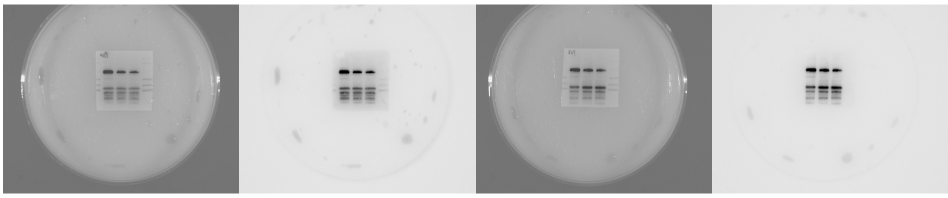

AMPK Thr172 64Kda

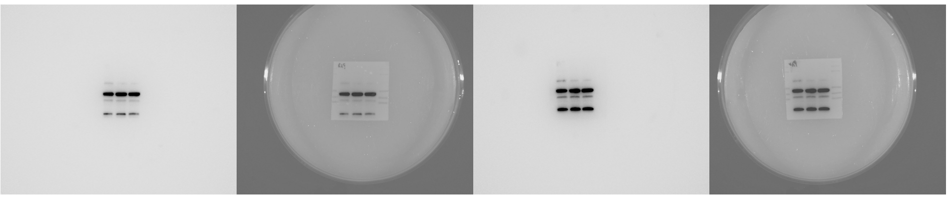

β-actin 42Kda

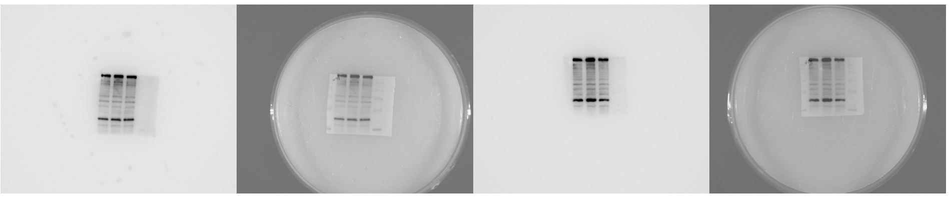

mTOR 289Kda

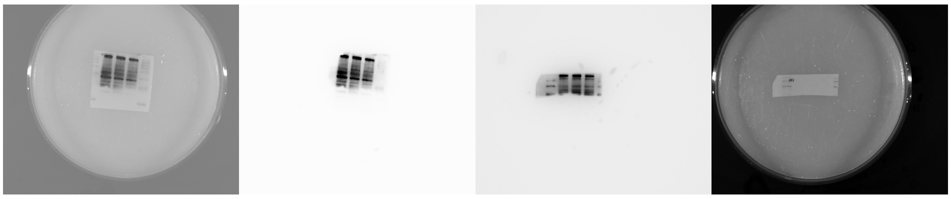

mTOR-ser 2448 289Kda

Figure 8A HCT116

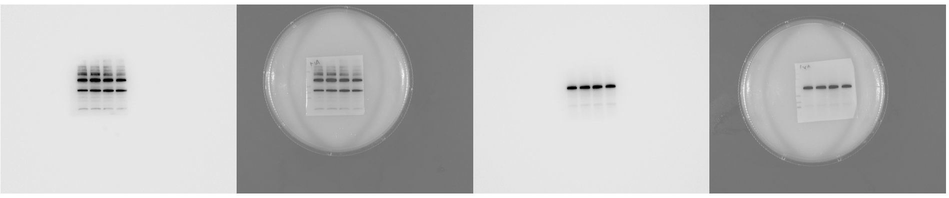

AMPK 62Kda

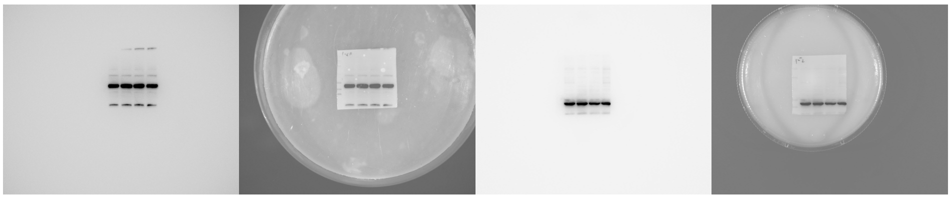

β-actin 42Kda

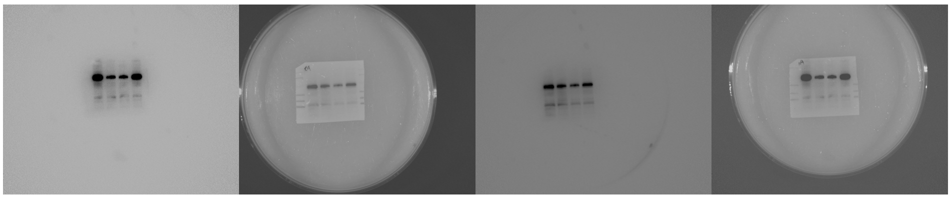

AMPK Thr 172 64Kda

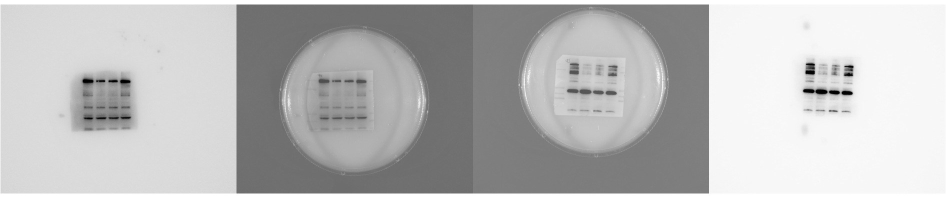

ULK1-Ser467 130Kda

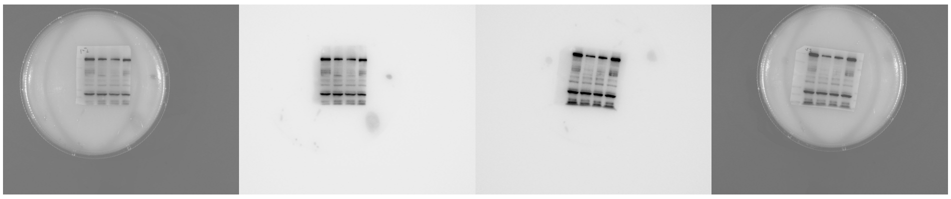

ULK1-Ser556 130Kda

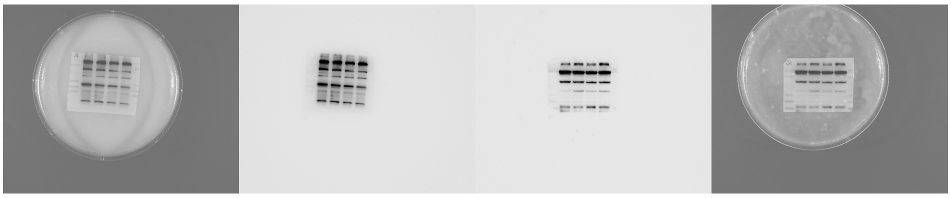

ULK1 130Kda

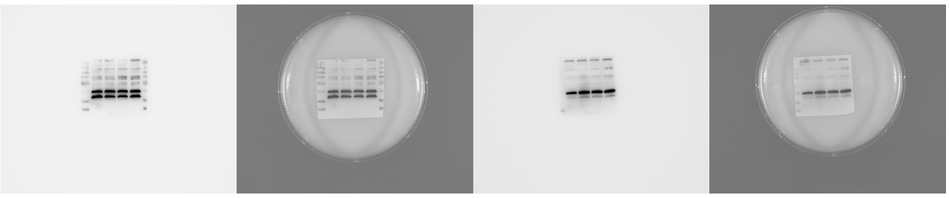

FUNDC1 17Kda

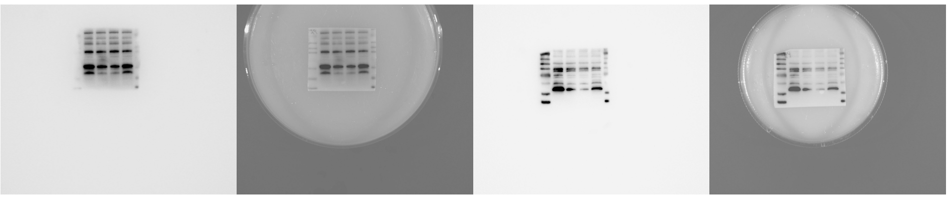

FUNDC1-Ser17 17Kda

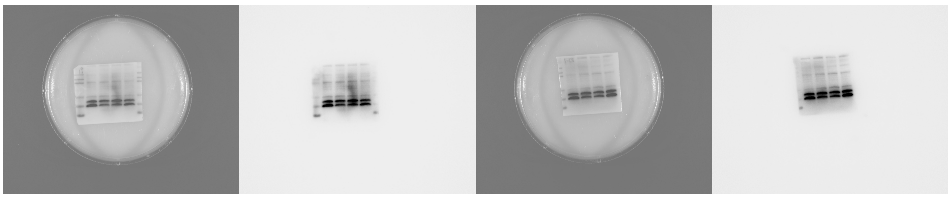

LC3 14/16Kda

Figure 8C HCT8

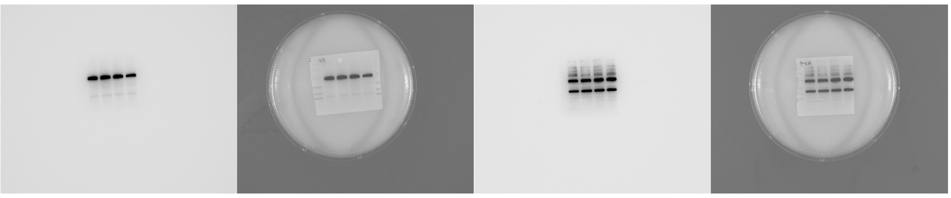

AMPK 62Kda

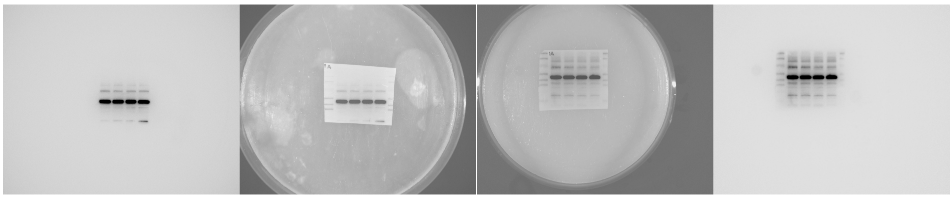

β-actin 42Kda

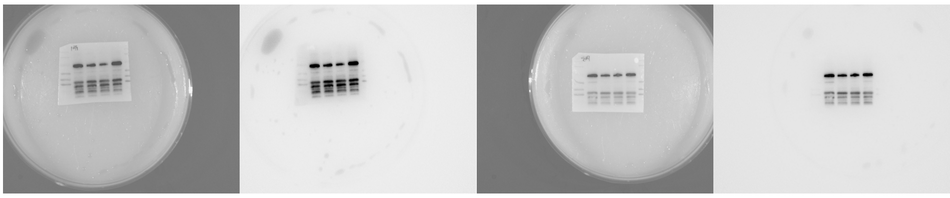

AMPK Thr 172 64Kda

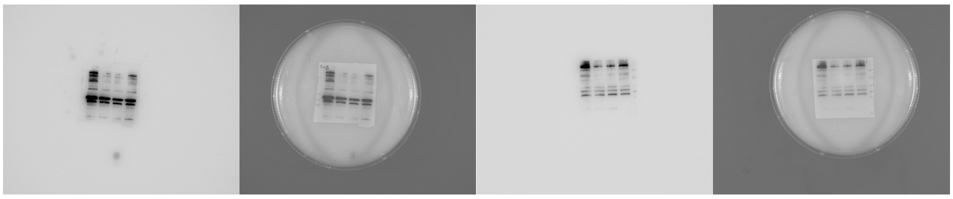

ULK1 Ser-467 130Kda

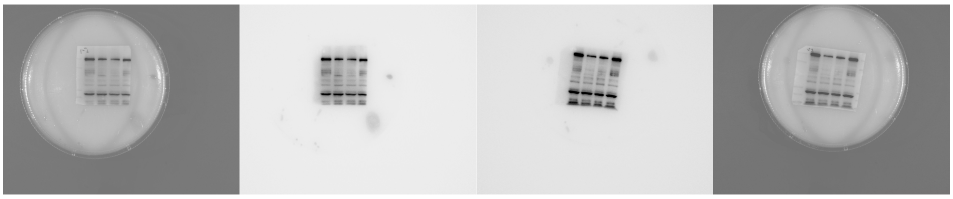

ULK1 Ser-556 130Kda、

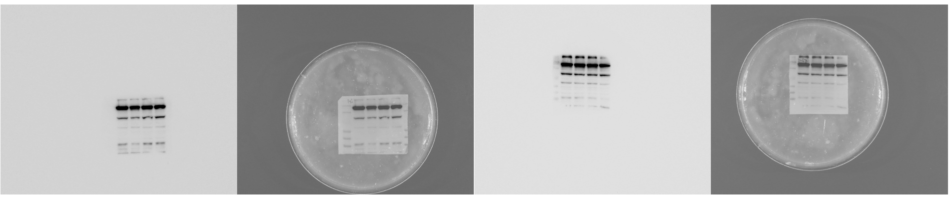

ULK1 130Kda

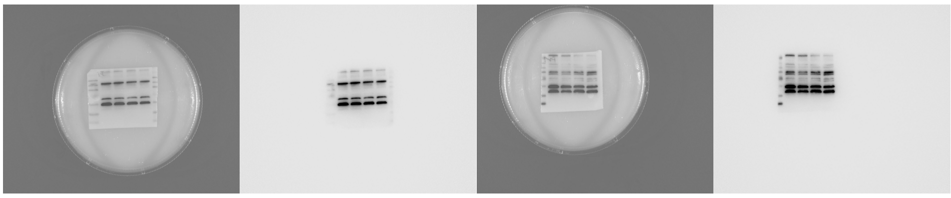

FUNDC1 17Kda

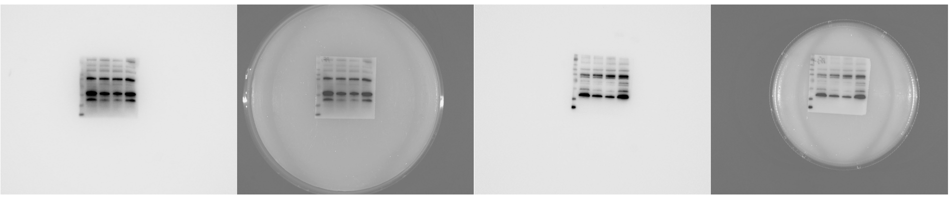

FUNDC1-Ser17 17Kda

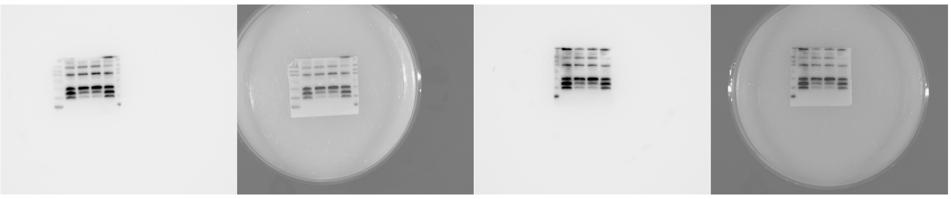

Figure 8C HCT 116

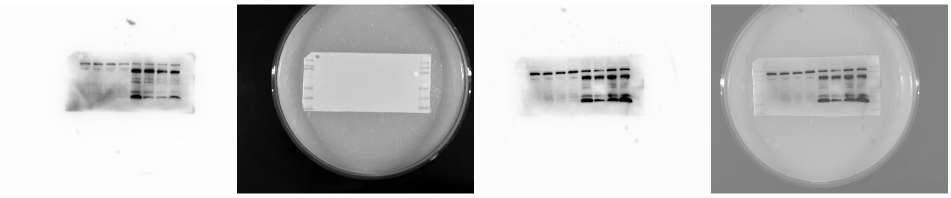

VDAC1 35Kda

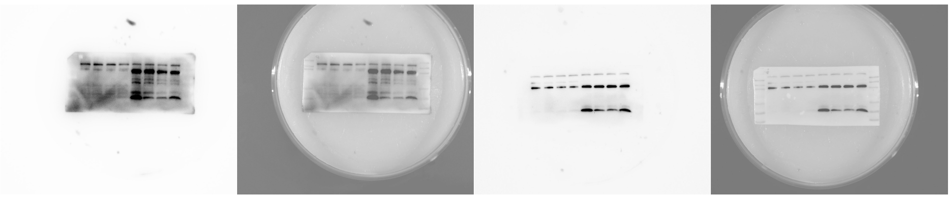

LC3 14/16Kda

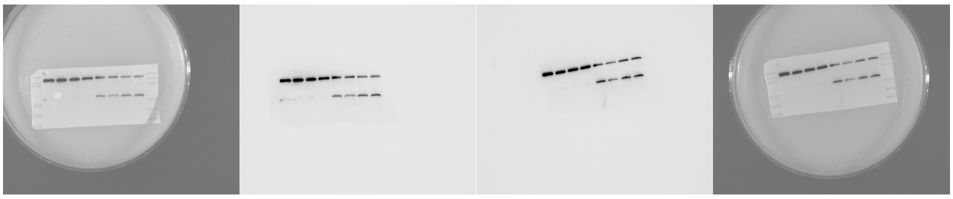

β-actin 42Kda

Figure 8D HCT8

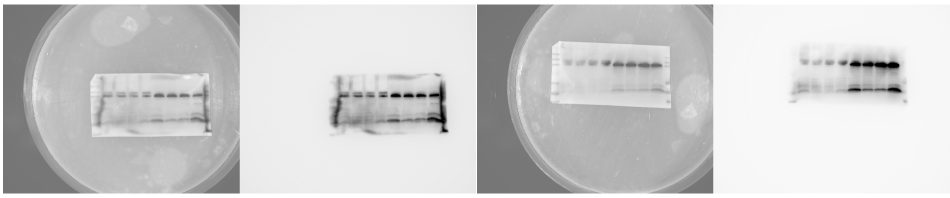

VDAC1 35Kda

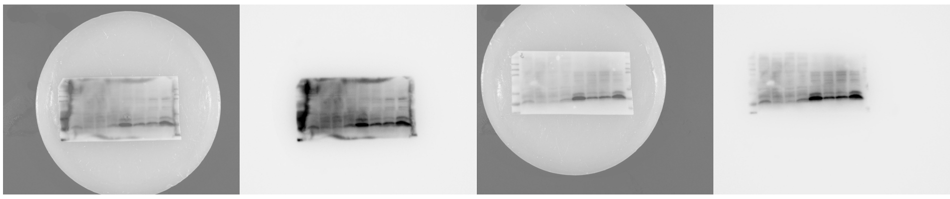

LC3 14/16Kda

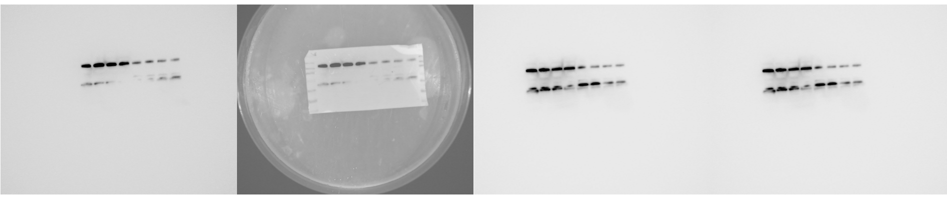

β-actin 42Kda

Figure 8D HCT116

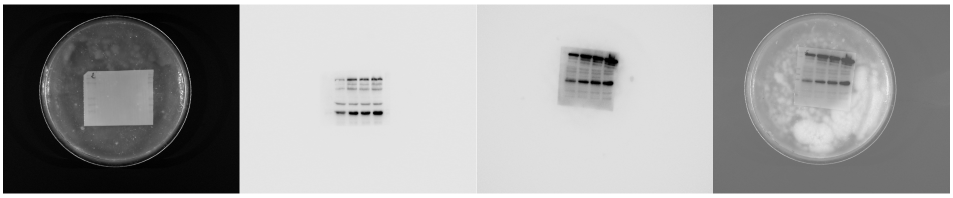

PPA1 33Kda

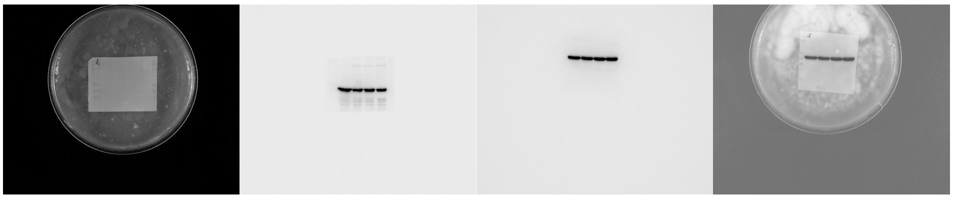

β-actin 42Kda

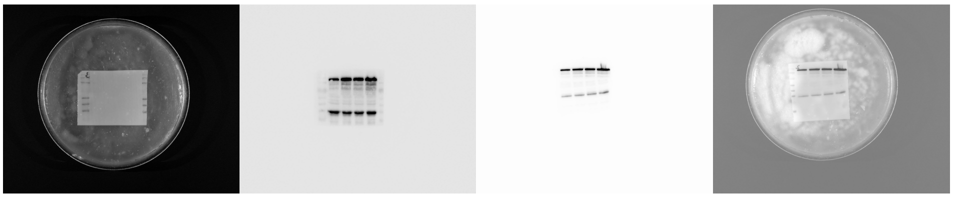

N-CA 130Kda

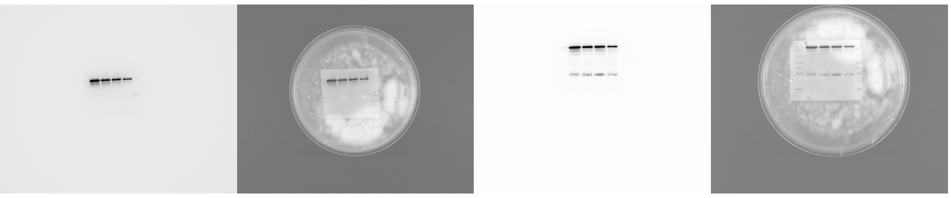

E-CA 130Kda

Figure 9D

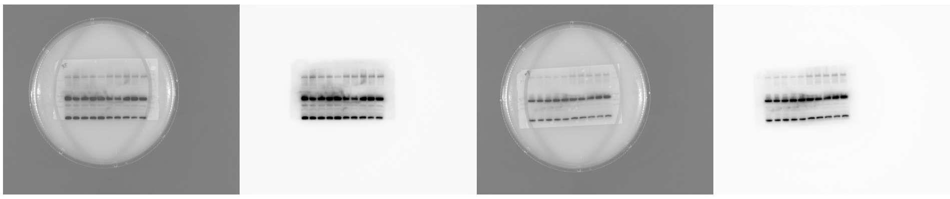

β-actin 42Kda

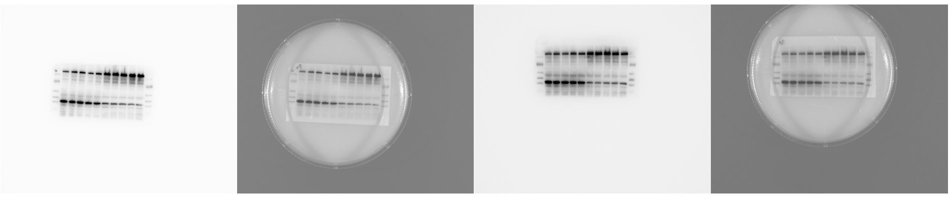

E-CA 130Kda

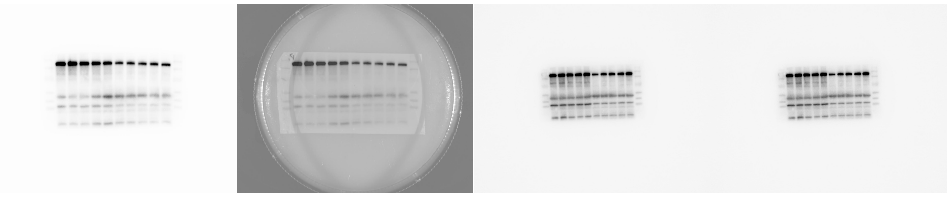

N-CA 130Kda

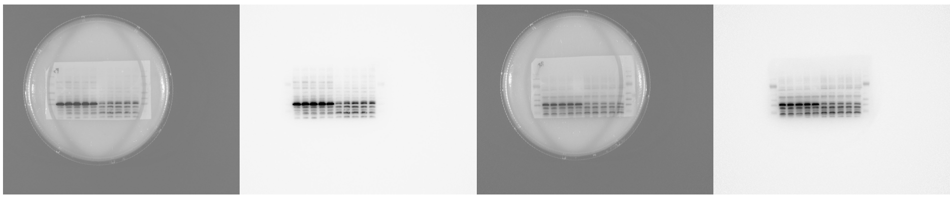

PPA1 33Kda

Figure 9H
