# Supplementary figures and images for: PPA1 promotes oxidative phosphorylation and malignant progression of colorectal cancer under glucose restriction via AMPK/ULK1/FUNDC1-mediated mitophagy
Source: Cell Death Discov. 2025 Nov 28;11:549. doi: 10.1038/s41420-025-02816-y (PMC12663196; doi:10.1038/s41420-025-02816-y)

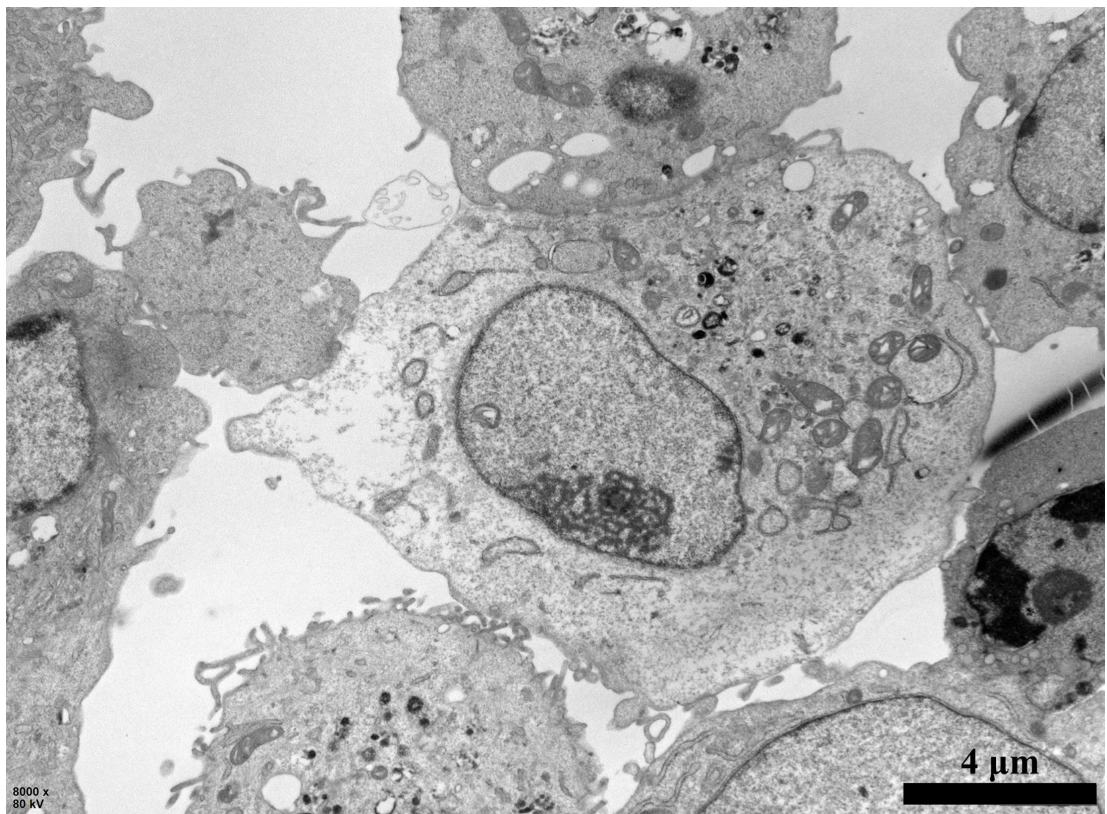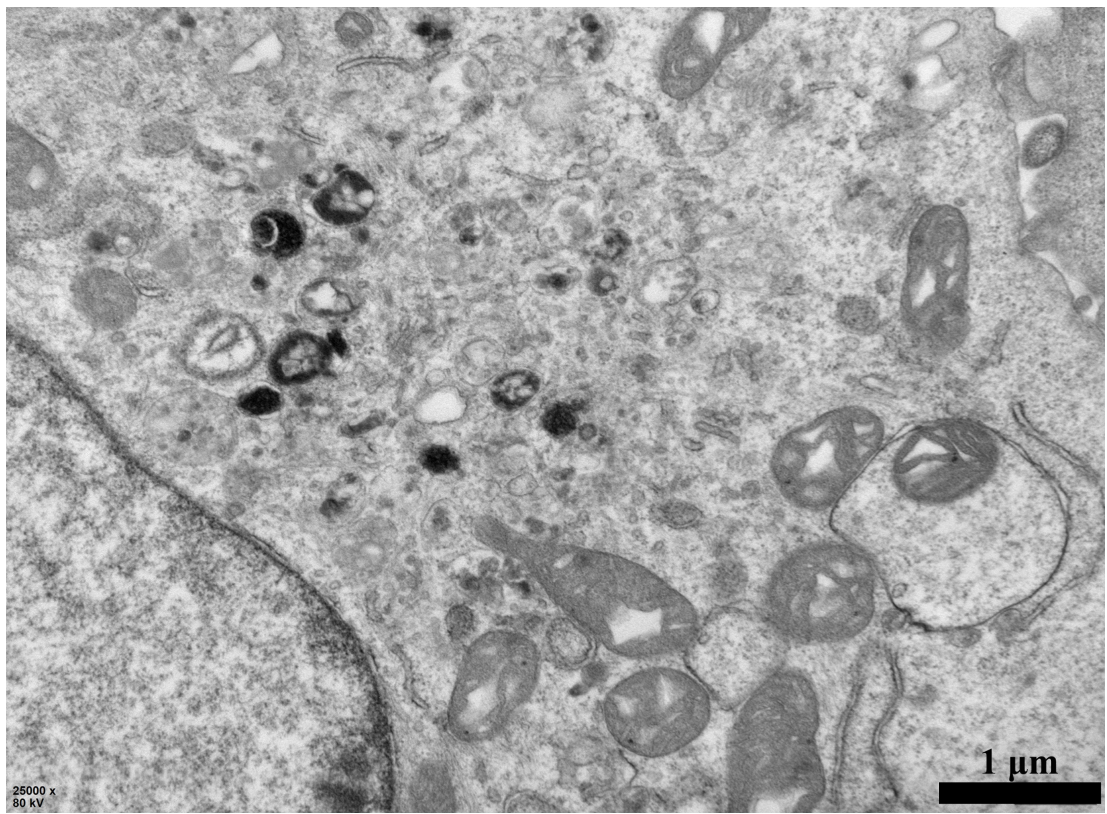

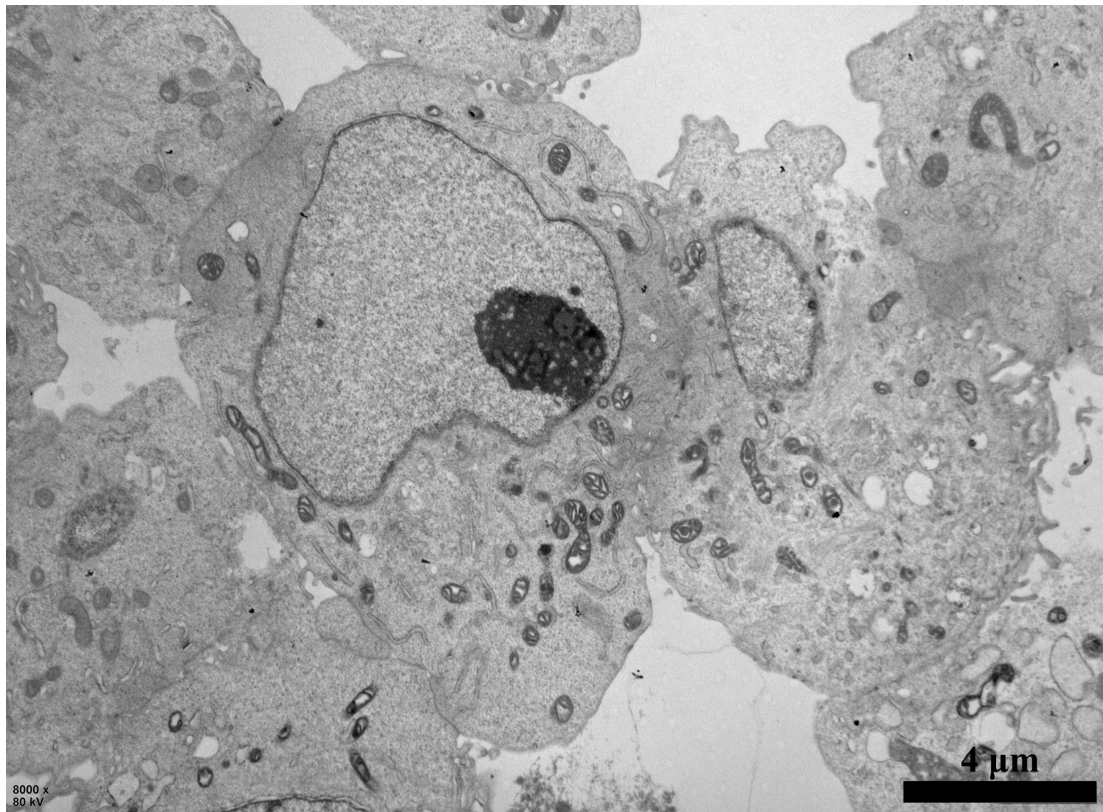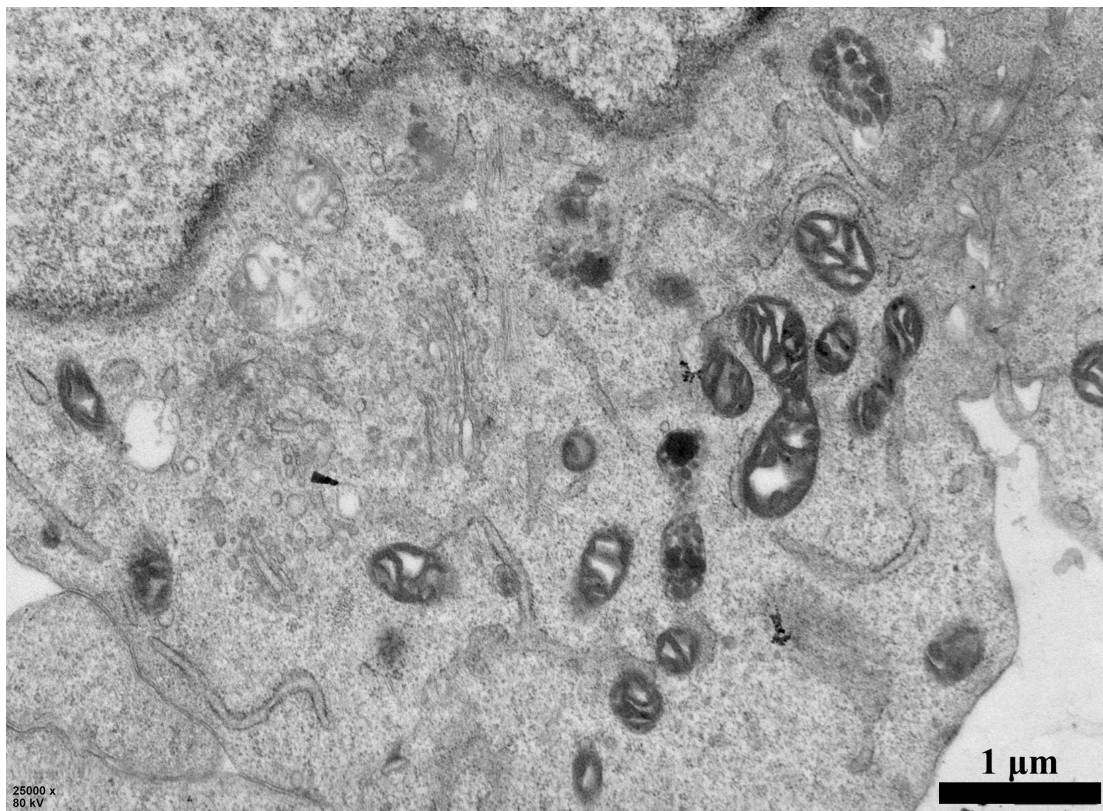

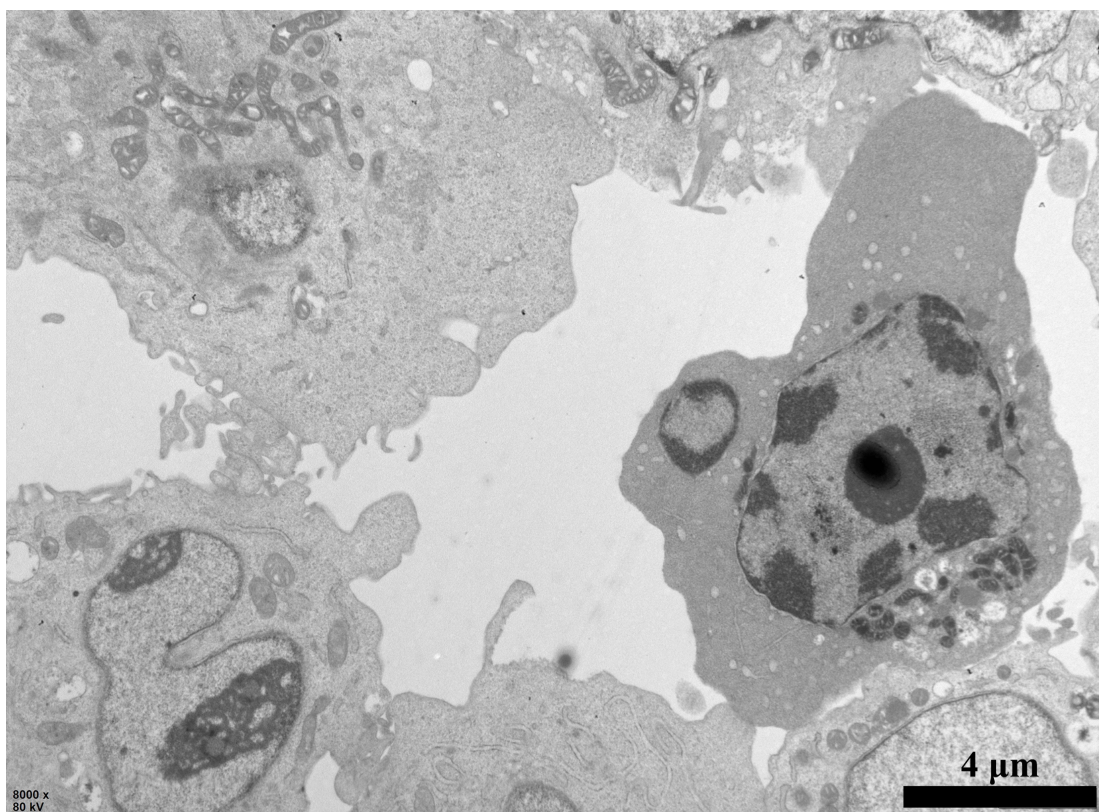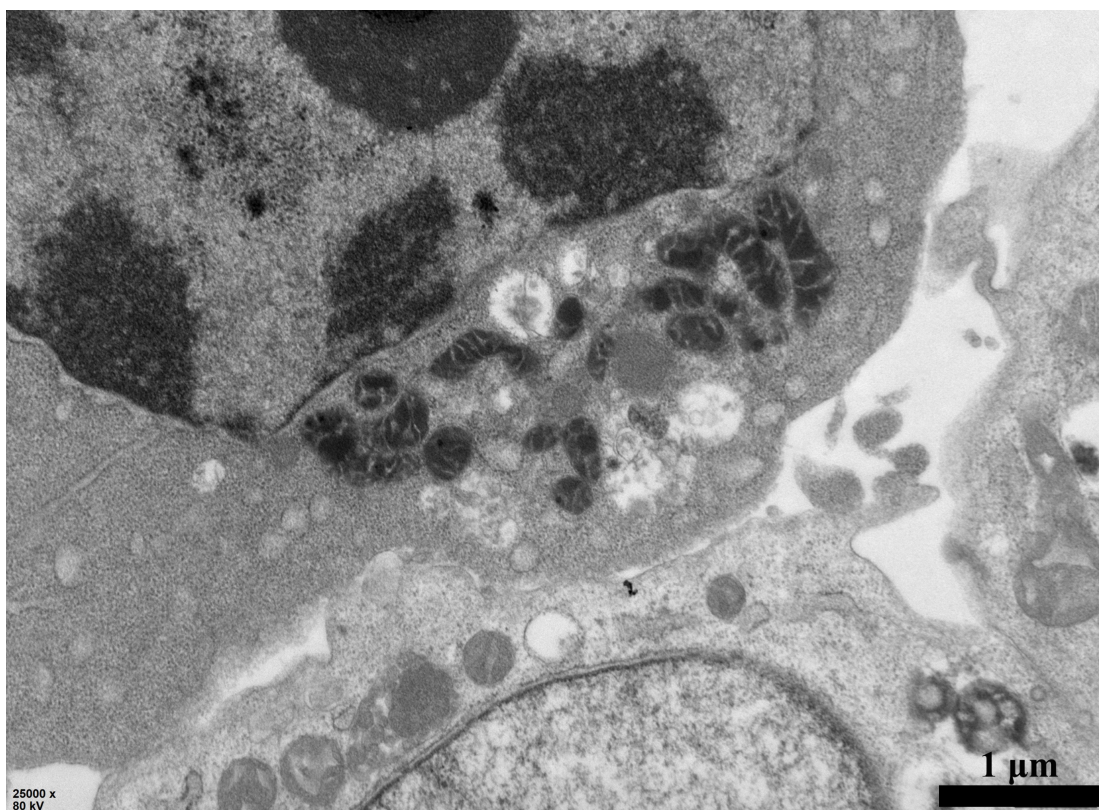

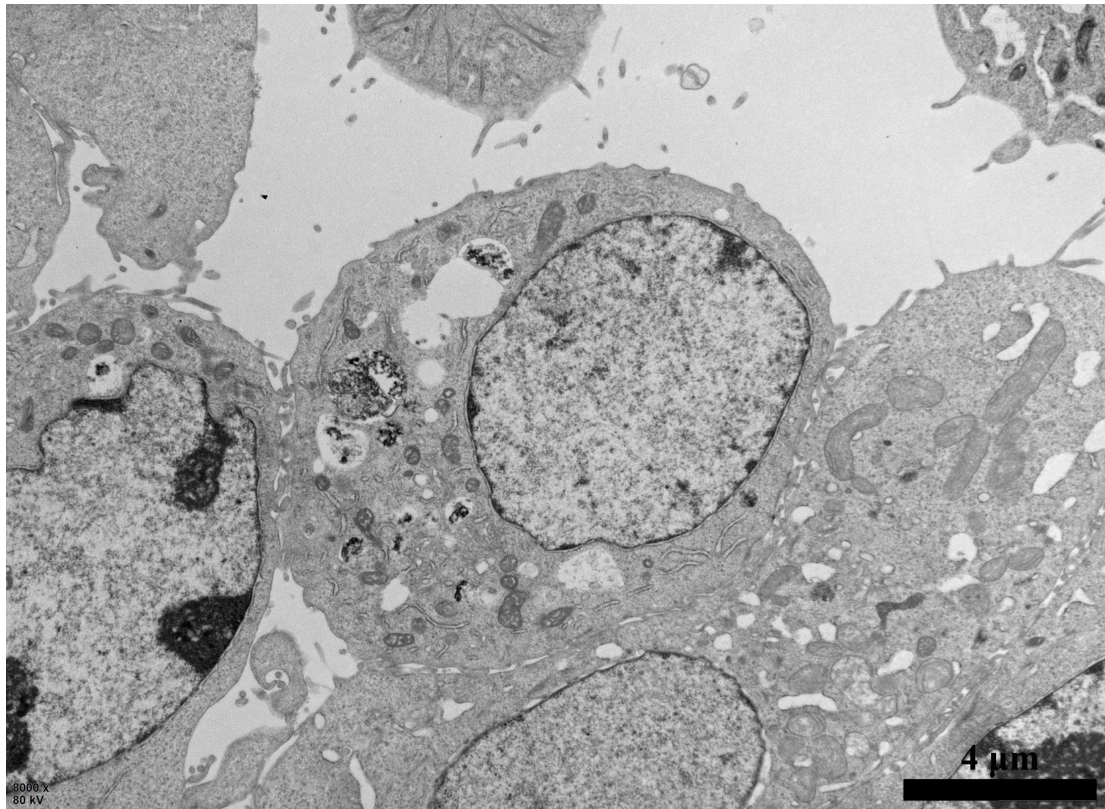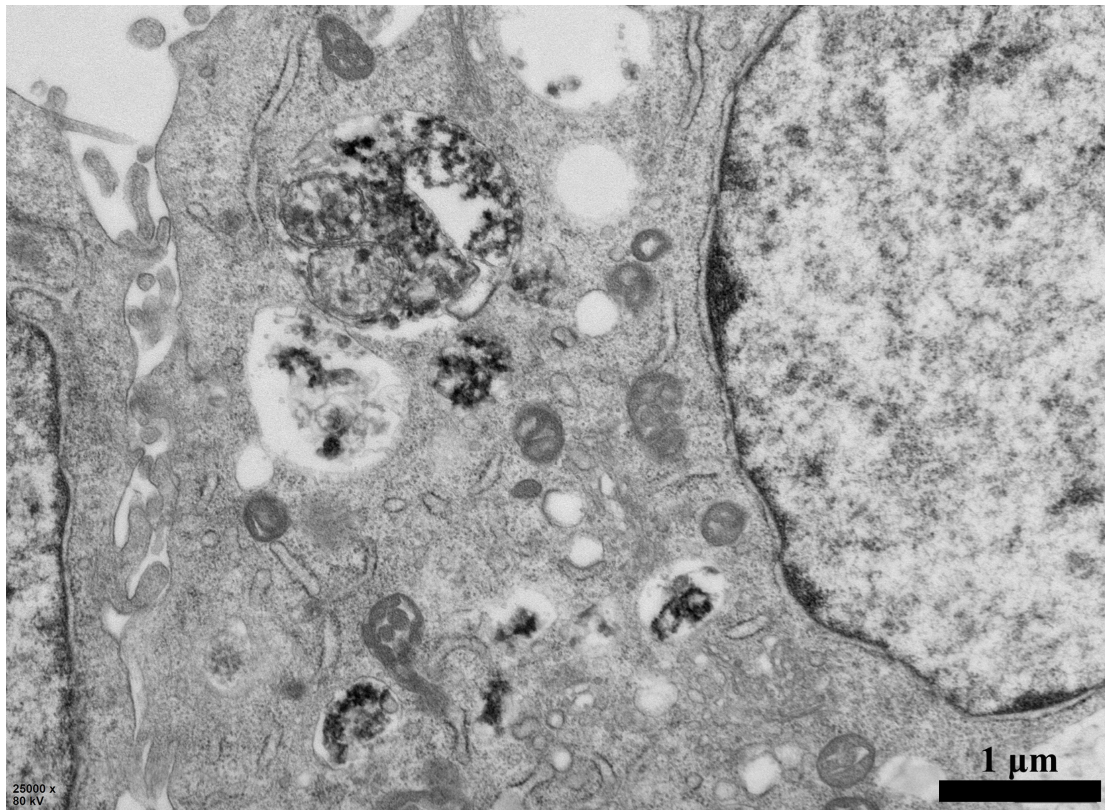

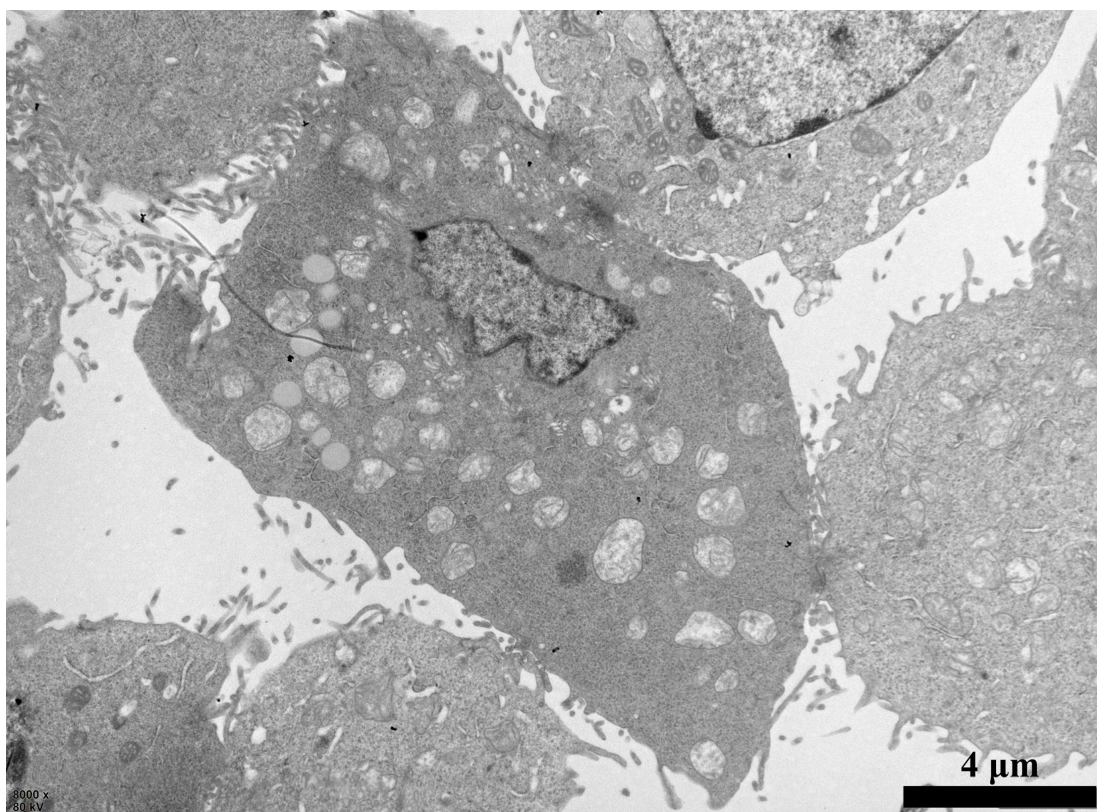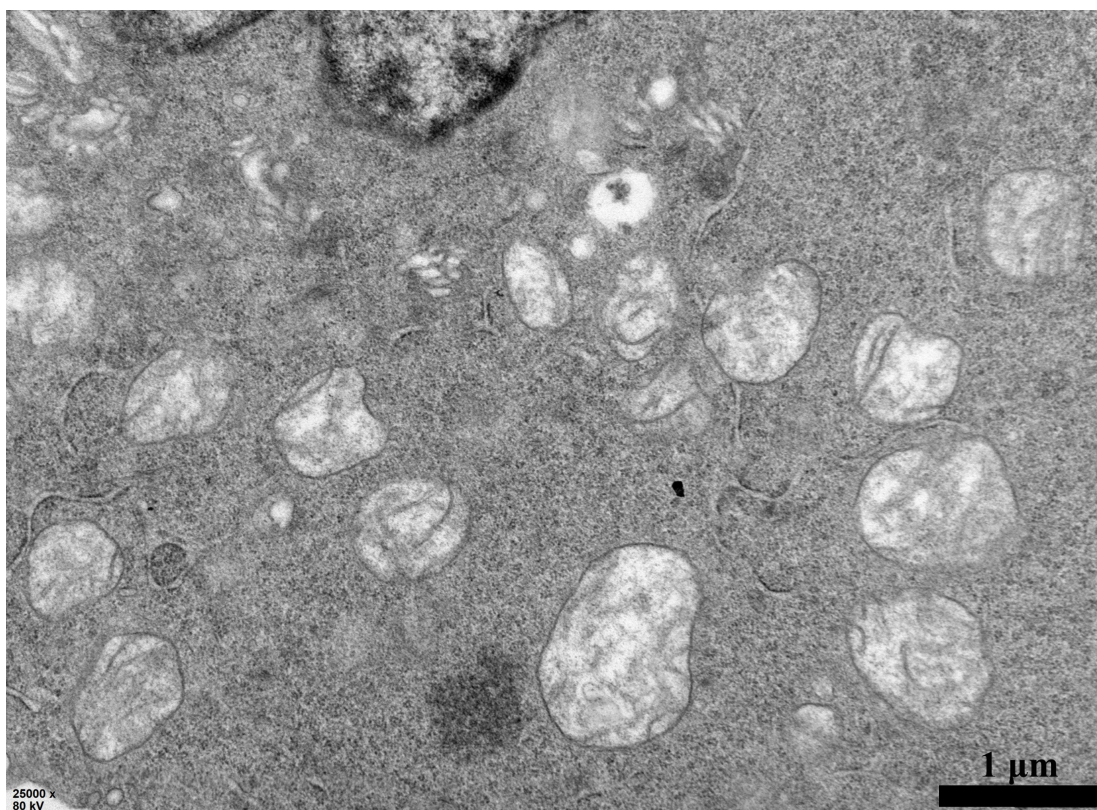

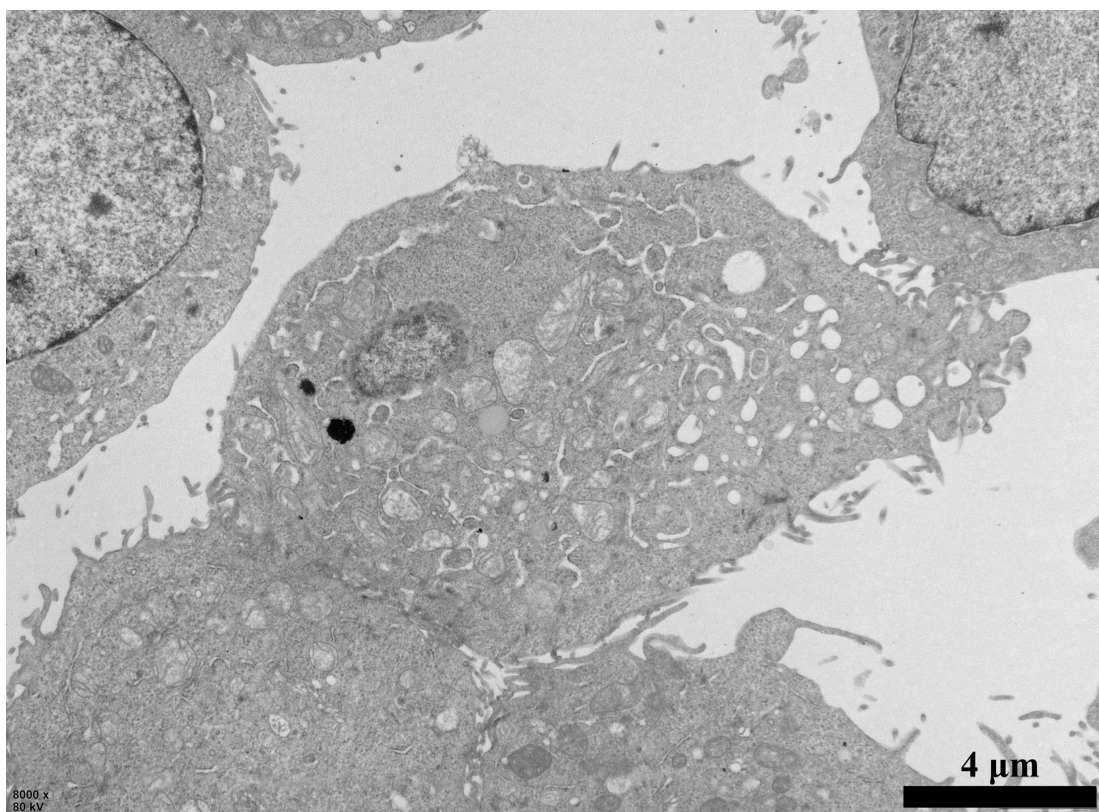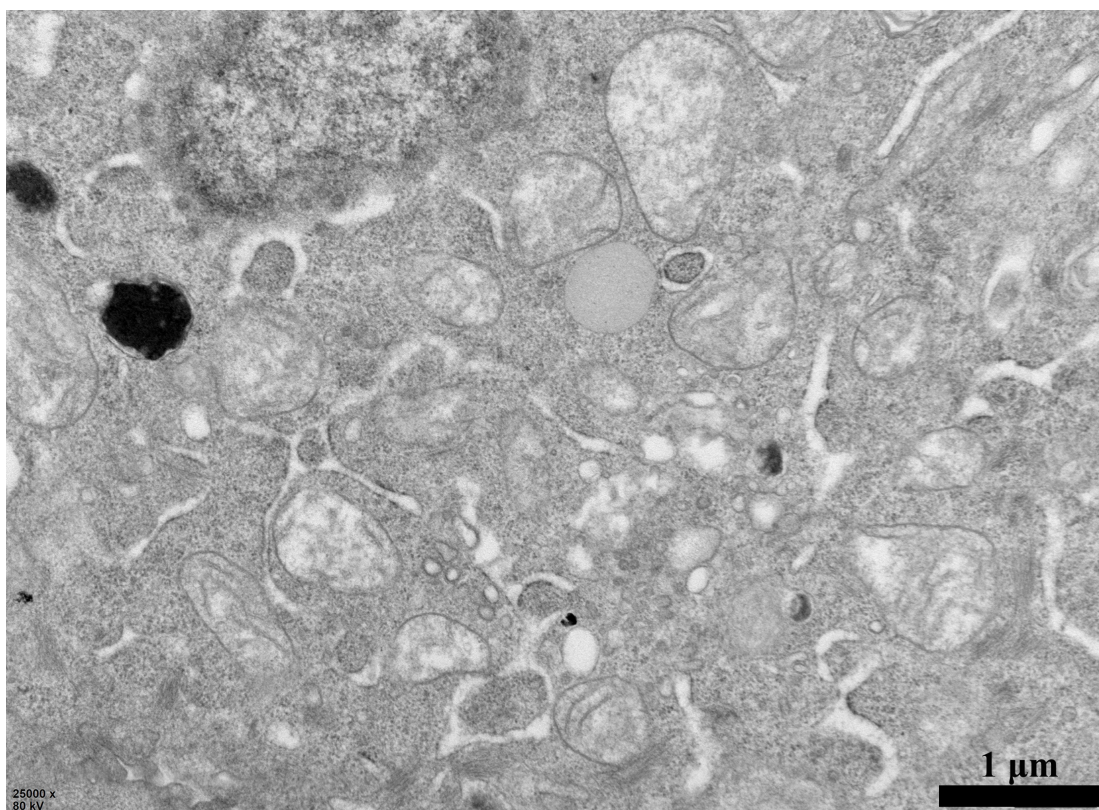

Supplement: Supplementary file 3 — The TME image [file 41420_2025_2816_MOESM3_ESM.pdf]

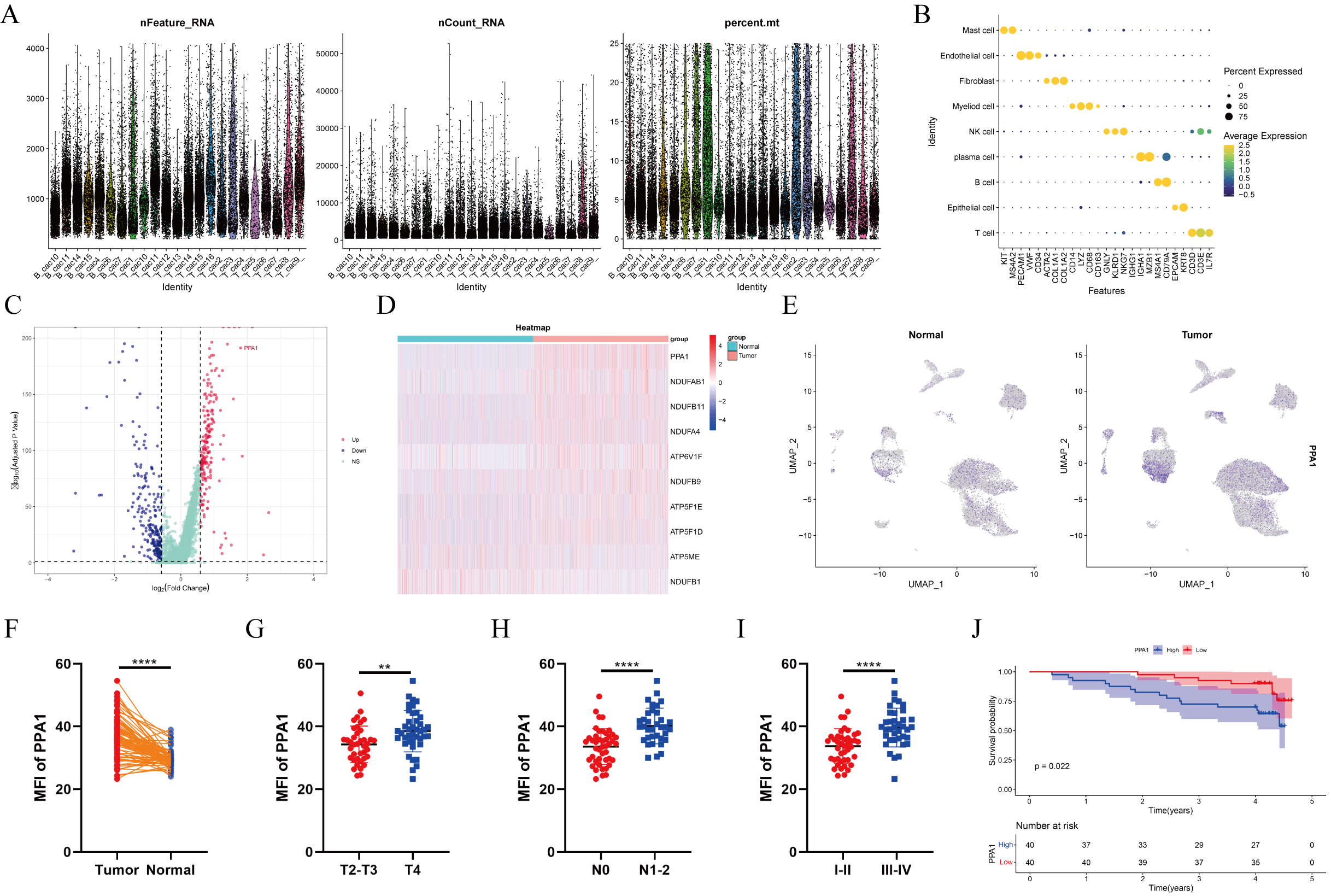

Supplement: Supplementary file 4 — Figure S1 [file 41420_2025_2816_MOESM4_ESM.tif]

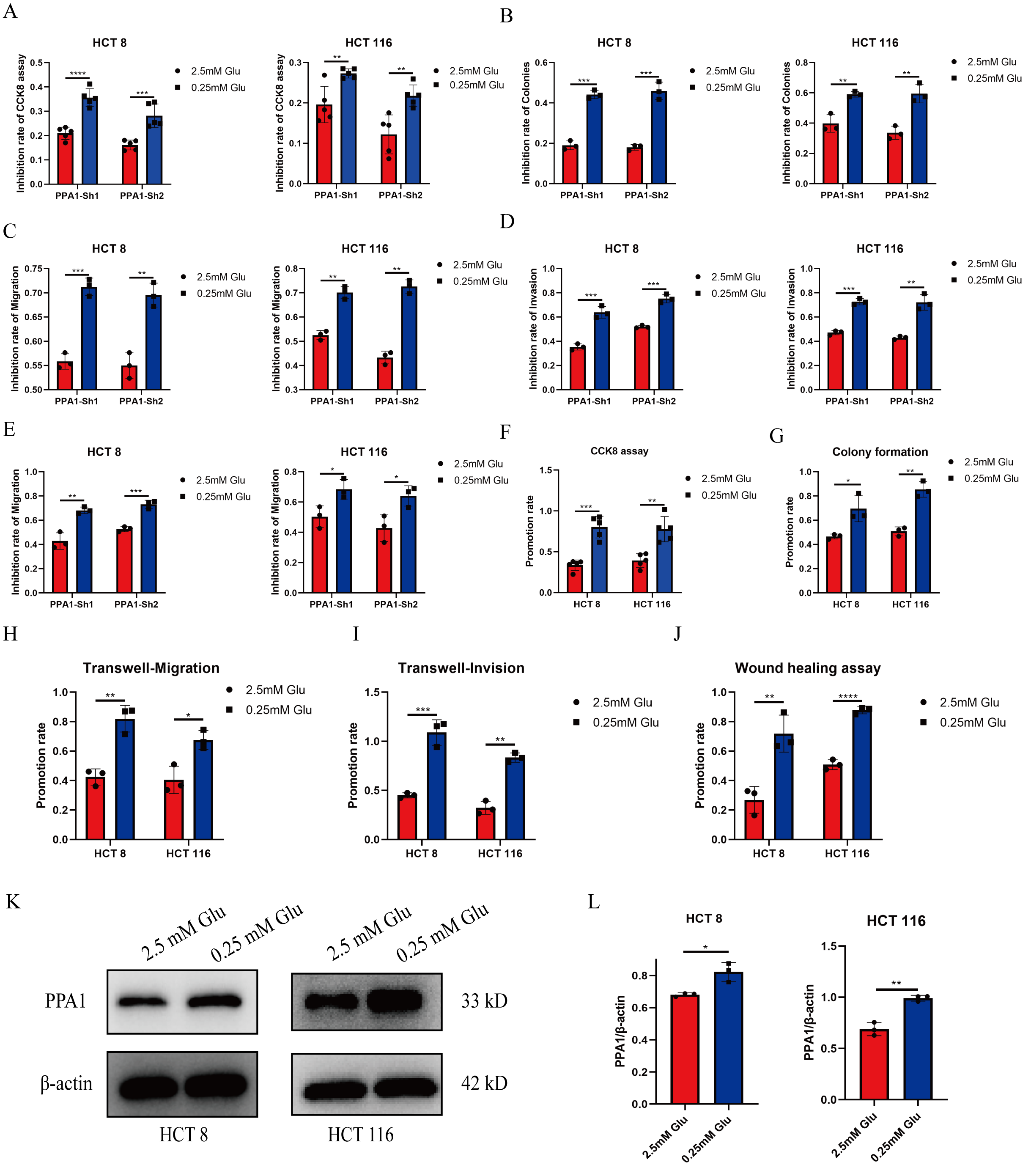

Supplement: Supplementary file 5 — Figure S2 [file 41420_2025_2816_MOESM5_ESM.tif]

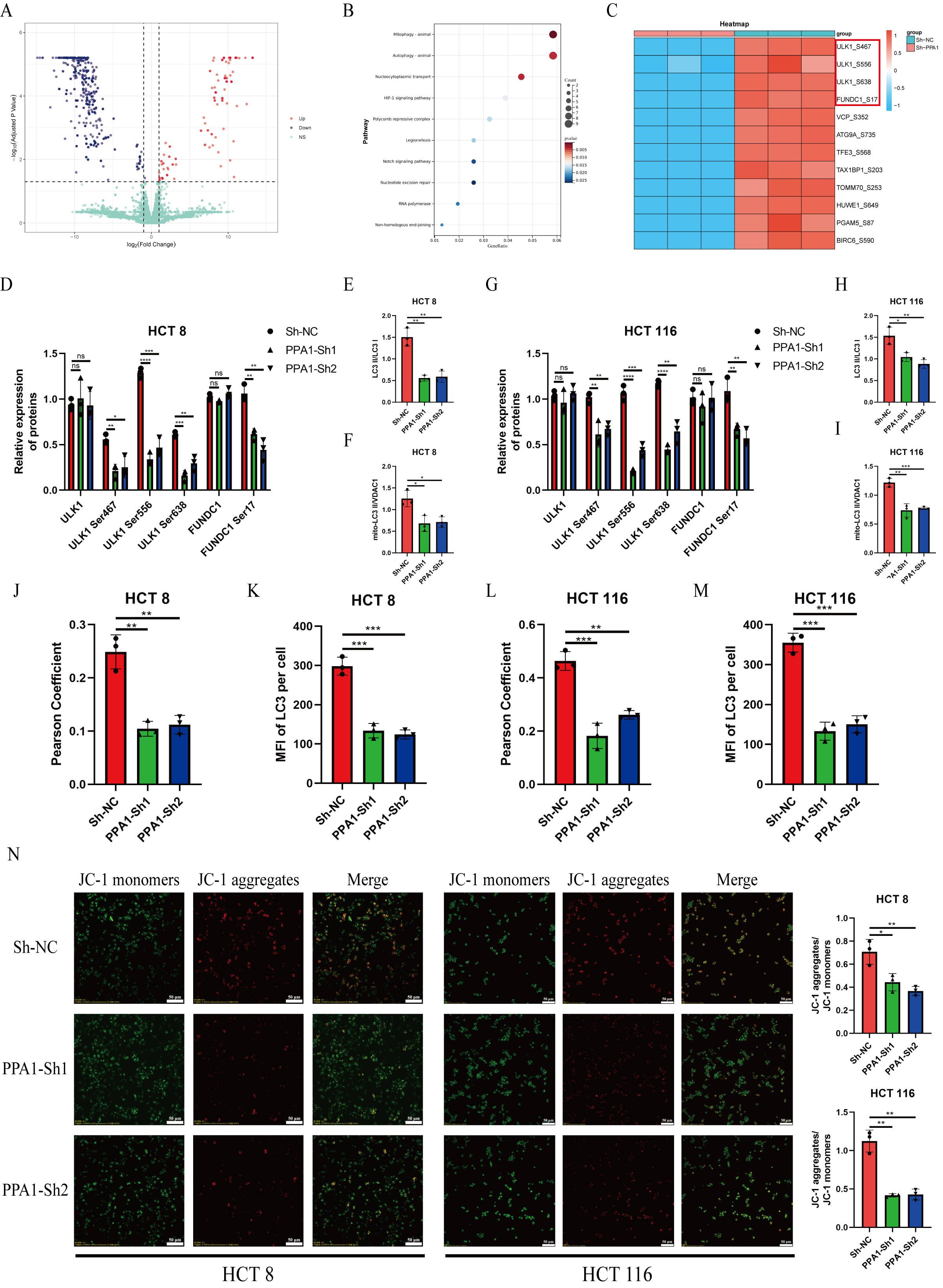

Supplement: Supplementary file 6 — Figure S3 [file 41420_2025_2816_MOESM6_ESM.tif]

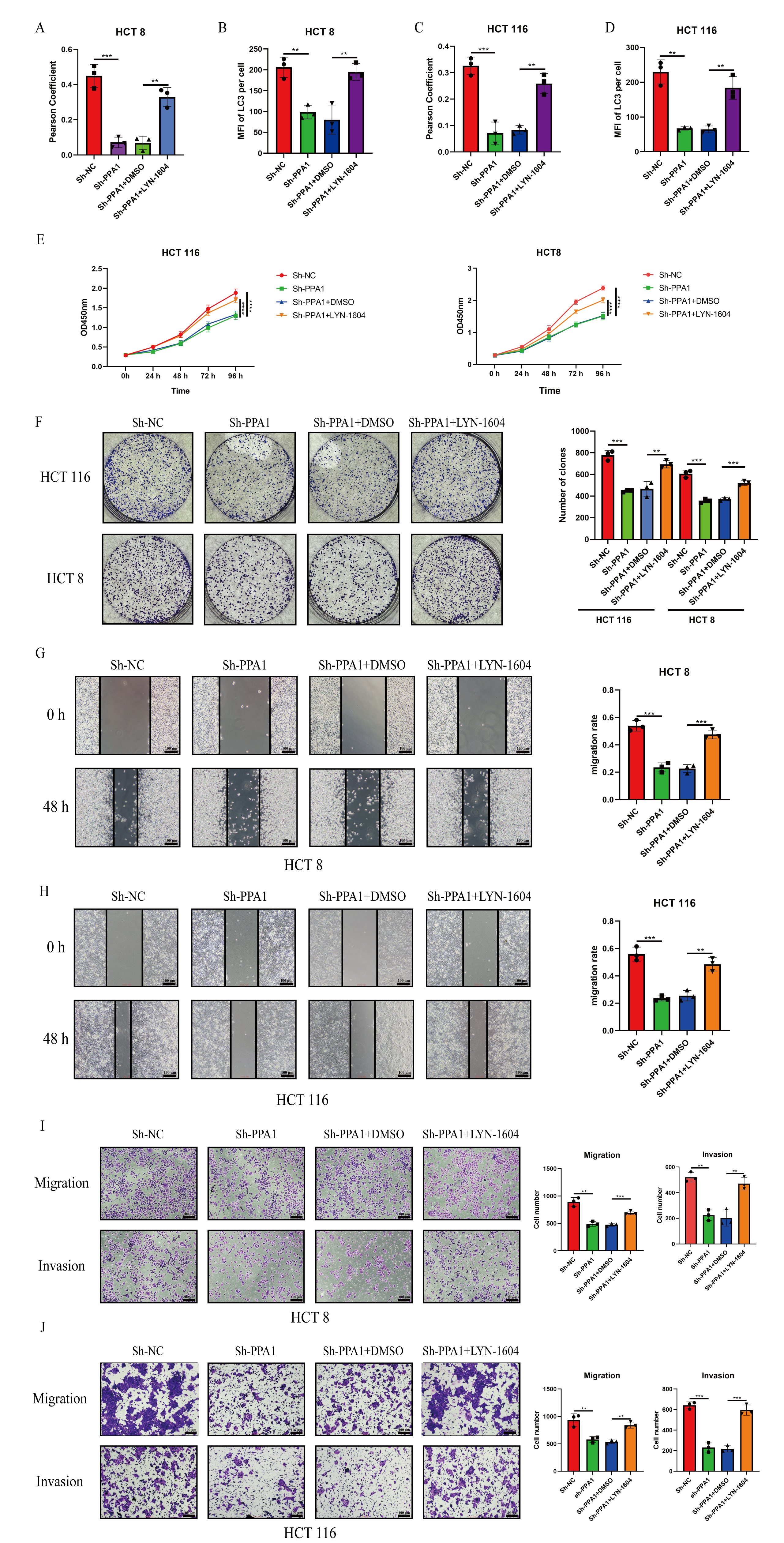

Supplement: Supplementary file 7 — Figure S4 [file 41420_2025_2816_MOESM7_ESM.tif]

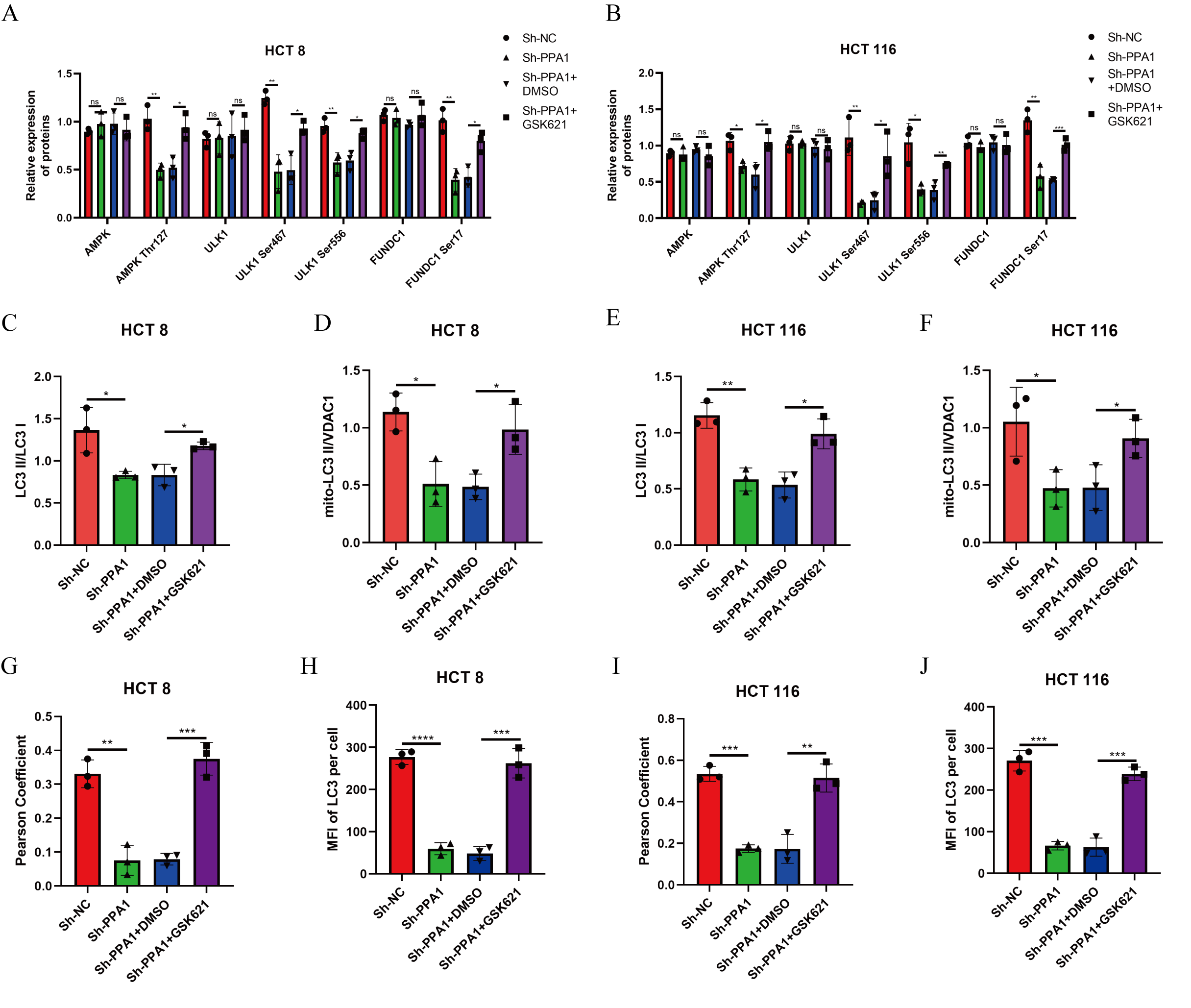

Supplement: Supplementary file 8 — Figure S5 [file 41420_2025_2816_MOESM8_ESM.tif]

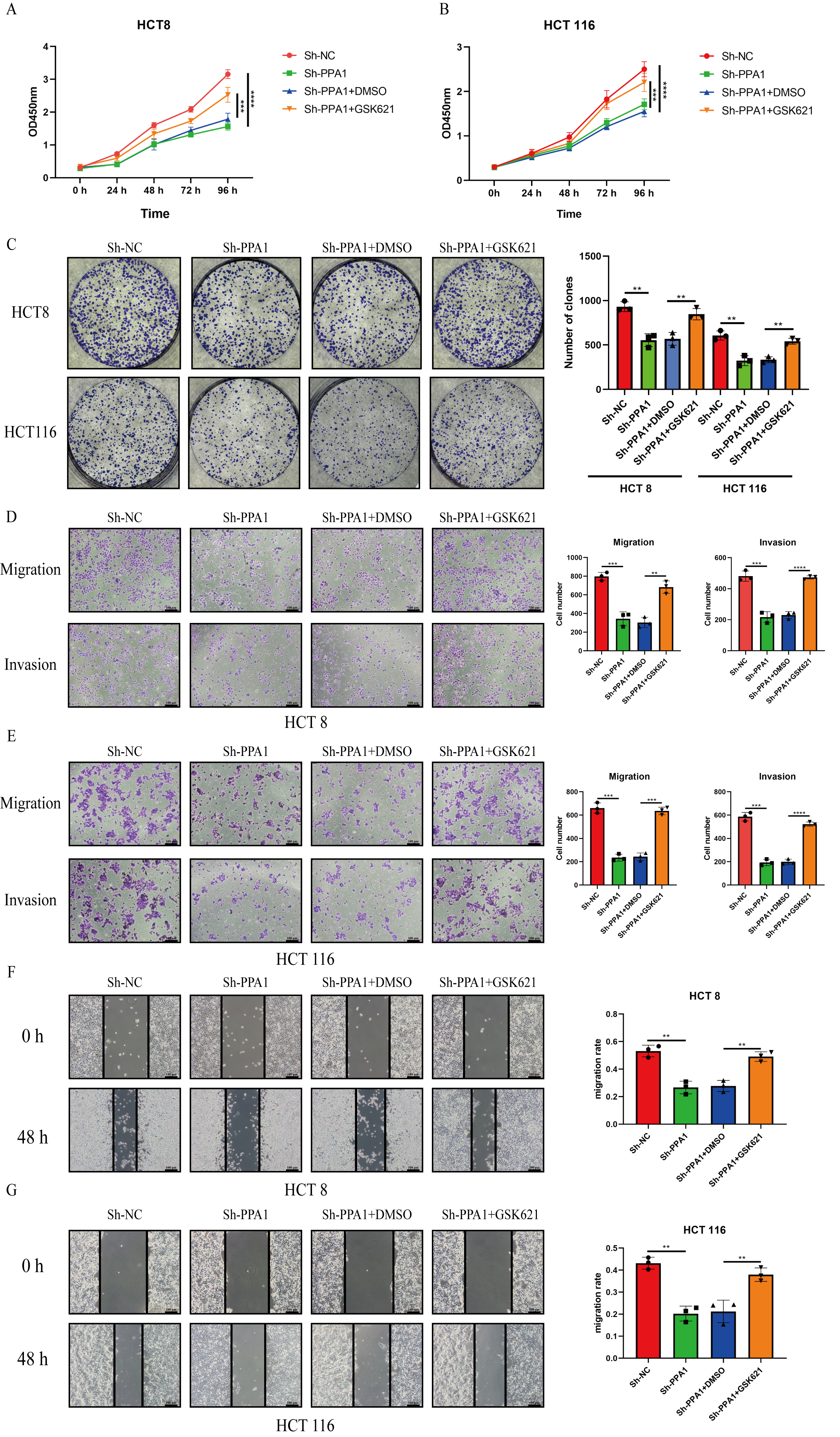

Supplement: Supplementary file 9 — Figure S6 [file 41420_2025_2816_MOESM9_ESM.tif]
